# Incidence of Coffee Leaf Rust in Vietnam, Possible Original Sources and Subsequent Pathways of Migration

**DOI:** 10.3389/fpls.2022.872877

**Published:** 2022-04-05

**Authors:** Cham Thi Mai Le, Izumi Okane, Yoshitaka Ono, Yoshiaki Tsuda, Yuichi Yamaoka

**Affiliations:** ^1^Graduate School of Life and Environmental Sciences, University of Tsukuba, Tsukuba, Japan; ^2^Division of Microbial Technology, Biotechnology Center of Ho Chi Minh City, Ho Chi Minh City, Vietnam; ^3^Faculty of Life and Environmental Sciences, University of Tsukuba, Tsukuba, Japan; ^4^College of Education, Ibaraki University, Mito, Japan; ^5^Sugadaira Research Station, Mountain Science Center, University of Tsukuba, Ueda, Japan

**Keywords:** coffee leaf rust disease, genetic diversity, *Hemileia vastatrix*, ITS, Southeast Asia

## Abstract

This research focused on the incidence and population genetics of coffee leaf rust (CLR) fungus, *Hemileia vastatrix*, to estimate the possible original source(s) and subsequent migration pathways of wind-borne and human-aided spores in three main coffee production regions (Northwest, Central Highlands, and Southeast) in Vietnam. In southern Vietnam (Central Highlands and Southeast), *Coffea canephora* covers the majority area, while Catimor lines of *C. arabica* accounts for 95% of the coffee plantations in northwestern Vietnam. Field surveys conducted at eighty-five plantations, show coffee leaf samples infected by the rust fungus across forty-one plantations. Catimor varieties exhibited high levels of susceptibility with severe rust symptoms, while robusta varieties had varying degrees of susceptibility. We analyzed 863−869 base pairs of internal transcribed spacer (ITS) region from 83 samples (41 sequences from Vietnam, 2 from Thailand, and the remaining 40 from American countries); and fifty-two haplotypes consisting of 123 polymorphic sites were detected. Although the analysis of molecular variance (AMOVA) indicates significant genetic differentiation in the *H. vastatrix* populations in Vietnam, there was no clear genetic structure with respect to the three geographic areas surveyed. Based on the haplotype network, NeighborNet analysis, and geographical distribution patterns of the haplotypes, five haplotypes were identified as early established, from which most other haplotypes in Vietnam were derived. The early established haplotypes were found in the highest frequency in Northwest Vietnam. This finding corresponds to the earliest record of CLR in Vietnam. The phylogenetic network analysis also illustrated that *H. vastatrix* had expanded from the northwest to southern Vietnam. Pairwise genetic distance analysis and the geophylogenetic tree also suggests that CLR was first established in the Northwest. In addition, some scattered individuals on the principal coordinate analysis (PCoA) diagram and several separated haplotypes in the phylogenetic networks indicated that other branches of CLR in Vietnam were initiated in the Central Highlands. *Hemileia vastatrix* from these branches have been spreading in southern Vietnam.

## Introduction

The rust fungi belong to the order Pucciniales (Class: Pucciniomycetes, Division: Basidiomycota). They are obligate parasites of vascular plants and cause rust diseases in several important crops worldwide (Kolmer et al., 2009). Management of rust disease is difficult because the life cycle of most rust fungi is complicated, with up to five different spore stages, including basidiospore, spermatium, aeciospore, urediniospore, and teliospore, which may occur on the same host (autoecious life cycle) or on two taxonomically unrelated hosts (heteroecious life cycle) (Cummins and Hiratsuka, 2003; Kolmer et al., 2009). Among them, *Hemileia* (Zaghouaniaceae P. Syd. & Syd.) (Aime and McTaggart, 2020) is characterized by its unique uredinial anamorph (Ritschel, 2005) and comprises 42 species, found mainly in plants of Rubiaceae and Apocynaceae families. Among these plants, species of *Coffea* (Rubiaceae) produce coffee beans—the most valuable and traded tropical agricultural product, which is severely affected by the rust disease (Talhinhas et al., 2017). Even if coffee trees do not die from early defoliation by heavy rust infections, the fungal infection will cause a severe decrease in berry yield and quality. This disease in coffee trees causes a rapid decline in the trees’ vigor and makes them more vulnerable to other pathogenic fungi and pests in successive years. CLR has been recorded to cause approximately 35% yield losses, resulting in annual losses of one to two billion US dollars (Talhinhas et al., 2017). Therefore, it is essential to develop effective and practical measures to manage the CLR disease.

The best strategy to control this disease is by using resistant varieties (Silva et al., 2006). Hibrido de Timor (HdT) is a natural hybrid between *Coffea arabica* and *C. canephora*, which was resistant to all races of the coffee rust fungus, *Hemileia vastatrix*, in the 1950s. The hybrids Catimor and Sachimor have also been considered resistant to *H. vastatrix* (Talhinhas et al., 2017). Planting disease-resistant coffee varieties was considered an effective strategy for preventing or decreasing the infection of *H. vastatrix* for a long time. However, in recent years, there have been reports of the outbreak of CLR in most coffee-growing areas worldwide, including regions planting rust-resistant varieties (Maia et al., 2013; Cabral et al., 2016; Talhinhas et al., 2017). Thus, to develop effective and practical management programs, we must know the current pathogenic diversity of the rust populations, understand the underlying genetic variations, and predict the tendency for variations in future.

Currently, *H. vastatrix* and *H. coffeicola* Maubl. and Roger are the known causative agents of the CLR disease (Thirumalachar and Narasimhan, 1947; Ritschel, 2005; Talhinhas et al., 2017). *Hemileia vastatrix* is the major agent causing substantial yield loss in worldwide coffee production. On the contrary, *H. coffeicola* occurs only in West and Central Africa on *C. canephora* (Ritschel, 2005). Previously, the global outbreaks of CLR have always been related to *H. vastatrix*. The native range of the fungus is suggested to be a geographic range spanning the Great Lake region, Ethiopia, and the eastern half of the Congo River basin in East Africa (McCook, 2019). After the domestication of *C. arabica* in Yemen in the fifteenth century, the fungus and its cultivated host had spread through Ceylon and southern India in 1869. The reasons for the first explosion of CLR were monsoon, shipment of infected coffee seedlings or goods, and the attached rust spores on the clothes of British soldiers returning from Ethiopia. Thereafter, the CLR pandemic exploded in the Indian Ocean basin and the Pacific from 1875 to 1920. Besides other routes related to wind, trade, and communication, the migration of coffee planters from infected areas to the Indian Ocean basin and the Pacific could also be considered the causal factor. Wind, infected materials, and anthropogenic activities also explain the CLR outburst in West Africa during 1950−1970 and in America during 1970−1989 (McCook, 2006, 2019).

*Coffea arabica* was first cultivated in northern Vietnam in 1857 by French missionaries (D’haeze et al., 2004; Anonymous, 2019). Some decades after that, in October 1890, *H. vastatrix* was first collected on *C. arabica* in some plantations (Hennings, 1895). Several years later, *C. arabica* spread to Southern Vietnam. However, when diseases, including coffee rust, caused yield losses in the coffee plantations, *C. canephora* was introduced to replace *C. arabica*, and it rapidly spread in the southern parts of Vietnam in the early 1900s (D’haeze et al., 2003). In the meantime, the Northwest of Vietnam also switched to Catimor derivatives due to its ability to resist *H. vastatrix* (Anonymous, 2019). After World War II, the introduction of *C. canephora* cultivars enabled Vietnam to become the world’s second-largest coffee producer, after Brazil. There are three main coffee-growing regions in Vietnam—the Southeast, the Central highlands, and the Northwest. Topographic and climatic differences affect seasonal disease occurrences and their severity in the three major coffee-growing regions. Annually, the disease begins from March to April and from July to September in the northern part of Vietnam. By contrast, the rust disease symptoms become apparent in the wet season between April and September in the southern part. CLR has been reported periodically in Vietnam. However, the actual occurrence and distribution of the disease in Vietnam and the origin of CLR have not been investigated. Therefore, the objectives of this research were to (i) investigate the occurrence of CLR in the main coffee-producing regions in Vietnam, (ii) evaluate the current genetic diversity and population structure of the coffee rust fungus based on sequencing of internal transcribed spacer (ITS) region, and (iii) estimate the geographic region where *H. vastatrix* first established as well as the migration direction of CLR between main coffee-growing areas in Vietnam.

## Materials and Methods

### Field Investigation and Sample Collection

Coffee species were identified based on morphology (flower, bract, apex, leaf, fruit, etc.), according to the descriptions of Backer and Bakhuizen Van Den Brink (1965). Three coffee regions, including the Southeast (Dong Nai and Binh Phuoc provinces), the Central Highlands (Dak Lak, Dak Nong, and Lam Dong provinces), and the Northwest (Son La and Dien Bien provinces), were investigated for the occurrence of CLR. The accessed plantations were along the street and were more than 1 km apart (Supplementary Figure S1). In each plantation, two rows of trees around the edges were examined for *H. vastatrix*. Ten coffee leaves with orange-colored rust sori were collected from three trees at each collection site, packed in a plastic bag, and referred to as one sample. Photographs of the rust-infected leaves were also taken, and the severity of the coffee leaf rust estimated using the method of Belan et al. (2020). The leaves were then dried by pressing them between dry blotting papers and maintained at room temperature (25 ± 2°C) at the microbiology laboratory of the Biotechnology Center of Ho Chi Minh City. Dried specimens were then deposited in the Mycological Herbarium of the University of Tsukuba (TSH), Japan. In addition to the Vietnamese samples, two coffee leaf specimens from Thailand, deposited in TSH (TSH-R59309 and TSH-R59420), were also used for this research. All samples were collected between July and November in 2019, except for one sample collected in November 2018 (Table 1).

**TABLE 1:** Coffee leaf rust samples collected in Vietnam and Thailand.

Area	Province	Date of collection	Latitude	Longitude	Specimen ID	TSH No.*	Severity %**			Level of severity**
							Sample 1	Sample 2	Sample 3	
The Southeast	Dong Nai	12 July 2019	10.983694	107.253583	S1_2	TSH-R30018	15.4	15.4	13.3	5
			10.946722	107.313111	S1_5	TSH-R30022	1.2	0.5	0.9	1–2
			10.857889	107.162611	S1_9	TSH-R30024	2.1	6.1	3.3	3–4
			10.972861	107.076528	S1_11	TSH-R30028	3.2	2.1	1.9	2–3
			11.242361	107.390583	S1_14	TSH-R30031	15.4	15.4	17	5–6
			11.266639	107.379417	S1_15	TSH-R30034	1.2	2.1	1.2	2–3
			10.770278	107.263861	S1_18	TSH-R30036	17	8.4	13.3	5–6
	Binh Phuoc	15 June 2019	11.70625	107.052972	S2_6	TSH-R30042	8.4	1.2	2.1	2–5
			11.741583	107.145611	S2_7	TSH-R30043	8.4	4.5	2.1	3–5
			11.800778	107.217472	S2_14	TSH-R30045	8.4	15.4	7.9	4–5
			11.796333	107.21725	S2_15	TSH-R30046	2.1	1.2	2.1	2–3
The Central Highlands	Dak Lak	07 August 2019	12.553333	108.180972	H1_8	TSH-R30050	13.3	8.4	7.9	4–5
			12.551167	108.191139	H1_9	TSH-R30051	6.1	2.1	2.1	3–4
			12.621417	108.191972	H1_10	TSH-R30052	7.9	13.3	13.3	4–5
			12.667417	108.195972	H1-11	TSH-R30055	8.4	7.9	4.5	4–5
			12.7035	108.195667	H1_12	TSH-R30059	6.1	7.9	7.9	4
	Dak Nong	06 August 2019	12.567722	107.8235	H2_2	TSH-R30063	13.3	15.4	8.4	5
			12.495222	107.740111	H2_4	TSH-R30067	0.9	0.5	0.5	1
			12.471917	107.691639	H2_5	TSH-R30069	7.9	3.2	3.2	3–4
			12.181889	107.637944	H2_6	TSH-R30071	8.4	6.1	13.3	4–5
			12.155528	107.640194	H2_7	TSH-R30073	33	50.9	50.9	7
			12.061833	107.677111	H2_8	TSH-R30080	8.4	8.4	8.4	5
			11.983972	107.700444	H2_9	TSH-R30085	33	33	13.3	5–7
	Lam Dong	08 August 2019	11.737306	107.978917	H3_3	TSH-R30088	0.2	0.5	0.5	1
			11.776389	108.153722	H3_8	TSH-R30098	17	17	17.8	6
			11.78475	108.184194	H3_9	TSH-R30101	6.1	7.9	17	4–6
			11.762556	108.345389	H3_10	TSH-R30104	8.4	8.4	13.3	5
			11.623583	108.256444	H3_11	TSH-R30107	6.1	17	8.4	4–6
			11.628333	108.140944	H3_12	TSH-R30109	13.3	7.9	7.9	4–5
			11.558972	107.830917	H3_14	TSH-R30118	17	15.4	19.1	5–6
The Northwest	Son La	17 August 2019	21.238944	103.859944	N1_4	TSH-R30120	13.3	50.9	15.4	5–7
			21.259306	103.868722	N1_5	TSH-R30125	19.1	50.9	33	6–7
			21.35375	103.812389	N1_7	TSH-R30129	23.4	50.9	8.4	5–7
			21.517722	103.643694	N1_8	TSH-R30133	15.4	19.1	15.4	5–6
			21.524444	103.637528	N1_9	TSH-R30137	19.1	33	19.1	6–7
			21.506333	103.653444	N1_10	TSH-R30145	13.3	4.5	23.4	4–7
			21.538667	103.620167	N1_11	TSH-R30148	7.9	19.1	33	4–7
	Dien Bien	18 August 2019	21.565306	103.465083	N2_1	TSH-R30155	13.3	6.1	19.1	4–6
			21.514972	103.206028	N2_4	TSH-R30160	17	8.4	13.3	5–6
			21.503917	103.196889	N2_5	TSH-R30167	13.3	6.1	33	4–7
			21.503361	103.202167	N2_6	TSH-R30172	23.4	4.5	13.3	4–7
Thailand	Chiang Mai	26 November 2018	18.767	99.02616	TL_18TH13	TSH-R59309	NT	NT	NT	NT
		07 November 2019	13.75398	100.50144	TL_19TH46	TSH-R59420	NT	NT	NT	NT

*TSH: Mycological herbarium of University of Tsukuba, Japan.

**Belan et al., 2020.

### DNA Extraction, Internal Transcribed Spacer Region Amplification, and Sequencing

DNA extraction from herbarium specimens requires special methods, in which only a small amount of spores must be picked to avoid possible cross-contamination. The thermal-shock method given by Fraczek et al. (2019) was used for DNA extraction, and BPS buffer was substituted with buffer-1 (10 mM Tris-Cl pH 8.3, 1.5 mM MgCl_2_, 50 mM KCl). The protocol can be summarized as follows: A coffee leaf with fully matured, seemingly clean sori on the lower surface was chosen for DNA extraction from each sample (one sample consisted of ten leaves from three different trees). Urediniospores were collected from several pustules on a single leaf and immersed in a 200 μl tube containing 30 μl buffer-1. The tubes were then vortexed and incubated at 95°C for 15 min and immediately transferred to a deep freezer (–80°C) for 10 min. Subsequently, the spore suspension was defrosted at room temperature (25 ± 2°C) and centrifugated at 15,000 rpm for 1 min. Finally, the supernatant was transferred to a new 200 μl tube. The nucleic acid concentration and 260/280 and 260/230 ratios were estimated using a DS-11 spectrophotometer (DeNovix, Wilmington, DE, United States).

The ITS region was amplified using ITS5 and ITS4 primers (White et al., 1990). A 25 μl reaction mixture containing 1–2 μl DNA template (30−50 ng/μl), 0.2 mM of each primer (2.5 μl), 12.5 μl Gene RED PCR Mix Plus (Nippon Gene, Tokyo, Japan), and 5.5–6.5 μl autoclaved distilled water, were prepared. Polymerase chain reaction (PCR) was conducted using a TaKaRa PCR Thermal Cycler Dice^®^ Touch (TaKaRa, Tokyo, Japan). The PCR process was as follows: initial denaturation at 95°C for 5 min, followed by 35 cycles of denaturation at 95°C for 1 min, annealing at 54°C for 30 s, and extension at 72°C for 1 min, followed by a final extension at 72°C for 10 min.

The amplification was confirmed using electrophoresis. The amplified products were purified using FastGene™ Gel/PCR extraction kit (Nippon Genetics, Tokyo, Japan) and consigned to Eurofins Genomics (Tokyo, Japan) for sequencing. The obtained sequences were edited and assembled using ATGC software (Genetyx Co., Tokyo, Japan). Finally, the sequences were aligned and compared with other sequences in the National Center for Biotechnology Information database using the BLASTn program^1^.

### Population Genetic Analysis

Besides sequences from Vietnam and two sequences from Thailand, 40 ITS sequences of *H. vastatrix* from a few Central and South American countries (Mexico, Brazil, and Colombia), and those from Portugal’s Coffee Rust Research Centre (CIFC: Centro de Investigação de Ferrugens do Cafeeiro) were downloaded from the NCBI Genbank (Supplementary Table S1) and added to the data, for identifying the original source(s) of CLR in Vietnam. Rust fungus samples from the same coffee-growing area (province or city) in different coffee-growing regions (Northwest, Central Highlands, and Southeast in Vietnam, and Thailand) were designated as a single population. Rust samples represented by ITS sequences downloaded from GenBank were treated similarly. Totally, 20 populations of *H. vastatrix* were examined in this study, including Southeast-Dong Nai, Southeast-Binh Phuoc, Central Highlands-Dak Lak, Central Highlands-Dak Nong, Central Highlands-Lam Dong, Northwest-Son La, and Northwest-Dien Bien populations from Vietnam; Chiang Mai population from Thailand; Veracruz, Chiapas, Oaxaca, and Puebla populations from Mexico; Minas Gerais-Coimbra, Minas Gerais-Capinopolis, Minas Gerais-São Sebastião do Paraíso, Minas Gerais-Senhora de Oliveira, and Espírito Santo-Venda Nova do Imigrante populations from Brazil; Quindío-Buenavista and Caldas-Chinchiná populations from Colombia; and the CIFC population from Portugal.

The obtained sequences were aligned using Clustal X version 2.0 (Larkin et al., 2007) (Supplementary Material S4). The number of haplotypes, haplotype diversity (Hd) (Nei and Li, 1979), and nucleotide diversity (Pi) of each population were calculated using the Dnasp program version 6 (Librado and Rozas, 2009). To visualize the correlation between nucleotide diversity and geographical pattern, the regression statistics between nucleotide diversity and latitude was analyzed using PAST v4.03 (Hammer et al., 2001), and Microsoft Excel was used to generate a scatter diagram. The demographic pattern in each population and in the whole *H. vastatrix* population was evaluated by conducting Tajima’s neutrality test (Tajima, 1989) using Arlequin version 3.5 (Excoffier and Lischer, 2010).

To evaluate the genetic structure of *H. vastatrix* populations, analyses of molecular variance (AMOVA) within each population and among different populations were calculated using GenAlEx 6.51b2 (Peakall and Smouse, 2012). Next, the matrices of pairwise genetic distance (Φ_PT_) (Weir and Cockerham, 1984) between populations and individuals based on the AMOVA were calculated using GenAlEx 6.51b2. These matrices were used as input data for principal coordinate analysis (PCoA) to visualize the relationships between populations and the genetic structure of the whole *H. vastatrix* population.

To evaluate the genetic relationship among the haplotypes and to predict the ancestors of the populations in Vietnam, we employed parsimony and NeighborNet network approaches (Bryant and Moulton, 2013). Firstly, Dnasp program version 6 was used to generate a raw nexus file that was manually prepared to form an input trait file for PopART software version 1.7 (Leigh and Bryant, 2015). Next, a Median-joining haplotype network was constructed using PopART version 1.7. Meanwhile, the NeighborNet network (Bryant and Moulton, 2013) was generated using Splitstree 5 software (Huson and Bryant, 2006). Moreover, a map-based phylogenetic tree was generated to estimate the migration routes and the influence of geographic patterns on the similarities or differences among the *H. vastatrix* populations. First, a map figure was generated using GeoMapApp 3.6.12^2^ (Ryan et al., 2009). Next, a phylogenetic tree of the *H. vastatrix* populations was built using the program MEGA X (Kumar et al., 2018), based on the maximum likelihood statistical method with 1000 bootstrap replications, Generalized time-reversible model (Tavaré, 1986) with the rate among sites Gamma distributed with invariant sites (G + I) was used. Lastly, a 3D graphical interface of the phylogeographic tree was generated by combining data from the map, phylogenic tree, and locations of collected samples using GenGIS2 software (Parks et al., 2013).

## Results

### Field Investigation

**FIGURE 1:**
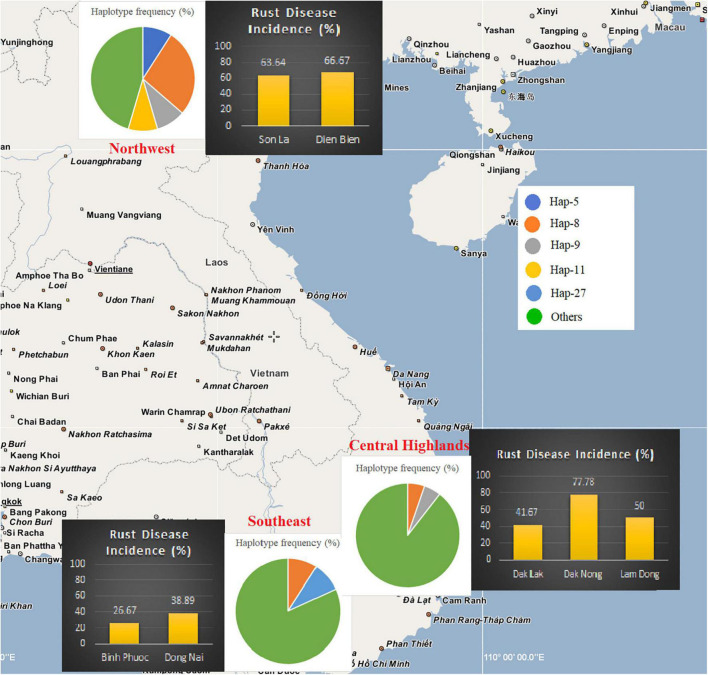
Rust disease incidence and the frequency of ancestral *Hemileia vastatrix* haplotypes in different geographical sample areas of Vietnam. Disease incidence in the areas of each province is indicated using bar charts and *Hemileia vastatrix* haplotype frequency in the sampling areas is shown using pie charts.

Field investigations showed that *C. canephora* is commonly planted in southern Vietnam, while *C. arabica* cv. Catimor is planted in the Northwest (Table 1 and Supplementary Table S2). In the Southeast of Vietnam, thirty-three plantations in Dong Nai and Binh Phuoc provinces were investigated, of which 26.7% (4/15) plantations in Binh Phuoc and 38.9% (7/18) plantations in Dong Nai were infected by the rust fungus. Simultaneously, thirty-five plantations in the Central Highlands were investigated. The incidence of coffee rust disease in the three provinces was variable, i.e., 41.7% (5/12) in Dak Lak, 77.8% (7/9) in Dak Nong, and 50.0% (7/14) in Lam Dong province. Our investigation at 17 plantations in the mountainous area of North Vietnam (Northwest) revealed that coffee rust disease incidence in this region was high—63.6% (7/11) and 66.7% (4/6) in Son La and Dien Bien provinces, respectively. After thorough field investigations at 85 coffee plantations in Vietnam, a total of 41 CLR samples were collected from 41 plantations in the three regions (Table 1, Figure 1, Supplementary Figure S1, and Supplementary Table S2).

The severity of coffee leaf rust on *C. arabica* in the Northwest and *C. canephora* in the Central Highlands and Southeast of Vietnam was assessed using individual survey sites and the method described by Belan et al. (2020) (Supplementary Figure S2 and Table 1). Overall, the severity of the disease in Vietnam ranged from 1–7. Coffee plantations in Son La and Dien Bien provinces (Northwest) had the highest rust severity (up to level 7). While in southern Vietnam the disease appeared less severe than that in northern Vietnam, except at two plantations in Dak Nong province (Central Highlands); however, these two plantations had been abandoned many years prior to our sampling. All plantations in Lam Dong province had moderate rust disease severity (levels 4-6) except plantation 3 (level 1) which is in a high mountainous area. The severity of rust disease in Southeast Vietnam was found to be lower than in the other areas, except for plantations 14 and 18 in Dong Nai province (levels 5-6) where coffee is intercropped with black pepper. Arabica varieties at all sites in the Northwest exhibited high susceptibility as the number and size of the rust lesions was greater when compared with other regions, while robusta varieties in the Central Highlands and the Southeast exhibited varying degrees of susceptibility. It was noticeable that those in Dak Nong and Dong Nai were highly variable among survey sites, ranging from highly resistant to highly susceptible (Table 1 and Supplementary Figure S2).

### Population Genetic Analysis of *Hemileia vastatrix*

The DNA sequences of the ITS region from all samples were 863−869 bp and had 98–100% similarity with *H. vastatrix* (Supplementary Table S3) (Genbank accession numbers: LC682363−LC682405). From the 86 sequences (41 sequences from Vietnam), 123 polymorphic (segregating) sites (77 singleton variable sites and 46 parsimony informative sites) were detected. In total, 52 haplotypes (36 haplotypes from Vietnam) were identified (Table 2), with 0.952 ± 0.00022 Hd and 0.00779 nucleotide diversity (Pi). The nucleotide diversity was highest (0.02651) in the Southeast (Binh Phuoc population), and the genetic variation was absent in the Veracruz (Mexico) population. Populations from Dak Nong (Central Highlands), Lam Dong (Central Highlands), and Dong Nai (Southeast) in Vietnam, and Chiang Mai in Thailand had higher diversity (0.02527, 0.01452, 0.01175, and 0.01153, respectively) (Supplementary Table S5) than the remaining populations. We detected a significant negative correlation between latitude and nucleotide diversity when we focused on the populations in Vietnam and Thailand (*p* < 0.05). The value of Tajima’s neutrality test (*D*) for the whole population of *H. vastatrix* was −0.19518. Although almost all populations in Vietnam had a negative value of Tajima’s *D* (except for the Central Highlands-Lam Dong population); however, the values were not significant (Supplementary Table S6).

**TABLE 2:** List of haplotypes obtained from sequences of coffee leaf rust fungus in Vietnam, Thailand, Portugal, and some American countries (Brazil, Colombia, Mexico).

Hap	Frequency	Distribution																				Sequence ID.
		ST-DN	ST-BP	CH-DL	CH-DN	CH-LD	NW-SL	NW-DB	TL-CM	MG-CB	ES-VNI	MG-CP	MG-SSP	MG-SO	Q-B	C-C	V	C	O	P	C-P	
**Hap_1**	1	0	0	1	0	0	0	0	0	0	0	0	0	0	0	0	0	0	0	0	0	30055
**Hap_2**	1	0	1	0	0	0	0	0	0	0	0	0	0	0	0	0	0	0	0	0	0	30045
**Hap_3**	1	0	0	0	1	0	0	0	0	0	0	0	0	0	0	0	0	0	0	0	0	30071
**Hap_4**	1	0	0	0	0	1	0	0	0	0	0	0	0	0	0	0	0	0	0	0	0	30109
**Hap_5**	1	0	0	0	0	0	1	0	0	0	0	0	0	0	0	0	0	0	0	0	0	30148
**Hap_6**	1	1	0	0	0	0	0	0	0	0	0	0	0	0	0	0	0	0	0	0	0	30036
**Hap_7**	1	0	0	0	0	0	0	0	1	0	0	0	0	0	0	0	0	0	0	0	0	59309
**Hap_8**	15	1	0	0	0	1	1	2	0	3	1	0	1	0	1	1	0	1	1	1	0	Mexico_6, 30160, 30172, Brazil_8, Brazil_1, Mexico_4, Mexico_8, Colombia_3, Brazil_5, Brazil_6, Colombia_4, Brazil_14, 30022, 30107, 30137
**Hap_9**	2	0	0	0	1	0	1	0	0	0	0	0	0	0	0	0	0	0	0	0	0	30120, 30073
**Hap_10**	1	0	0	0	0	0	0	1	0	0	0	0	0	0	0	0	0	0	0	0	0	30155
**Hap_11**	1	0	0	0	0	0	1	0	0	0	0	0	0	0	0	0	0	0	0	0	0	30133
**Hap_12**	1	0	0	0	0	0	1	0	0	0	0	0	0	0	0	0	0	0	0	0	0	30145
**Hap_13**	1	0	0	0	0	0	1	0	0	0	0	0	0	0	0	0	0	0	0	0	0	30129
**Hap_14**	1	0	0	0	0	0	0	1	0	0	0	0	0	0	0	0	0	0	0	0	0	30167
**Hap_15**	1	0	0	1	0	0	0	0	0	0	0	0	0	0	0	0	0	0	0	0	0	30052
**Hap_16**	1	1	0	0	0	0	0	0	0	0	0	0	0	0	0	0	0	0	0	0	0	30028
**Hap_17**	9	0	0	0	0	0	0	0	0	0	0	0	2	0	0	0	2	1	0	1	3	Mexico_3, Mexico_2, Mexico_1, Mexico_9, CIFC_1, CIFC_2, CIFCI_3, Brazil_15, Brazil_16
**Hap_18**	1	0	0	0	0	0	0	0	0	0	0	1	0	0	0	0	0	0	0	0	0	Brazil_12
**Hap_19**	1	0	0	0	0	0	0	0	0	0	0	0	0	1	0	0	0	0	0	0	0	Brazil_18
**Hap_20**	1	0	0	0	1	0	0	0	0	0	0	0	0	0	0	0	0	0	0	0	0	30069
**Hap_21**	1	0	0	0	0	0	0	0	0	0	1	0	0	0	0	0	0	0	0	0	0	Brazil_7
**Hap_22**	1	0	0	0	0	0	0	0	0	0	0	1	0	0	0	0	0	0	0	0	0	Brazil_11
**Hap_23**	1	0	0	0	0	0	0	0	0	0	0	0	0	1	0	0	0	0	0	0	0	Brazil_17
**Hap_24**	2	0	0	0	0	0	0	0	0	0	0	0	0	0	0	0	0	1	1	0	0	Mexico_5, Mexico_7
**Hap_25**	1	0	0	0	0	0	1	0	0	0	0	0	0	0	0	0	0	0	0	0	0	30125
**Hap_26**	1	0	0	0	0	0	0	0	0	0	0	0	0	0	1	0	0	0	0	0	0	Colombia_1
**Hap_27**	2	1	0	0	0	0	0	0	0	0	0	0	0	0	1	0	0	0	0	0	0	Colombia_2, 30018
**Hap_28**	1	0	0	0	0	0	0	0	0	1	0	0	0	0	0	0	0	0	0	0	0	Brazil_4
**Hap_29**	1	0	0	0	0	0	0	0	0	1	0	0	0	0	0	0	0	0	0	0	0	Brazil_2
**Hap_30**	1	0	0	0	0	0	0	0	0	0	1	0	0	0	0	0	0	0	0	0	0	Brazil_10
**Hap_31**	1	0	0	0	0	0	0	0	0	0	0	1	0	0	0	0	0	0	0	0	0	Brazil_13
**Hap_32**	7	0	0	0	0	0	0	0	0	1	0	0	0	0	3	3	0	0	0	0	0	Colombia_5, Brazil_3, Colombia_7, Colombia_8, Colombia_10, Colombia_9, Colombia_6
**Hap_33**	1	0	0	0	0	0	0	0	0	0	1	0	0	0	0	0	0	0	0	0	0	Brazil_9
**Hap_34**	1	0	1	0	0	0	0	0	0	0	0	0	0	0	0	0	0	0	0	0	0	30046
**Hap_35**	1	1	0	0	0	0	0	0	0	0	0	0	0	0	0	0	0	0	0	0	0	30031
**Hap_36**	1	1	0	0	0	0	0	0	0	0	0	0	0	0	0	0	0	0	0	0	0	30034
**Hap_37**	1	0	0	0	0	1	0	0	0	0	0	0	0	0	0	0	0	0	0	0	0	30098
**Hap_38**	1	0	0	0	1	0	0	0	0	0	0	0	0	0	0	0	0	0	0	0	0	30065
**Hap_39**	1	1	0	0	0	0	0	0	0	0	0	0	0	0	0	0	0	0	0	0	0	30024
**Hap_40**	1	0	1	0	0	0	0	0	0	0	0	0	0	0	0	0	0	0	0	0	0	30042
**Hap_41**	1	0	1	0	0	0	0	0	0	0	0	0	0	0	0	0	0	0	0	0	0	30043
**Hap_42**	1	0	0	1	0	0	0	0	0	0	0	0	0	0	0	0	0	0	0	0	0	30050
**Hap_43**	1	0	0	1	0	0	0	0	0	0	0	0	0	0	0	0	0	0	0	0	0	30059
**Hap_44**	1	0	0	0	0	1	0	0	0	0	0	0	0	0	0	0	0	0	0	0	0	30104
**Hap_45**	1	0	0	0	0	0	0	0	1	0	0	0	0	0	0	0	0	0	0	0	0	59420
**Hap_46**	1	0	0	1	0	0	0	0	0	0	0	0	0	0	0	0	0	0	0	0	0	30051
**Hap_47**	1	0	0	0	0	1	0	0	0	0	0	0	0	0	0	0	0	0	0	0	0	30088
**Hap_48**	1	0	0	0	1	0	0	0	0	0	0	0	0	0	0	0	0	0	0	0	0	30080
**Hap_49**	1	0	0	0	1	0	0	0	0	0	0	0	0	0	0	0	0	0	0	0	0	30067
**Hap_50**	1	0	0	0	0	1	0	0	0	0	0	0	0	0	0	0	0	0	0	0	0	30118
**Hap_51**	1	0	0	0	0	1	0	0	0	0	0	0	0	0	0	0	0	0	0	0	0	30101
**Hap_52**	1	0	0	0	1	0	0	0	0	0	0	0	0	0	0	0	0	0	0	0	0	30085

*ST-DN, Southeast-Dong Nai; ST-BP, Southeast-Binh Phuoc; CH-DL, Central Highlands-Dak Lak; CH-DN, Central Highlands-Dak Nong; CH-LD, Central Highlands-Lam Dong; NW-SL, Northwest-Son La; NW-DB, Northwest-Dien Bien; TL-CM, Thailand-Chiang Mai; MG-CB, Minas Gerais-Coimbra; ES-VNM, Espírito Santo-Venda-Nova-do-Imigrante; MG-CP, Minas Gerais- Capinopolis; MG-SSP, Minas Gerais-Sao-Sebastiao-do-Paraiso; MG-SO, Minas Gerais-Senhora-de-Oliveira; Q-B, Quindío-Buenavista; C-C, Caldas-Chinchiná; V, Veracruz; C, Chiapas; O, Oaxaca; P, Pueblo; C-P, Centro de Investigação de Ferrugens do Cafeeiro-Portugal.*

**FIGURE 2:**
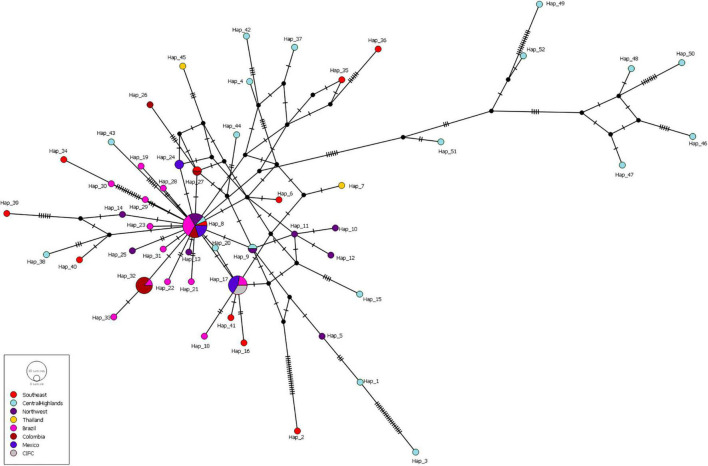
Median-joining haplotype network inferred from the ITS regions of *Hemileia vastatrix* sampled from Vietnam, Thailand, Portugal, and other countries in America (Brazil, Colombia, Mexico). Circles represent haplotypes, with bright red corresponding to samples collected from Southeast Vietnam, light turquoise the Central Highlands of Vietnam, purple the Northwest of Vietnam, while yellow indicates Thailand, pink Brazil, brick red Colombia, dark blue Mexico, and grey CIFC (Portugal). The size of the circles is proportional to the number of sequences in each haplotype, one transverse line represents simple mutations, and black nodes are non-sampled haplotypes.

Haplotype diversity of most of the populations was high (> 0.900) and did not show any geographic patterns, except the Caldas-Chinchiná population from Colombia, which showed a much lower value (0.500) than others (Supplementary Table S5). When we focused on the haplotype distribution, the most common and major haplotype was Hap 8 (18.1%; detected in all populations except for the Thailand population), followed by Haps 17 [10.8%; Mexico (Veracruz, Chiapas, and Puebla), CIFC-Portugal, Brazil (Minas Gerais-São Sebastião do Paraíso)], Hap 32 [8.4%; Colombia and Brazil (Minas Gerais-Coimbra)], and three haplotypes, namely, Hap 9 (the Northwest and the Central Highlands of Vietnam), Hap 24 [Mexico (Chiapas and Oaxaca)], and Hap 27 (Colombia and the Southeast of Vietnam). The remaining haplotypes were singleton (Table 2 and Figure 2).

The AMOVA showed that genetic differentiation among the twenty *H. vastatrix* populations from the six countries (Vietnam, Thailand, Brazil, Colombia, Mexico, and Portugal) was low (*F_ST_* = 0.175) but significant (*p* < 0.01). Only 17% of the total variation was found among populations, while 83% of the differences were among individuals within a population (Table 3). The genetic differentiation (Φ_PT_) between the Vietnamese–Thai and Central and South American populations, and the Brazilian and Caldas-Chinchiná population (Colombia) was high (Supplementary Table S7). Meanwhile, the genetic differentiation between the Mexican and Vietnamese–Thai populations was lower than that observed between other populations (Supplementary Table S7). The PCoA chart indicated that the Vietnamese–Thai and the Mexican populations (Puebla, Chiapas, Veracruz, and Oaxaca) had closer genetic distance than combinations with the Brazilian–Colombian populations (Supplementary Figure S3).

**TABLE 3:** Analysis of molecular variance (AMOVA) based on ITS sequences of the *Hemileia vastatrix* populations.

Source of variation	d.f.	Sum of squares	Variance components	Percentage of variation
Among populations	19	210.921	1.257	17
Within populations	63	374.055	5.937	83
Total	82	584.976	7.195	100
Fixation Index (*F*_ST_)	0.175			
P value	0.001			

*d.f., degrees of freedom.*

**FIGURE 3:**
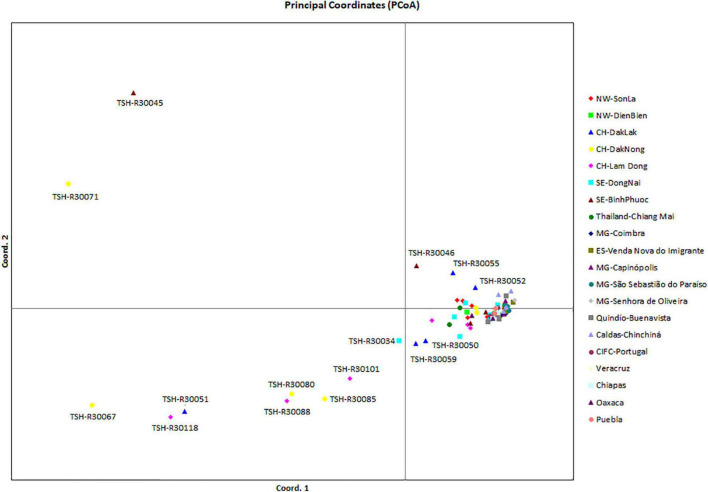
Principal coordinate analysis (PCoA) of the genetic (nucleotide) diversity of *Hemileia vastatrix* in Vietnam, Thailand, Portugal, and some countries in the Americas (Brazil, Colombia, Mexico). Individuals that are closer together have smaller genetic distances. NW: Northwest (Vietnam), CH: Central Highands (Vietnam), ST: Southeast (Vietnam), CIFC: Centro de Investigação de Ferrugens do Cafeeiro (Portugal), MG: Minas Gerais (Brazil), ES: Espírito Santo (Brazil).

**FIGURE 4:**
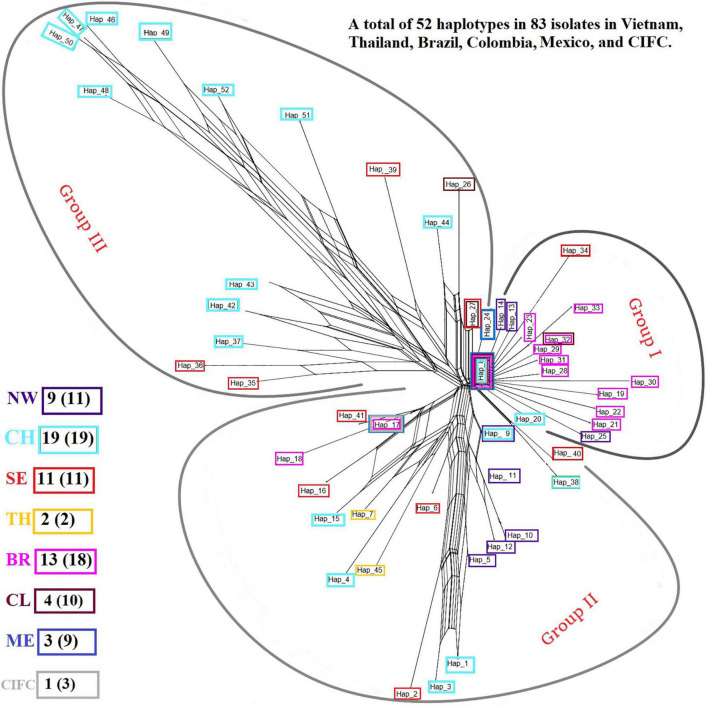
NeighborNet graph constructed from the pairwise FST values of *Hemileia vastatrix* samples from Vietnam, Thailand, Portugal, and other American countries (Brazil, Colombia, Mexico) based on their ITS regions. The haplotypes detected in seven areas are framed in boxes with different colors: purple indicates the Northwest (NW) of Vietnam, light turquoise the Central Highlands (CH) of Vietnam, bright red the Southeast (SE) of Vietnam, while yellow indicates Thailand (TH), pink Brazil (BR), brick red Colombia (CL), blue Mexico (ME), and grey the Centro de Investigação de Ferrugens do Cafeeiro (CIFC)-Portugal. After the abbreviation of each region the number of haplotypes detected in each area is stated, and the number in the parentheses indicates the number of sequences obtained from the region.

Analysis of the genetic structure of the whole *H. vastatrix* population (by studying the genetic distance between individuals) revealed that most individuals were closely gathered, while some from the Central Highlands and Southeast were distantly scattered (TSH-R30034, 30045, 30046, 30051, 30067, 30071, 30080, 30085, 30088, 30101, and 30118) (Figure 3). Haplotype (Figure 2) and NeighborNet (Figure 4) networks showed three groups in the *H. vastatrix* populations. The first group included Haps 13, 14, 19, 20, 21, 22, 23, 25, 28, 29, 30, 31, 32, 33, and 34, which were derived from Hap 8, and were mostly from Central and South American countries and the Northwest of Vietnam. There were 19 haplotypes in group 2, namely, Haps 1, 2, 3, 4, 5, 6, 7, 9, 10, 11, 12, 15, 16, 17, 18, 38, 40, 41, and 45 from Vietnam, Thailand, Brazil, and CIFC-Portugal. The remaining haplotypes (Haps 26, 27, 35, 36, 37, 39, 42, 43, 44, 46, 47, 48, 49, 50, 51, and 52) were included in the third group and were mainly from the Central Highlands and the Southeast of Vietnam.

**FIGURE 5:**
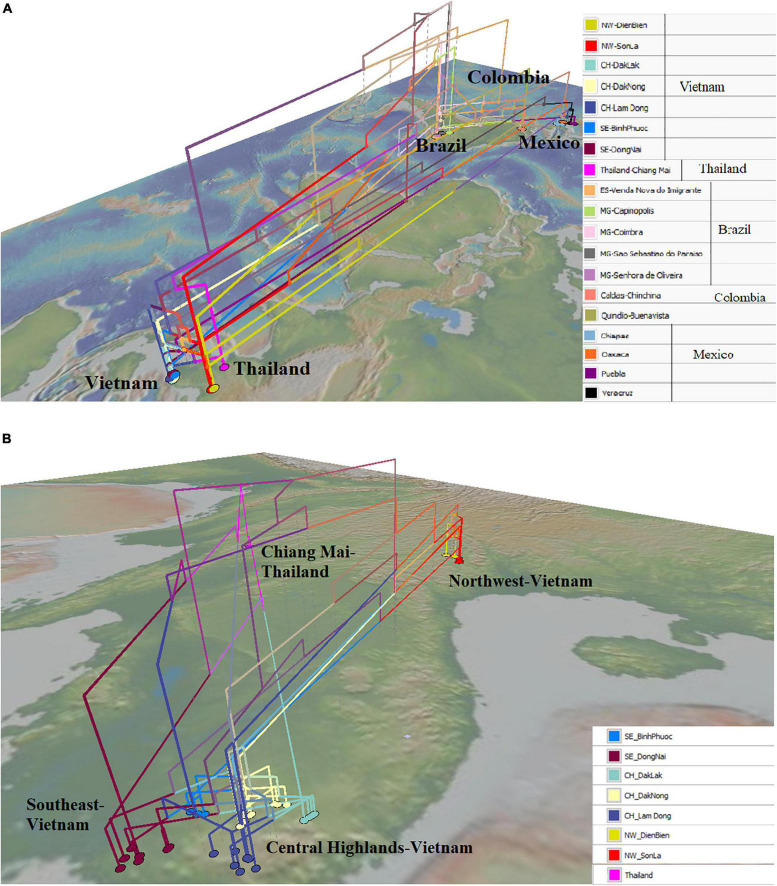
Geophylogeny of *Hemileia vastatrix* from Vietnam, Thailand, and other American countries (Brazil, Colombia, Mexico) using ITS sequences. Colored nodes on the map correspond to sampling locations. **(A)** Geophylogeny of *Hemileia vastatrix* from Vietnam, Thailand, and Central and South American countries, **(B)** Geophylogeny of *Hemileia vastatrix* from areas in Vietnam and Chiang Mai-Thailand. NW: Northwest (Vietnam), CH: Central Highands (Vietnam), ST: Southeast (Vietnam), CIFC: Centro de Investigação de Ferrugens do Cafeeiro (Portugal), MG: Minas Gerais (Brazil), ES: Espírito Santo (Brazil).

The map-based phylogenetic tree highlighted the close genetic relationship of some populations in Mexico and Brazil with the Northwest (Vietnam) populations. Populations from Oaxaca, Chiapas, and Puebla (Mexico) were directly related to populations from Son La and Dien Bien (Northwest of Vietnam) (Figure 5A). Besides that, some individuals from Minas Gerais and Espírito Santo (Brazil) had close relationships with some individuals from the Northwest of Vietnam (Figure 5A). On the contrary, a population from Oaxaca had a closer relationship with some individuals from Dong Nai (Southeast of Vietnam) (Figure 5A). Figure 5B shows the close relationship of the Chiang Mai (Thailand) population with populations in the Central Highlands and the Southeast (Dong Nai) of Vietnam. Moreover, all populations in the Central Highlands and the Southeast were connected with the Northwest populations (Figure 5B).

## Discussion

### Catimor Derivatives of Coffee in Vietnam Are No Longer Resistant to Rust Disease?

The results of field investigations show that the leaf rust disease is present in all coffee growing regions in Vietnam, from South to North, and from low altitudes to high mountainous areas. These observations suggest that all coffee species and cultivars planted in Vietnam now are susceptible to rust disease. Previously, *Coffea arabica* cv. Catimor, HdT, and Sachimor were known to be rust-resistant (Talhinhas et al., 2017). However, our survey shows that this cultivar can be infected by rust fungus. The number and size of the rust lesions was large on the coffee tree leaves in the Northwest plantations, where Catimor varieties are predominately planted (Table 1 and Supplementary Figure S2). The detection of rust-infected leaves in regions planting rust-resistant coffee varieties is not unique to this research; numerous previous researches have revealed that the rust disease existed in HdT growing areas of Brazil and Colombia (Maia et al., 2013; Cabral et al., 2016; Talhinhas et al., 2017). This raises concerns about whether all rust-resistant coffee varieties have become susceptible to *H. vastatrix* or whether the situation is only present in a few countries or areas. The most probable reasons for the loss of rust resistance are mutations in the genome of *H. vastatrix* or the appearance of new pathogen races (Talhinhas et al., 2017). However, other factors that could affect the rust resistance of these coffee varieties must also be studied because climatic conditions and cultural practices can also promote disease outbreaks (Avelino et al., 2015). For example, there were high levels of rust severity found in the abandoned coffee plantations of the Central Highlands (Table 1 and Supplementary Figure S2). Therefore, further research on such factors is required to control CLR disease in Vietnam.

### *Hemileia vastatrix* in Vietnam Has Low Genetic Divergence and Unstructured Population but High Haplotype Diversity

**FIGURE 6:**
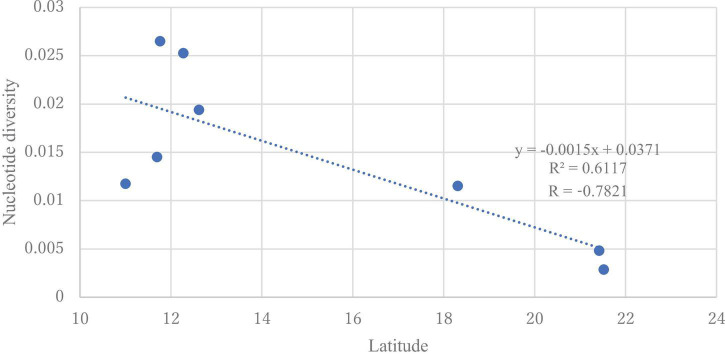
Linear regression model for the latitude and nucleotide diversity of *Hemileia vastatrix* populations from Vietnam and Thailand. The dotted line represents the regression line with equation *y* = −0.0015x + 0.0371.

Genetic diversity and population structure analyses disclosed some actualities of the *H. vastatrix* populations in Vietnam. Firstly, the genetic differentiation of *H. vastatrix* in Vietnam is low, as shown in the calculated AMOVA (Table 3 and Supplementary Table S5). The percentage of variation within populations surpasses that among populations (Table 3), indicating that the genetic divergence among populations is small. Moreover, the nucleotide diversity values of populations in Vietnam are low (less than 0.1; Supplementary Table S5). Secondly, *H. vastatrix* populations in Vietnam are unstructured. The PCoA diagram (Figure 3) illustrates that some individuals are scattered from others. Dispersed haplotypes are also well illustrated as they are divided into groups distinguished from two others (Figures 2, 4); however, these groups are divided without relation to the regions where CLR samples were collected. Lastly, the Hd of *H. vastatrix* in Vietnam is high. This is not only because of their high values showed in Supplementary Table S5 but also because many groups of haplotype are divided into networks (Figures 2, 4). Besides that, the linear regression (Figure 6) shows that the nucleotide diversity has a negative relationship with latitude; the higher the latitude, the lower the nucleotide diversity value. Most haplotypes in Vietnam are unique. Mutations in the *H. vastatrix* populations of Vietnam are likely to be spontaneous without being affected by geographical factors or the host’s resistance genes. Nonetheless, random alterations due to environmental changes, such as warmer climate or increased annual rainfall, could result in the formation of new virulent races (Ali et al., 2014). Furthermore, the unstructured population of *H. vastatrix* could result from asexual reproduction (Ramírez-Camejo et al., 2021). The results of this study are similar to those from studies in the coffee-growing regions of Peru and Brazil (Maia et al., 2013; Cabral et al., 2016; Quispe-Apaza et al., 2017, 2021).

### The Hypothesized Original Sources and the Migration Direction of *Hemileia vastatrix* in Vietnam

Based on the population genetic analyses, a hypothetical interpretation of the first establishment of CLR disease and the successive spore transportation pathways through coffee-growing areas in Vietnam is possible. The frequency of Haps 5, 8, 9, 11, 17, 27, and 32 in the whole *H. vastatrix* population and their positions in the phylogenetic analyses (Figures 1, 2, 4) indicate that these haplotypes are shared in most populations and steady in the reproductive process. Thus, these haplotypes are referred to as the ‘early established haplotypes’. Among them, Hap 32 originated from Hap 8; and Haps 11 and 5 are descendants of Hap 9. Haps 8, 17, 27, and 32 are shared between Brazil, Colombia, CIFC-Portugal, and Mexico. In addition, the pairwise genetic distance matrices (Supplementary Table S7), PCoA diagram (Supplementary Figure S3), and phylogeographic tree (Figure 5A) highlight the close genetic distances and genetic similarities between some populations in Mexico and Brazil and the Northwest population in Vietnam. These results prove that *H. vastatrix* populations from Vietnam (mostly from the Northwest) and those from Central and South American countries (Mexico and Brazil) have very close relationships. However, we cannot ascertain that the *H. vastatrix* population in Vietnam spread from these countries just because the first rust detection in Vietnam was in 1890, while the first confirmation of rust establishment in Brazil and Mexico was in the 1970s and 1980s, respectively (McCook, 2006, 2019). On second thought, *H. vastatrix* might have migrated to Vietnam from African countries because Vietnam and these countries were colonized by the French government during World War I. *Hemileia vastatrix* was first identified in Vietnam in 1890, the exact timeline of the global CLR pandemic (1875−1920). At that time, rust-infected coffee seedlings or materials from infected areas in Africa (where *H. vastatrix* is native) might have been carried to Vietnam by the French. However, confirming the origin of CLR in Vietnam necessitates further studies with larger rust samples from other countries, especially African countries. For these reasons, this discussion only focused on the first establishment of CLR in the three main coffee growing regions in Vietnam. Three main inferences regarding the origin and migration route of *H. vastatrix* can be gathered based on the present study. Firstly, because Φ_PT_ was previously measured only based on the genetic drift (Hedrick, 2005), the low genetic distance recorded between populations in Vietnam (Supplementary Table S7 and Supplementary Figure S3) proves that the probability of migration between these regions was high. Secondly, most ancestral haplotypes (Haps 5, 8, 9, 11) are present in the Northwest, and the percentage of those ancestral haplotypes in the Northwest is larger than that in other areas (Figure 1). Thirdly, the phylogeographic tree shows that *H. vastatrix* from Brazil and Mexico have genetic similarity with populations in the Northwest of Vietnam (Figure 5A); all populations from southern Vietnam had a close genetic relationship with the Northwest population (Figure 5B). Therefore, we can infer that *H. vastatrix* existed in the north of Vietnam in the early years of introducing coffee, then spread to the Southeast and the Central Highlands as coffee cultivation was extended to these areas. Although *C. arabica* was cut down for Catimor coffee in northern Vietnam (Anonymous, 2019), urediniospores of the rust fungus could still exist by parasitizing coffee plants in other plantations (the replacement of coffee varieties was not synchronized well and did not occur at the same time in all plantations of a region) or wild coffee plants and infected Catimor coffee, making this coffee variety susceptible to coffee rust. Hap 8 is one of the first ancestors that spread in northern Vietnam. After that, this haplotype became widespread in coffee plantations in the Northwest and formed Haps 13, 14, and 25. Later, it was transported to the South via human activities or infected materials and formed Haps 20, 27, and 34. Hap 27 is one of the ancestral haplotypes in the Southeast. Meanwhile, another ancestor, Hap 9, is widespread and formed Haps 5, 10, 11, and 12. This ancestor and some haplotypes (Haps 5 and 11) were then carried to the Central Highlands of Vietnam and formed Haps 1, 3, and 15 (Figures 2, 4). Thus, Haps 5, 8, 9, 11, and 27 are the ancestral haplotypes of the Vietnamese *H. vastatrix*.

On the other hand, data from the population genetic analyses (Figures 2, 3, 4) suggest that coffee rust in Vietnam has another origin besides spreading from the Northwest. This is indicated in the PCoA diagram (Figure 3): some individuals from the Central Highlands and the Southeast are scattered distantly from others. Figures 2, 4 also pointed out that some haplotypes (Haps 46–52) are genetically distant from others and form a different group. Information from the history of coffee in Vietnam consolidates this hypothesis. *Coffea canephora* was introduced in the Central Highlands of Vietnam to replace *C. arabica* in the early 1900s (D’haeze et al., 2003; Anonymous, 2019). When *C. canephora* spread to southern Vietnam, they might have also brought *H. vastatrix* to this region, broadened, and formed other branches of CLR in Vietnam. The appearance of absent haplotype (black node) in the haplotype network and the NeighborNet (Figures 2, 4) suggest that other haplotypes are yet to be discovered, and some of them are likely to be ancestral for haplotypes found in this study.

This study sheds new light on the genetic diversity of the CLR fungus, *H. vastatrix*, in Vietnam and in Central and South America. However, all analyses in this study are based on only one locus (ITS region). Because of this limitation, we could only discover two possible migration pathways of *H. vastatrix* in Vietnam. In future, we intend to conduct population genomic studies on *H. vastatrix* in Vietnam to reveal more information regarding the genetic structure, population demography, origins, and migration pathways of *H. vastatrix* in Vietnam.

## Data Availability Statement

The datasets presented in this study can be found in online repositories. The names of the repository/repositories and accession number(s) can be found in the article/Supplementary Material.

## Author Contributions

CL collected the samples, designed and performed the experiments, and wrote the manuscript. YO, IO, YT, YY, and CL conceived and directed the study. CL, YT, IO, and YO analyzed and biologically interpreted the data. YO, YT, and YY revised and approved the final version of the manuscript. All authors contributed greatly to the present manuscript.

## Author Disclaimer

All the specimens in this study were collected under the permission of the Vietnamese Government. In this study, all experiments were performed with the current laws of Japan.

## Conflict of Interest

The authors declare that the research was conducted in the absence of any commercial or financial relationships that could be construed as a potential conflict of interest.

## Publisher’s Note

All claims expressed in this article are solely those of the authors and do not necessarily represent those of their affiliated organizations, or those of the publisher, the editors and the reviewers. Any product that may be evaluated in this article, or claim that may be made by its manufacturer, is not guaranteed or endorsed by the publisher.

## Footnotes

1
https://blast.ncbi.nlm.nih.gov/Blast.cgi

2
www.geomapapp.org

## Supplementary Material

Supplementary Figure S1 Locations in Vietnam and Thailand where coffee leaf rust samples were collected. The 3D map was generated using Microsoft Excel. Purple dots represent plantations without rust disease and blue dots represent plantations with rust disease.

Supplementary Figure S2 Coffee leaf rust samples collected from *Coffea canephora* and *Coffea arabica* cv. Catimor in Vietnam.

Supplementary Figure S3 Principal coordinate analysis (PCoA) of *Hemileia vastatrix* distance matrix between populations. Populations that are closer together have smaller divergence. NW, Northwest; CH, Central Highlands; ST, Southeast; CIFC, Centro de Investigação de Ferrugens do Cafeeiro; MG, Minas Gerais; ES, Espírito Santo.

Supplementary Table S1 Internal transcribed spacer (ITS) sequences for coffee leaf rust fungus dowloaded from Genbank NCBI.

Supplementary Table S2 Coffee leaf rust samples collected in Vietnam and Thailand.

Supplementary Table S3 Information including query cover, % identity, name, and accession of the query sequences after using the NCBI BLAST tool for Vietnamese and Thailand coffee leaf rust fungus sequences.

Supplementary Table S5 Number of haplotypes, haplotype diversity, and nucleotide diversity of the *Hemileia vastatrix* populations.

Supplementary Table S6 Tajima’s D value for the *Hemileia vastatrix* populations.

Supplementary Table S7 Genetic differentiation (measured by ϕPT) of the *Hemileia vastatrix* populations between Vietnam and other countries.

Supplementary Material S4 Alignment dataset used for the population genetic analyses.
