# Supplementary material for: Incidence of Coffee Leaf Rust in Vietnam, Possible Original Sources and Subsequent Pathways of Migration
Source: Front Plant Sci. 2022 Apr 5;13:872877. doi: 10.3389/fpls.2022.872877 (PMC9016365; doi:10.3389/fpls.2022.872877)
Supplement: Supplementary Figure S1 — Locations in Vietnam and Thailand where coffee leaf rust samples were collected. The 3D map was generated using Microsoft Excel. Purple dots represent plantations without rust disease and blue dots represent plantations with rust disease. [file Data_Sheet_1.docx]

Supplementary Material

# Supplementary Figures, Tables, and Material

## Supplementary Figures


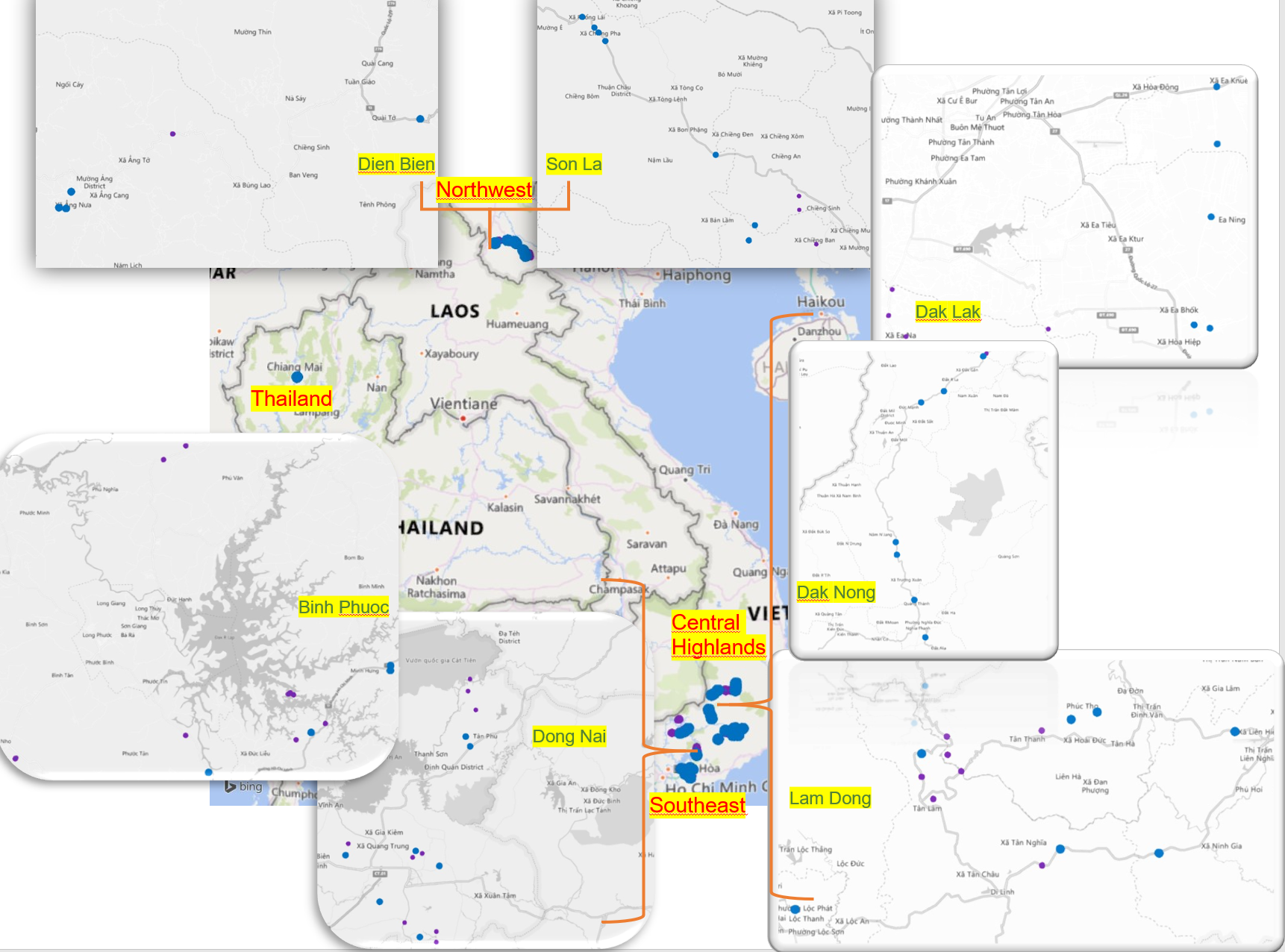


**Supplementary Figure S1.** Locations in Vietnam and Thailand where coffee leaf rust samples were collected. The 3D map was generated using Microsoft Excel. Purple dots represent plantations without rust disease and blue dots represent plantations with rust disease.


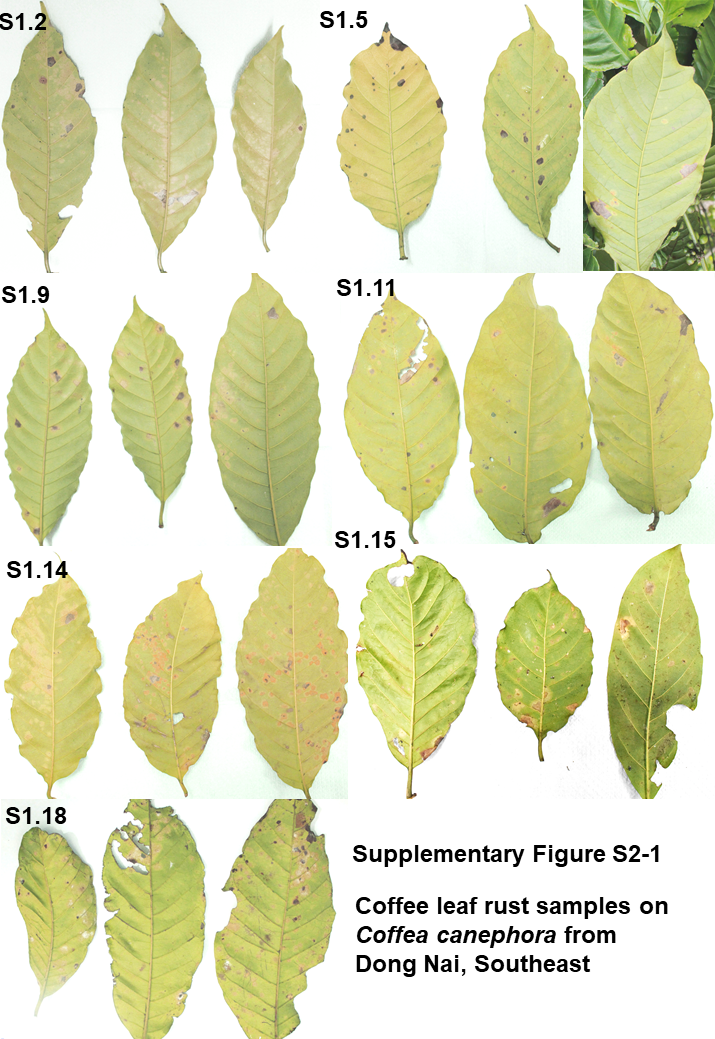


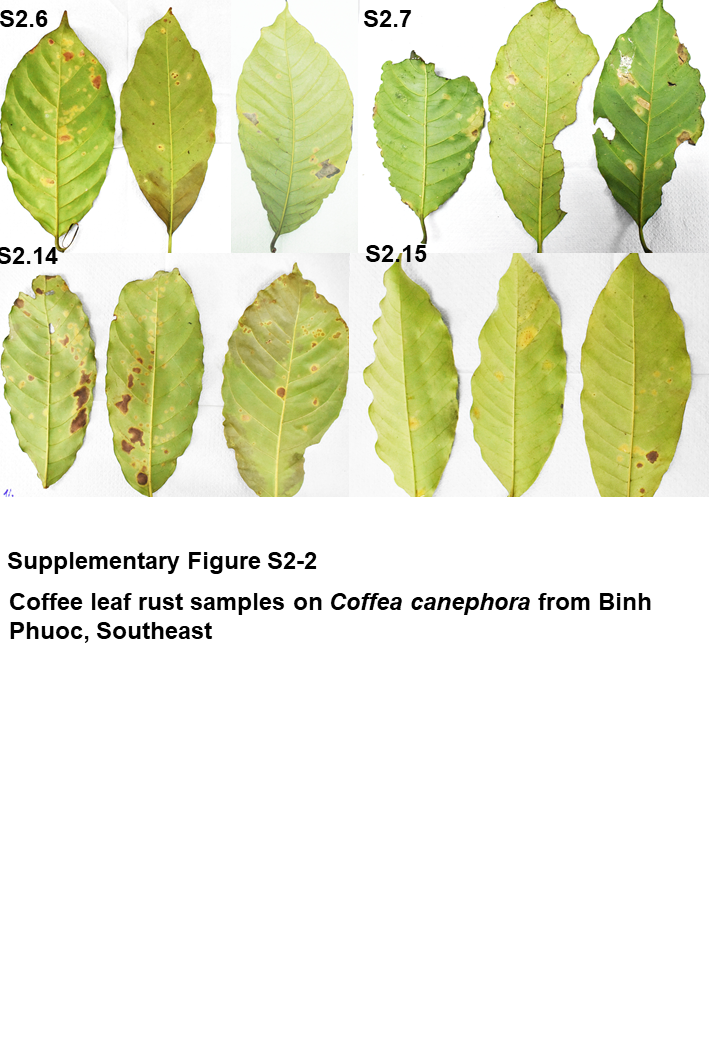


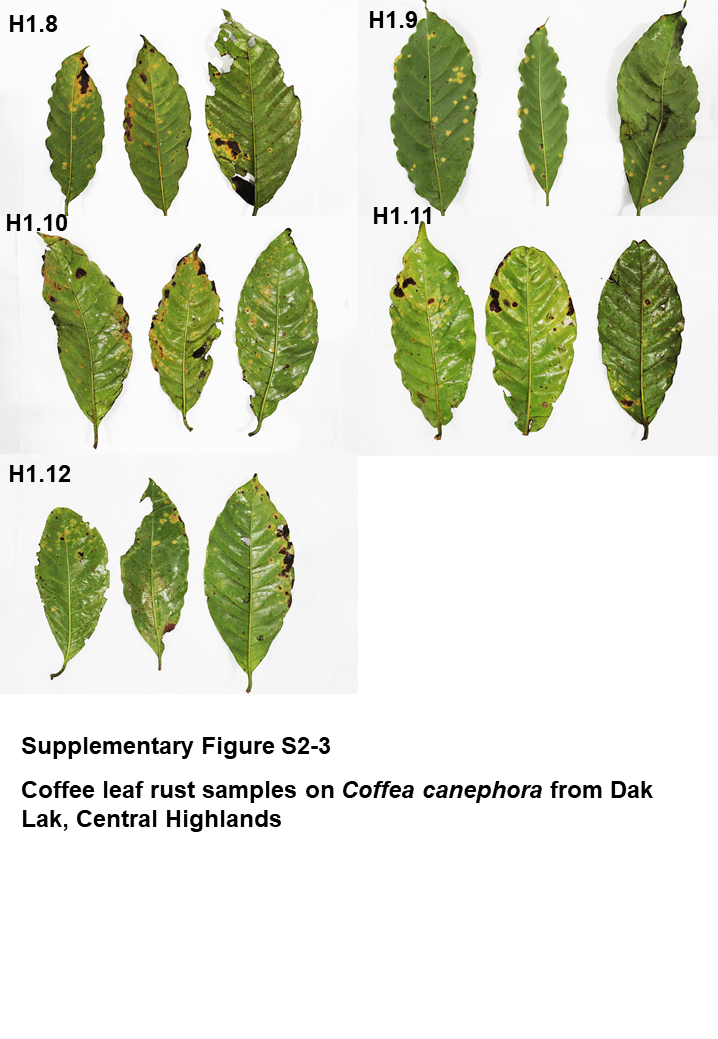


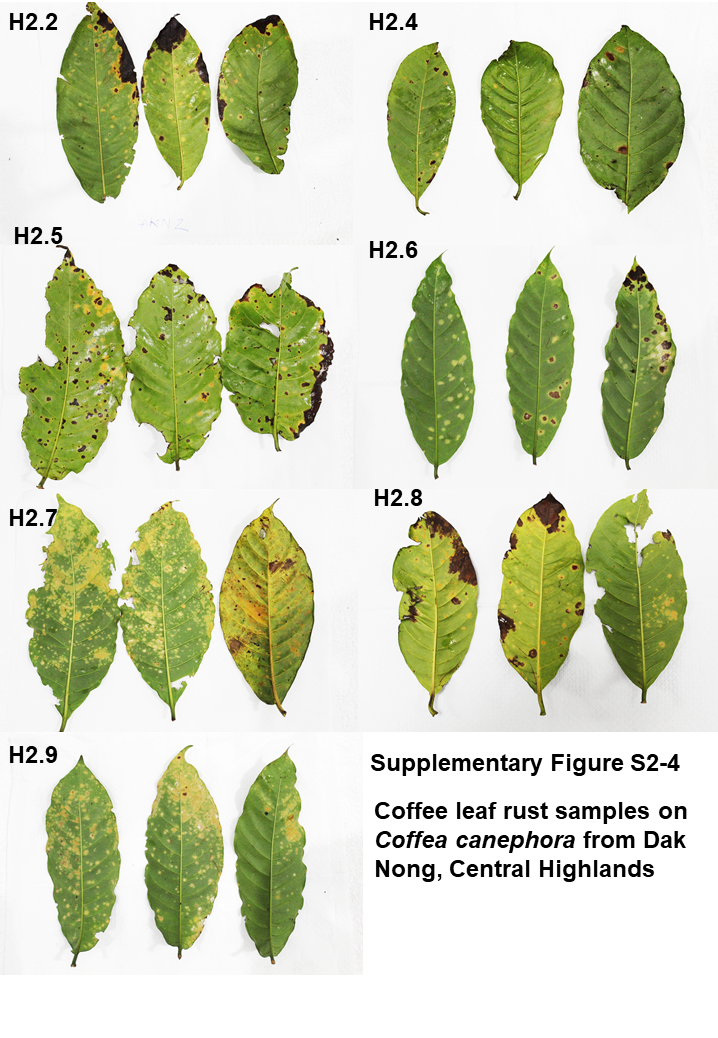


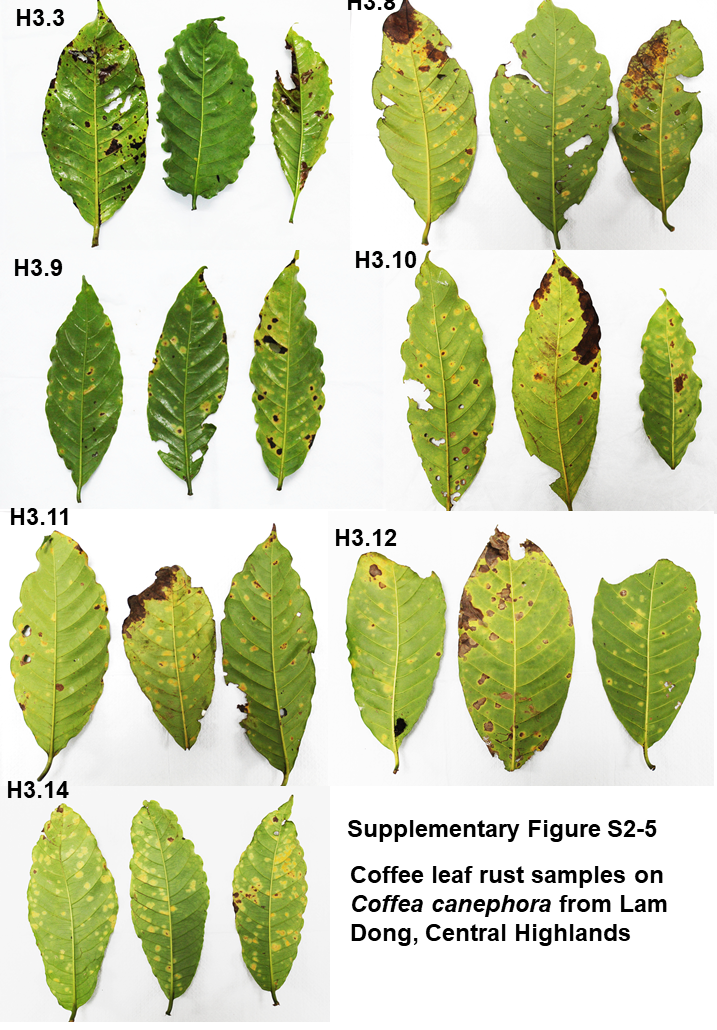


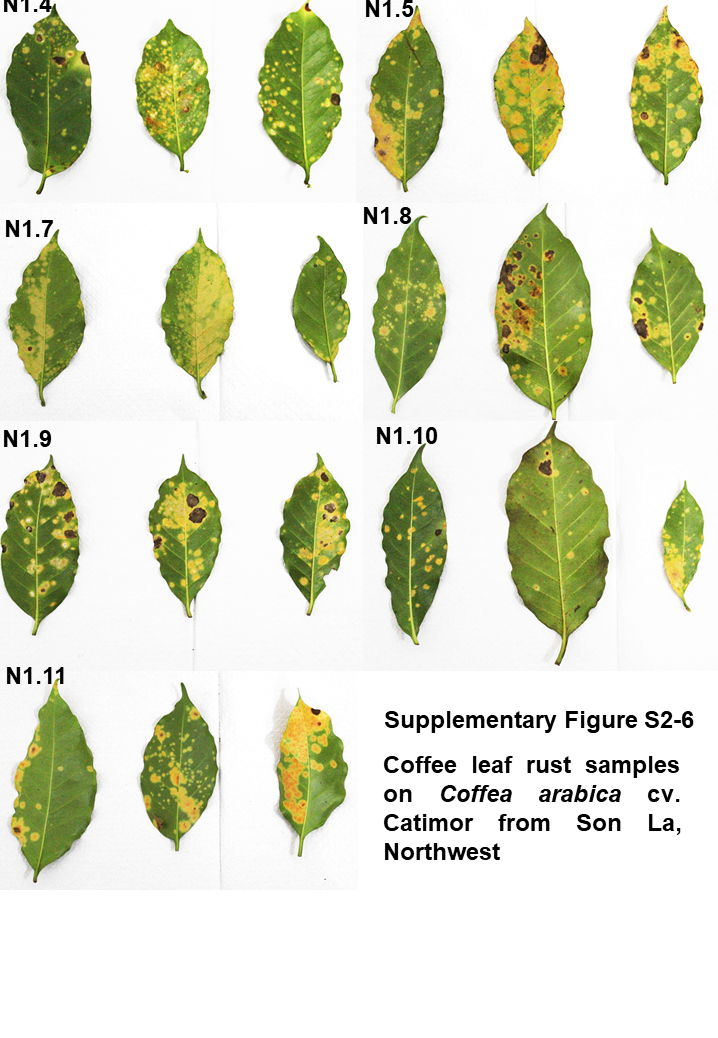


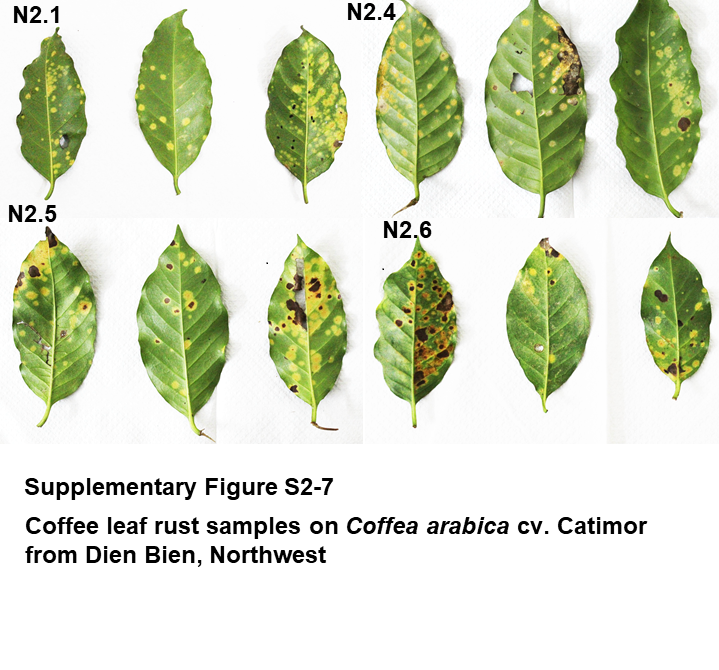


**Supplementary Figure S2.** Coffee leaf rust samples collected from *Coffea canephora* and *Coffea arabica* cv. Catimor in Vietnam.


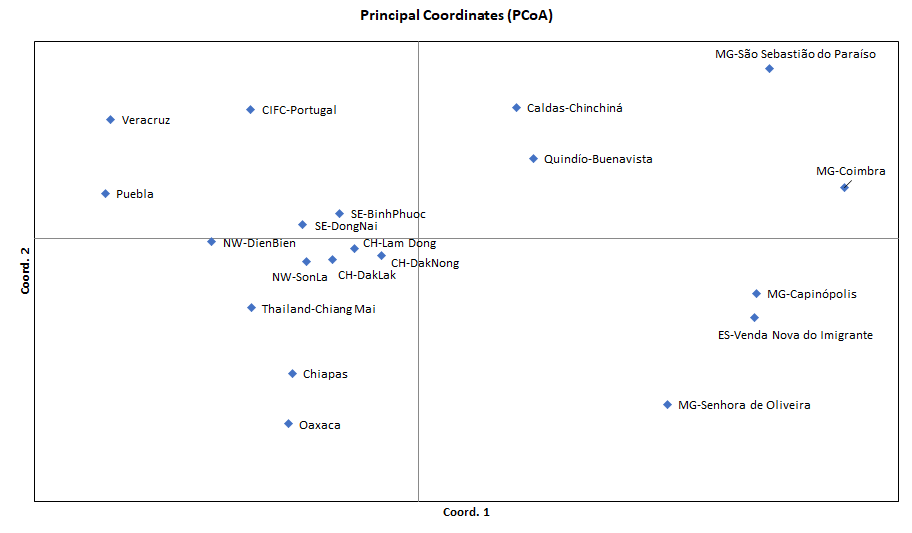


**Supplementary Figure S3.** Principal coordinate analysis (PCoA) of the *Hemileia vastatrix* distance matrix between populations. Populations that are closer together have smaller divergence. NW: Northwest (Vietnam), CH: Central Highands (Vietnam), ST: Southeast (Vietnam), CIFC: Centro de Investigação de Ferrugens do Cafeeiro (Portugal), MG: Minas Gerais (Brazil), ES: Espírito Santo (Brazil).

## Supplementary Tables

**Supplementary Table S1.** Internal transcribed spacer (ITS) sequences for coffee leaf rust fungus dowloaded from Genbank NCBI.

**Supplementary Table S2.** Coffee leaf rust samples collected in Vietnam and Thailand.

**Supplementary Table S3.** Information including query cover, % identity, name, and accession of the query sequences after using the NCBI BLAST tool for Vietnamese and Thailand coffee leaf rust fungus sequences.

**Supplementary Table S5.** Number of haplotypes, haplotype diversity, and nucleotide diversity of the *Hemileia vastatrix* populations.

**Supplementary Table S6.** Tajima's D value for the *Hemileia vastatrix* populations.

**Supplementary Table S7.** Genetic differentiation (measured by ΦPT) of the *Hemileia vastatrix* populations between Vietnam and other countries.

## Supplementary Material

**Supplementary Material S4.** Alignment dataset used for the population genetic analyses.
