# Supplementary material for: Incidence of Coffee Leaf Rust in Vietnam, Possible Original Sources and Subsequent Pathways of Migration
Source: Front Plant Sci. 2022 Apr 5;13:872877. doi: 10.3389/fpls.2022.872877 (PMC9016365; doi:10.3389/fpls.2022.872877)
Supplement: Supplementary Material S4 — Alignment dataset used for the population genetic analyses. [file Table_4.pdf]

**Supplementary Material S4**

Alignment dataset in the population genetic analyses

Incidence of coffee leaf rust in Vietnam, its origin and spread pathway

Cham Thi Mai Le 1,2, Izumi Okane 3, Yoshitaka Ono 4, Yoshiaki Tsuda 5,
Yuichi Yamaoka3

1 Graduate School of Life and Environmental Sciences, University of Tsukuba, Tsukuba, Ibaraki 305-8572, Japan
2 Division of Microbial Technology, Biotechnology Center of Ho Chi Minh City, Trung My Tay Ward, District 12, 71507 Ho Chi Minh, Vietnam
3 Faculty of Life and Environmental Sciences, University of Tsukuba, Tsukuba, Tsukuba, Ibaraki 305-8572, Japan
4 College of Education, Ibaraki University, Mito, Ibaraki 310-8512, Japan
5 Sugadaira Research Station, Montain Research Center, University of Tsukuba, Ueda, Nagano 386-2204, Japan

* Corresponding author. Graduate School of Life and Environmental Sciences, University of Tsukuba, Tsukuba, Ibaraki 305-8572, Japan
E-mail address: ltmcham.snn@tphcm.gov.vn (Cham Thi Mai Le)
s2030250@s.tsukuba.ac.jp

>TSH-R59309
TTAAGAGTGCACTTAATTGTGGCTTGAAATTTTACTTTATTTACCCCCAACGTC-
TTCGGGACACTGCGGC-
AATTTTTTGCTTACCGAATTTAACCCCTGCGGTTAGGCATATATAA-TTCTCTCT--
GAGGGTTGTATGTGTTCTAAT-CTTGTTTTTTTTATTTT-
CAACCACAAATTTATACATATGTATATATGTATT-ATTTACTATCAAGTAAATAAATAT-
AAAACTTTTAACAATGGATCTCTTGGCTCTCA-
CATCGATGAAGAACACAGTGAAATGTGATAAGTAATGTGAATTGCAGAATTCAGTGAATCAT
CGAATTTTTGAACGCATATTGCGCCTTTTGGCTATTCCAAAAGGTACACCTGTTTGAGAGTA
TGAAAGGTCAGGGTGTTGAGAGAGTTATTAAAAAAAA-
GAAAAAGGCAAAGTAACCCTTTAAGTGTTATTTTGTCTTTGATTTTTTTTTT-
CTCTTAGCATCTTGGATATTGGGTG-CTTGCCATTATTAAGTTTGATGG-
CTCACCTTAAATTTATAAGTTGTTTTTT-

ATTAAGGATGAAAAGTCTTTTGATGGCTTGATGTTATTGAATATATGAAATGTCATTCATCA
AGAAATCAGGGGGGTGACTAACCTTGATGAGAAATGCTGACTTTATAAACACATGTTAAAAG
ATAAATAAAAAGTAAAAGATAAAAAAAATAAAAAATAAAAAATTAAAGGGAAAGAAGTAAAAA
GAATGAGAGGTATGACATATTTTGAAAATATGTCCCTTG-
TTCTCTTTTTATTTTGATTATCTTTATTTTTTTAT-TTTTAATTTTAATTTTTATGTTTT-
AAGTTTTATTTTATTTTTTACTTCTTTATAATGAATCTCAAA
>TSH-R59420
TTAAGAGTGCACTTAATTGTGGCTTGAAATTTTACTTTATTTACCCCCAACGTC-
TTCGGGACACTGCGGC-
AATTTATTGCTTAGCGAATTTAACCCCGGCGGTTAGGCATATATAA-TTCTCTCT--
GAGGGTGGAATGGGTCCTAATCTT-GTTTTTTTTATTTT-
CAACCACAAATTTATACATATGTATATATGTATT-ATTTACTATCAAGTAAATAAATAT-
AAAACTTTTAACAATGGATCTCTTGGCTCTCA-
CATCGATGAAGAACACAGTGAAATGTGATAAGTAATGGGAATTGCAGAATTCAGTGAATCAT
CGAATTTTTGAACGCATATTGCGCCTTTTGGCTATTCCAAAAGGTACACCTGTTTGAGAGTA
TGAAAGGTCAGGGTGTTGAGAGAGTTATTAAAAAAAA-
GAAAAAGGCAAAGTAACCCTTTAAGTGTTATTTTGTCTTTGATTTTTTTTTT-
CTCTTAGCATCTTGGATATTGGGTG-CTTGCCATTATTAAGTTTGATGG-
CTCACCTTAAATTTATAAGTTGTTTTTT-
ATTAAGGATGAAAAGTCTTTTGATGGCTTGATGTTATTGA-
TATATGAAATGTCATTCATCAAGAAATCAGGGGGGTGACTAACCTTGATGAGAAATGTTGAC
TTTATAAACACATGTTAAAAGATAAATAAAAAGTAAAAGATAAAAAAAATAAAAAATTAAAAA
TTAAAGGGAAAGAAGTAAAAAGAATGAGAGGTATGACATATTTTGAAAATATGTCCCTTG-
TTCTCTTTTTATTTTGATTATCTTTATTTTTTTAT-TTTTAATTTTAATTTTTATGTTTT-
AAGTTTTATTTTATTTTTTACTTCTTTATAATGAATCTCAAA
>TSH-R30050
TTAAGAGTGCACTTAATTGTGGCTTGAAATTTTACTTTATTTACCCCCAACGTC-
TTCGGGACACTGCGGC-
AATTTTTTGTTTACCGAATTTAACCCCTGCGGTTAGGCATATATAT-TTCTCTCG--
GAGGGTTGTATGGTTTTTAATTCTTGTTTTTTTTATTTT-
CAACCACAAATTTATACATATGTATATATGTATT-ATTTACTATCAAGTAAATAAATAT-
AAAACTTTTAACAATGGATCTCTTGGCTCTCA-
CATCGATGAAGAACACAGTGAAATGTGATAAGTAATGTGAATTGCAGAATTCAGTGAATCAT
CGAATTTTTGAACGCATATTGCGCCTTTTGGCTATTCCAAAAGGTACACCTGTTTGAGAGTA
TGAAAGGTCAGGGTGTTGAGAGAGTTATTAAAAAAAA-
GAAAAAGGCAAAGTAACACTTTAAGTGTTATTTTGTCTTTGATTTTTTTTTT-
CTCTTAGCATCTTGGATATTGGGTG-CTTGCCATTATTAAGTTTGATGG-
CTCACCTTAAATTTATAAGTTGTTTTTT-
ATTAAGGATGAAAAGTCTTTTGATGGCTTGATGTTATTGA-

TATATGAAATGTCATTCATCAAGAAATCAGGGGGGTGACTAACCTTGATGAGAAATGTTGAC
TTTATAAACACATGTTAAAAGATAAATAAAAAGTAAAAGATAAAAAAAATAAAAAATTAAAAA
TTAAAGGGAAAGAAGTAAAAAGAATGAGAGGTATGACATATTTTGAAAATATGTCCCTTG-
TTCTCTTTTTATTTTGATTATCTTTATTTTTTTAT-TTTTAATTTTAATTTTTATGTTTT-
AAGTTTTATTTTATTTTTTACTTCTTTATAATGAATCTCAAA
>TSH-R30051
TTAAGAGTGCACTTAATTGTGGCTTGAAATTTTACTTAATTTCCACCCAACGCC-
TTCGGGACACTGCGGC-
AATTTATGGTTTAGCGAATTTAACCCCGGCGGTTAGGCATATATAA-TTCTCTCT--
GAGGGTGGTAGGGGTTCTAATCTT-GTTTTTTTTATTTT-
CAACCCCAAATTTATACATATGTATATATGTATT-ATTTACTATCAAGTAAATAAATAT-
AAAACTTTTAACAATGGATCTCTTGGCTCTCA-
CATCGATGAAGAACACAGTGAAATGTGATAAGTAATGTGAATTGCAAAATTCAGTGAATCAT
CGAATTTTTGAACGCATATTGCGCCTTTTGGCTATTCCAAAAGGTACACCTGTTTGAGAGTA
TGAAAGGTCAGGGTGTTGAGAGAGTTATTAAAAAAAA-
GAAAAAGGCAAAGTAACCCTTTAAGTGTTATTTTGTCTTTGATTTTTTTTTT-
CTCTTAGCATCTTGGATATTGGGTG-
CTTGCCATTATTAAGTTTGATGGGCTCACCTTAAATTTATAAGTTGTTTTTT-
ATTAAGGATGAAAAGTCTTTTGATGGCTTGATGTTATTGA-
TATATGAAATGTCATTCATCAAGAAATCAGGGGGGTGACTAACCTTGATGAGAAATGTTGAC
TTTATAAACACATGTTAAAAGATAAATAAAAAGTAAAAGATAAAAAAAATTAAAAATTAAAAA
TTAAAGGGAAAGAAGTAAAAAGAATGAGAGGTATGACATATTTTGAAAATATGTCCCTTG-
TTCTCTTTTTATTTTGATTATCTTTATTTTTTTATATTTTAATTTTAATTTTTATGTTTT-
AAGTTTTATTTTATTTTTTACTTCTTTATAATGAATCTCAAA
>TSH-R30052
TTAAGAGTGCACTTAATTGTGGCTTGAAATTTTACTTTATTTACACCCAACGTC-
TTCGGGACACTGCGGC-
AATTTATTGCTTAGCGAATTTAACCCCTGCGGTTAGGCATATATAA-TTCTCTCT--
GAGGGTTGTATGTGTTCTAATCTT-GTTTTTTTTATTTT-
CAACCCCAAATTTATACATTTGTATATATGTATT-ATTTACTATCAAGTAAATAAATAT-
AAAACTTTTAACAATGGATCTCTTGGCTCTCA-
CATCGATGAAGAACACAGTGAAATGTGATAAGTAATGTGAATTGCAGAATTCAGTGAATCAT
CGAATTTTTGAACGCATATTGCGCCTTTTGGCTATTCCAAAAGGTACCCCTGTTTGAGAGTA
TGAAAGGTCGGGGTGTTGAGAGAGTTATTAAAAAAAA-
GAAAAAGGCAAAGTAACCCTTTAAGTGTTATTTTGTCTTTGATTTTTTTTTT-
CTCTTAGCATCTTGGATATTGGGTG-CTTGCCATTATTAAGTTTGATGG-
CTCACCTTAAATTTATAAGTTGTTTTTT-
ATTAAGGATGAAAAGTCTTTTGATGGCTTGATGTTATTGA-
TATATGAAATGTCATTCATCAAGAAATCAGGGGGGTGACTAACCTTGATGAGAAATGCTGAC

TTTATAAACACATGTTAAAAGATAAATAAAAAGTAAAAGATAAAAAAAATAAAAAATAAAAAA
TTAAAGGGAAAGAAGTAAAAAGAATGAGAGGTATGACATATTTTGAAAATATGTCCCTTGGT
TCTCTTTTTATTTTGATTATCTTTATTTTTTTAT-TTTTAATTTTAATTTTTATGTTTT-
AAGTTTTATTTTATTTTTTACTTCTTTATAATGAACCTCAAA
>TSH-R30055
TTAAGAGTGCACTTAATTGTGGCTTGAAATTTTACTTTATTTACACCCAACGTC-
TTCGGGACACTGCGGC-
AATTTATTGCTTAGCGAATTTAACCCCTGCGGTTAGGCATATATAA-TTCTCTCT-G-
AGGGTTGTATGTGTTCTAATCTTTGTTTTTTTTATTTT-
CAACCACAAATTTATACATATGTATATATGTATT-ATTTACTATCAAGTAAATAAATAT-
AAAACTTTTAACAATGGATCTCTTGGCTCTCA-
CATCGATGAAGAACCCAGTGAAATGTGATAAGTAATGTGAATTGCAGAATTCAGGGAATCAT
CGAATTTTTGAACGCATATTGGCCCTTTTGGCTATTCCAAAAGGTACCCCTGTTTGAGAGTA
TGAAAGGTCAGGGTGTTGAGAGAGTTTTTAAAAAAAA-
GAAAAAGGCAAAGTAACACTTTAAGTGTTATTTTGTCTTTGATTTTTTTTTT-
CTCTTAGCATCTTGGATATTGGGTG-CTTGCCATTATTAAGTTTGATGG-
CTCACCTTAAATTTATAAGTTGTTTTTT-
ATTAAGGATGAAAAGTCTTTTGATGGCTTGATGTTATTGA-
TATATGAAATGTCATTCATCAAGAAATCAGGGGGGTGACTAACCTTGATGAGAAATGTTGAC
TTTATAAACACATGTTAAAAGATAAATAAAAAGTAAAAGATAAAAAAAATAAAAAATAAAAAA
TTAAAGGGAAAGAAGTAAAAAGAATGAGAGGTATGACATATTTTGAAAATATGTCCCTTG-
TTCTCTTTTTATTTTGATTATCTTTATTTTTTTAT-TTTTAATTTTAATTTTTATGTTTT-
AAGTTTTATTTTATTTTTTACTTCTTTATAATGAATCTCAAA
>TSH-R30059
TTAAGAGTGCACTTAATTGTGGCTTGAAATTTTACTTTATTTACCCCCAACTTC-
TTCGGGACATTGCGGC-
AATTTATTGCTTAGCGAATTTAACCCCTGCGGTTAGGCATATATAA-TTCTCTCT--
GAGGGTTGAATG-TGTTCAATTCTTGTTTTTTTTATTTT-
CAACCACAAATTTATACATATGTATATATGTATT-ATTTACTATCAAGTAAATAAATAT-
AAAACTTTTAACAATGGATCTCTTGGCTCTCA-
CATCGATGAAGAACACAGTGAAATGTGATAAGTAATGTGAATTGCAGAATTCAGTGAATCAT
CGAATTTTTGAACGCATATTGCGCCTTTTGGCTATTCCAAAAGGTACACCTGTTTGAGAGTA
TGAAAGGTCAGGGTGTTGAGAGAGTTATTAAAAAAAA-
GAAAAAGGCAAAGTAACACTTTAAGTGTTATTTTGTCTTTGATTTTTTTTTT-
CTCTTAGCATCTTGGATATTGGGTG-CTTGCCATTATTAAGTTTGATGG-
CTCACCTTAAATTTATAAGTTGTTTTTT-
ATTAAGGATGAAAAGTCTTTTGATGGCTTGATGTTATTGA-
TATATGAAATGTCATTCATCAAGAAATCAGGGGGGTGACTAACCTTGATGAGAAATGTTGAC
TTTATAAACACATGTTAAAAGATAAATAAAAAGTAAAAGATAAAAAAAATAAAAAATTAAAAA

TTAAAGGGAAAGAAGTAAAAAGAATGAGAGGTATGACATATTTTGAAAATATGTCCCTTG-
TTCTCTTTTTATTTTGATTATCTTTATTTTTTTAT-TTTTAATTTTAATTTTTATGTTTT-
AAGTTTTATTTTATTTTTTACTTCTTTATAATGAATCTCAAA
>TSH-R30063
TTAAGAGTGCACTTAATTGTGGCTTGAAATTTTACTTTATTTACCCCCAACGTC-
TTCGGGACACGGCGGC-
AATTTATTGCTTAGCGAATTTAACCCCTGCGGTTAGGCATATATAA-TTCTCTCT--
GAGGGTTGTATGTGTTCTAATCTT-GTTTTTTTTATTTT-
CAACCACAAATTTATACATATGTATATATGTATT-ATTTACTATCAAGTAAATAAATAT-
AAAACTTTTAACAATGGATCTCTTGGCTCTCA-
CATCGATGAAGAACACAGTGAAATGTGATAAGTAATGGGAATTGCAGAATTCAGTGAATCAT
CGAATTTTTGAACGCATATTGCGCCTTTTGGCTATTCCAAAAGGTACACCTGTTTGAGAGTA
TGAAAGGTCAGGGTGTTGAGAGAGTTATTAAAAAAAA-
GAAAAAGGCAAAGTAACACTTTAAGTGTTATTTTGTCTTTGATTTTTTTTTT-
CTCTTAGCATCTTGGATATTGGGTG-CTTGCCATTATTAAGTTTGATGG-
CTCACCTTAAATTTATAAGTTGTTTTTT-
ATTAAGGATGAAAAGTCTTTTGATGGCTTGATGTTATTGA-
TATATGAAATGTCATTCATCAAGAAATCAGGGGGGTGACTAACCTTGATGAGAAATGTTGAC
TTTATAAACACATGTTAAAAGATAAATAAAAAGTAAAAGATAAAAAAAATAAAAAATTAAAAA
TTAAAGGGAAAGAAGTAAAAAGAATGAGAGGTATGACATATTTTGAAAATATGTCCCCTG-
TTCTCTTTTTATTTTGATTATCTTTATTTTTTTAT-TTTTAATTTTAATTTTTATGGTTT-
AAGTTTTATTTTATTTTTTACTTCTTTATAATGAATCTCAAA
>TSH-R30067
TTAAGAGTGCACTTAATTGTGGCTTGAAATTTTACTTAATTTCCCCCCACCGCC-
TTCGGAACCCGGCGGC-ATTTAATGGTTAACCGAATTTAACCCCGGCGGTAAGGCAAA-
ATAA-TTCTCTCC--GAAGGGTGGAAGGGGTCCAATCCTTGTTTTTTTTATTTT-
CAAACACAAATTTATACATATGTATATATGTATT-ATTTACTATCAAGTAAATAAATAT-
AAAACTTTTAACAATGGATCTCTTGGCTCTCA-
CATCGATGAAGAACACAGTGAAATGTGATAAGTAATGTGAATTGCAGAATTCAGTGAATCAT
CGAATTTTTGAACGCATATTGCGCCTTTTGGCTATTCCAAAAGGTACCCCTGTTTGAGAGTA
TGAAAGGTCAGGGTGTTGAGAGAGTTATTAAAAAAAA-
GAAAAAGGCAAAGTAACACTTTAAGTGTTATTTTGTCTTTGATTTTTTTTTT-
CTCTTAGCATCTTGGATATTGGGTGGCTTGCCATTATTAAGTTTGATGG-
CTCACCTTAAATTTATAAGTTGTTTTTT-
ATTAAGGATGAAAAGTCTTTTGATGGCTTGATGTTATTGA-
TATATGAAATGTCATTCATCAAGAAATCAGGGGGGTGACTAACCTTGATGAGAAATGTTGAC
TTTATAAACACATGTTAAAAGATAAATAAAAAGTAAAAGATAAAAAAAATAAAAAATTAAAAA
TTAAAGGGAAAGAAGTAAAAAGAATGAGAGGTATGACATATTTTGAAAATATGTCCCTTG-
TTCTCTTTTTATTTTGATTATCTTTATTTTTTTAT-TTTTAATTTTAATTTTTATGTTTT-

AAGTTTTATTTTATTTTTTACTTCTTTATAATGAATCTCAAA
>TSH-R30069
TTAAGAGTGCACTTAATTGTGGCTTGAAATTTTACTTTATTTACACCCAACGTC-
TTCGGGACACTGCGGC-
AATTTATTGCTTAGCGAATTTAACCCCTGCGGTTAGGCATATATAA-TTCTCTCT--
GAGGGTTGTATGTGTTCTAATCTT-GTTTTTTTTATTTT-
CAACCACAAATTTATACATATGTATATATGTATT-ATTTACTATCAAGTAAATAAATAT-
AAAACTTTTAACAATGGATCTCTTGGCTCTCA-
CATCGATGAAGAACACAGTGAAATGTGATAAGTAATGTGAATTGCAGAATTCAGTGAATCAT
CGAATTTTTGAACGCATATTGCGCCTTTTGGCTATTCCAAAAGGTACACCTGTTTGAGAGTA
TGAAAGGTCAGGGTGTTGAGAGAGTTATTAAAAAAAA-
GAAAAAGGCAAAGTAACACTTTAAGTGTTATTTTGTCTTTGATTTTTTTTTT-
CTCTTAGCATCTTGGATATTGGGTG-CTTGCCATTATTAAGTTTGATGG-
CTCACCTTAAATTTATAAGTTGTTTTTTT-
TTAAGGATGAAAAGTCTTTTGATGGCTTGATGTTATTGA-
TATATGAAATGTCATTCATCAAGAAATCAGGGGGGTGACTAACCTTGATGAGAAATGGTGA
CTTTATAAACACATGTTAAAAGATAAATAAAAAGTAAAAGATAAAAAAAATAAAAAATAAAAA
ATTAAAGGGAAAGAAGTAAAAAGAATGAGAGGTATGACATATTTTGAAAATATGTCCCTTG-
TTCTCTTTTTATTTTGATTATCTTTATTTTTTTAT-TTTTAATTTTAATTTTTATGTTTT-
AAGTTTTATTTTATTTTTTACTTCTTTATAATGAATCTCAAA
>TSH-R30071
TTAAGAGTGCACTTAATTGTGGCTTGAAATTTTACTTAATTTCCCCCCAACGTC-
TTCGGAACACTGCGGC-
AATTTATTGCTTAGCGAATTTAACCCCTGCGGTTAGGCATATATAA-
TTCTCTCTTGAGGGGTGGTATGTGTTCTAATCCTTGTTTTTTTTATTTT-
CAACCCCAAATTTATACATATGTATATATGTTTTTATTTACTATCAAGTAAATAAATATTAAA
ACTTTTAACAATGGATCTCTTGGCTCTCA-
CATCGATGAAAAACCCAGTGAAATGGGAAAAGTAATGGGAATTGCAAAATTCAGGGAATCAT
CGAATTTTTGAACCCATATTGGCCCTTTTGGCTTTTCCAAAAGGTACCCCTGTTTGAAAATA
TGAAAGGTCAGGGGGTTGAAGAAGTTTTTAAAAAAAA-
GAAAAAGGCAAAGTAACCCTTTAAGTGTTATTTTGTCTTTGATTTTTTTTTTTCTCTTAGCA
TCTTGGATATTGGGTG-CTTGCCATTATTAAGTTTGATGG-
CTCACCTTAAATTTATAAGTTGTTTTTT-
ATTAAGGATGAAAAGTCTTTTGATGGCTTGATGTTATTGA-
TATATGAAATGTCATTCATCAAGAAATCAGGGGGGTGACTAACCTTGATGAGAAATGTTGAC
TTTATAAACACATGTTAAAAGATAAATAAAAAGTAAAAGATAAAAAAAATAAAAAATTAAAAA
TTAAAGGGAAAGAAGTAAAAAGAATGAGAGGTATGACATATTTTGAAAATATGTCCCTTG-
TTCTCTTTTTATTTTGATTATCTTTATTTTTTTAT-TTTTAATTTTAATTTTTATGTTTT-
AAGTTTTATTTTATTTTTTACTTCTTTATAATGAATCTCAAA

>TSH-R30073
TTAAGAGTGCACTTAATTGTGGCTTGAAATTTTACTTTATTTACACCCAACGTC-
TTCGGGACACTGCGGC-
AATTTATTGCTTAGCGAATTTAACCCCTGCGGTTAGGCATATATAA-TTCTCTCT--
GAGGGTTGTATGTGTTCTAAT-CTTGTTTTTTTTATTTT-
CAACCACAAATTTATACATATGTATATATGTATT-ATTTACTATCAAGTAAATAAATAT-
AAAACTTTTAACAATGGATCTCTTGGCTCTCA-
CATCGATGAAGAACACAGTGAAATGTGATAAGTAATGTGAATTGCAGAATTCAGTGAATCAT
CGAATTTTTGAACGCATATTGCGCCTTTTGGCTATTCCAAAAGGTACCCCTGTTTGAGAGTA
TGAAAGGTCAGGGTGTTGAGAGAGTTATTAAAAAAAA-
GAAAAAGGCAAAGTAACACTTTAAGTGTTATTTTGTCTTTGA-
TTTTTTTTTTCTCTTAGCATCTTGGATATTGGGTG-CTTGCCATTATTAAGTTTGATGG-
CTCACCTTAAATTTATAAGTTGTTTTTT-
ATTAAGGATGAAAAGTCTTTTGATGGCTTGATGTTATTGA-
TATATGAAATGTCATTCATCAAGAAATCAGGGGGGTGACTAACCTTGATGAGAAATGTTGAC
TTTATAAACACATGTTAAAAGATAAATAAAAAGTAAAAGATAAAAAAAATAAAAAATAAAAAA
TTAAAGGGAAAGAAGTAAAAAGAATGAGAGGTATGACATATTTTGAAAATATGTCCCTTG-
TTCTCTTTTTATTTTGATTATCTTTATTTTTTTAT-TTTTAATTTTAATTTTTATGTTTT-
AAGTTTTATTTTATTTTTTACTTCTTTATAATGAATCTCAAA
>TSH-R30080
TTAAGAGTGCACTTAATTGTGGCTTGAAATTTTACTTAATTTACACCCAACGTC-
TTCGGGACACTGCGGCCAATTTATGGCTTAGCGAATTTAACCCCGGCGGTTAGGCATATAT
AA-TTCTCTCT--GAGGGTGGTAGGGGTTCTAATCTT-GTTTTTTTTATTTT-
CAACCACAAATTTATACATATGTATATATGTATT-ATTTACTATCAAGTAAATAAATAT-
AAAACTTTTAACAATGGATCTCTTGGCTCTCA-
CATCGATGAAGAACACAGTGAAATGTGATAAGTAATGTGAATTGCAGAATTCAGTGAATCAT
CGAATTTTTGAACGCATATTGCGCCTTTTGGCTATTCCAAAAGGTACCCCTGTTTGAGAGTA
TGAAAGGTCAGGGTGTTGAGAGAGTTATTAAAAAAAA-
GAAAAAGGCAAAGTAACACTTTAAGTGTTATTTTGTCTTTGATTTTTTTTTT-
CTCTTAGCATCTTGGATATTGGGTG-CTTGCCATTATTAAGTTTGATGG-
CTCACCTTAAATTTATAAGTTGTTTTTT-
ATTAAGGATGAAAAGTCTTTTGATGGCTTGATGTTATTGA-
TATATGAAATGTCATTCATCAAGAAATCAGGGGGGTGACTAACCTTGATGAGAAATGTTGAC
TTTATAAACACATGTTAAAAGATAAATAAAAAGTAAAAGATAAAAAAAATAAAAAATTAAAAA
TTAAAGGGAAAGAAGTAAAAAGAATGAGAGGTATGACATATTTTGAAAATATGTCCCTTG-
TTCTCTTTTTATTTTGATTATCTTTATTTTTTTAT-TTTTAATTTTAATTTTTATGTTTT-
AAGTTTTATTTTATTTTTTACTTCTTTATAATGAATCTCAAA
>TSH-R30085
TTAAGAGTGCACTTAATTGTGGCTTGAAATTTTACTTTATTTACACCCAACGCC-

TTCGGAACACTGCGGC-
AATTTATGGCTTACCGAATTTAACCCCGGCGGTTAGGCATATATAA-TTCTCTCT--
GAGGGTGGAAGGTGTCCAAATCTT-GTTTTTTTTATTTT-
CAACCACAAATTTATACATATGTATATATGTATT-ATTTACTATCAAGTAAATAAATAT-
AAAACTTTTAACAATGGATCTCTTGGCTCTCA-
CATCGATGAAGAACACAGTGAAATGTGATAAGTAATGTGAATTGCAGAATTCAGTGAATCAT
CGAATTTTTGAACGCATATTGCGCCTTTTGGCTATTCCAAAAGGTACACCTGTTTGAGAGTA
TGAAAGGTCAGGGTGTTGAGAGAGTTATTAAAAAAAA-
GAAAAAGGCAAAGTAACACTTTAAGTGTTATTTTGTCTTTGATTTTTTTTTT-
CTCTTAGCATCTTGGATATTGGGTG-CTTGCCATTATTAAGTTTGATGG-
CTCACCTTAAATTTATAAGTTGTTTTTT-
ATTAAGGATGAAAAGTCTTTTGATGGCTTGATGTTATTGA-
TATATGAAATGTCATTCATCAAGAAATCAGGGGGGTGACTAACCTTGATGAGAAATGTTGAC
TTTATAAACACATGTTAAAAGATAAATAAAAAGTAAAAGATAAAAAAAATAAAAAATTAAAAA
TTAAAGGGAAAGAAGTAAAAAGAATGAGAGGTATGACATATTTTGAAAATATGTCCCTTG-
TTCTCTTTTTATTTTGATTATCTTTATTTTTTTAT-TTTTAATTTTAATTTTTATGTTTT-
AAGTTTTATTTTATTTTTTACTTCTTTATAATGAATCTCAAA
>TSH-R30088
TTAAGAGTGCACTTAATTGTGGCTTGAAATTTTACTTTATTTCCACCCAACGTC-
TTCGGAACACTGCGGC-
AATTAATGGCTTAGCGAATTTAACCCCGGCGGTTAGGCATATATAA-TTCTCTCT--
GAGGGTGGTAGGGGTTCTAATCTT-GTTTTTTTTATTTT-
CAACCACAAATTTATACATATGTATATATGTATT-ATTTACTATCAAGTAAATAAATAT-
AAAACTTTTAACAATGGATCTCTTGGCTCTCA-
CATCGATGAAGAACACAGTGAAATGTGATAAGTAATGTGAATTGCAGAATTCAGTGAATCAT
CGAATTTTTGAACGCATATTGCGCCTTTTGGCTATTCCAAAAGGTACACCTGTTTGAGAGTA
TGAAAGGTCAGGGTGTTGAGAGAGTTATTAAAAAAAA-
GAAAAAGGCAAAGTAACACTTTAAGTGTTATTTTGTCTTTGATTTTTTTTTT-
CTCTTAGCATCTTGGATATTGGGTG-CTTGCCATTATTAAGTTTGATGG-
CTCACCTTAAATTTATAAGTTGTTTTTT-
ATTAAGGATGAAAAGTCTTTTGATGGCTTGATGTTATTGA-
TATATGAAATGTCATTCATCAAGAAATCAGGGGGGTGACTAACCTTGATGAGAAATGTTGAC
TTTATAAACACATGTTAAAAGATAAATAAAAAGTAAAAGATAAAAAAAATAAAAAATTAAAAA
TTAAAGGGAAAGAAGTAAAAAGAATGAGAGGTATGACATATTTTGAAAATATGTCCCTTG-
TTCTCTTTTTATTTTGATTATCTTTATTTTTTTAT-TTTTAATTTTAATTTTTATGTTTT-
AAGTTTTATTTTATTTTTTACTTCTTTATAATGAATCTCAAA
>TSH-R30098
TTAAGAGTGCACTTAATTGTGGCTTGAAATTTTACTTTTTTTACACCCAACGTC-
TTCGGGACACTGCGGC-

AATTTATTGCTTACCGAATTTAACCCCTGCGGTTAGGCATATATAT-TTCTCTCT--
GAGGGTTGTATGTGTTCTAAATCTTGTTTTTTTTATTTT-
CAACCACAAATTTATACATATGTATATATGTATT-ATTTACTATCAAGTAAATAAATAT-
AAAACTTTTAACAATGGATCTCTTGGCTCTCA-
CATCGATGAAGAACACAGTGAAATGTGATAAGTAATGTGAATTGCAGAATTCAGTGAATCAT
CGAATTTTTGAACGCATATTGCGCCTTTTGGCTATTCCAAAAGGTACACCTGTTTGAGAGTA
TGAAAGGTCAGGGTGTTGAGAGAGTTATTAAAAAAAA-
GAAAAAGGCAAAGTAACACTTTAAGTGTTATTTTGTCTTTGATTTTTTTTTT-
CTCTTAGCATCTTGGATATTGGGTG-CTTGCCATTATTAAGTTTGATGG-
CTCACCTTAAATTTATAAGTTGTTTTTT-
ATTAAGGATGAAAAGTCTTTTGATGGCTTGATGTTATTGA-
TATATGAAATGTCATTCATCAAGAAATCAGGGGGGTGACTAACCTTGATGAGAAATGTTGAC
TTTATAAACACATGTTAAAAGATAAATAAAAAGTAAAAGATAAAAAAAATAAAAAATAAAAAA
TTAAAGGGAAAGAAGTAAAAAGAATGAGAGGTATGACATATTTTGAAAATATGTCCCTTG-
TTCTCTTTTTATTTTGATTATCTTTATTTTTTTAT-TTTTAATTTTAATTTTTATGTTTT-
AAGTTTTATTTTATTTTTTACTTCTTTATAATGAATCTCAAA
>TSH-R30101
TTAAGAGTGCACTTAATTGTGGCTTGAAATTTTACTTAATTTACCCCCAACGCC-
TTCGGGACACTGCGGC-
AATTTATGGCTTAGCGAATTTAACCCCTGCGGTTAGGCATATATAA-TTCTCTCT--
GAGGGTGGAAGG-GTTCCAAACCTTGTTTTTTTTATTTT-
CAACCACAAATTTATACATATGTATATATGTATT-ATTTACTATCAAGTAAATAAATAT-
AAAACTTTTAACAATGGATCTCTTGGCTCTCA-
CATCGATGAAGAACACAGTGAAATGTGATAAGTAATGTGAATTGCAGAATTCAGTGAATCAT
CGAATTTTTGAACGCATATTGCGCCTTTTGGCTATTCCAAAAGGTACACCTGTTTGAGAGTA
TGAAAGGTCAGGGTGTTGAGAGAGTTATTAAAAAAAA-
GAAAAAGGCAAAGTAACACTTTAAGTGTTATTTTGTCTTTGATTTTTTTTTT-
CTCTTAGCATCTTGGATATTGGGTG-CTTGCCATTATTAAGTTTGATGG-
CTCACCTTAAATTTATAAGTTGTTTTTT-
ATTAAGGATGAAAAGTCTTTTGATGGCTTGATGTTATTGA-
TATATGAAATGTCATTCATCAAGAAATCAGGGGGGTGACTAACCTTGATGAGAAATGTTGAC
TTTATAAACACATGTTAAAAGATAAATAAAAAGTAAAAGATAAAAAAAATAAAAAATTAAAAA
TTAAAGGGAAAGAAGTAAAAAGAATGAGAGGTATGACATATTTTGAAAATATGTCCCTTG-
TTCTCTTTTTATTTTGATTATCTTTATTTTTTTAT-TTTTAATTTTAATTTTTATGTTTT-
AAGTTTTATTTTATTTTTTACTTCTTTATAATGATCCTCAAA
>TSH-R30104
TTAAGAGTGCACTTAATTGTGGCTTGAAATTTTACTTTATTTACACCCAACGTC-
TTCGGGACACTGCGGC-
AATTTATTGCTTAGCGAATTTAACCCCTGCGGTTAGGCATATATAA-TTCTCTCT--

GAGGGTGGAAGGGGTTCTAATCTT-GTTTTTTTTATTTT-
CAACCACAAATTTATACATATGTATATATGTATT-ATTTACTATCAAGTAAATAAATAT-
AAAACTTTTAACAATGGATCTCTTGGCTCTCA-
CATCGATGAAGAACACAGTGAAATGTGATAAGTAATGTGAATTGCAGAATTCAGTGAATCAT
CGAATTTTTGAACGCATATTGCGCCTTTTGGCTATTCCAAAAGGTACCCCTGTTTGAGAGTA
TGAAAGGTCAGGGTGTTGAGAGAGTTATTAAAAAAAA-
GAAAAAGGCAAAGTAACACTTTAAGTGTTATTTTGTCTTTGATTTTTTTTTT-
CTCTTAGCATCTTGGATATTGGGTG-CTTGCCATTATTAAGTTTGATGG-
CTCACCTTAAATTTATAAGTTGTTTTTT-
ATTAAGGATGAAAAGTCTTTTGATGGCTTGATGTTATTGA-
TATATGAAATGTCATTCATCAAGAAATCAGGGGGGTGACTAACCTTGATGAGAAATGTTGAC
TTTATAAACACATGTTAAAAGATAAATAAAAAGTAAAAGATAAAAAAAATAAAAAATTAAAAA
TTAAAGGGAAAGAAGTAAAAAGAATGAGAGGTATGACATATTTTGAAAATATGTCCCTTG-
TTCTCTTTTTATTTTGATTATCTTTATTTTTTTAT-TTTTAATTTTAATTTTTATGTTTT-
AAGTTTTATTTTATTTTTTACTTCTTTATAATGAATCTCAAA
>TSH-R30107
TTAAGAGTGCACTTAATTGTGGCTTGAAATTTTACTTTATTTACACCCAACGTC-
TTCGGGACACTGCGGC-
AATTTATTGCTTAGCGAATTTAACCCCTGCGGTTAGGCATATATAA-TTCTCTCT--
GAGGGTTGTATGTGTTCTAATCTT-GTTTTTTTTATTTT-
CAACCACAAATTTATACATATGTATATATGTATT-ATTTACTATCAAGTAAATAAATAT-
AAAACTTTTAACAATGGATCTCTTGGCTCTCA-
CATCGATGAAGAACACAGTGAAATGTGATAAGTAATGTGAATTGCAGAATTCAGTGAATCAT
CGAATTTTTGAACGCATATTGCGCCTTTTGGCTATTCCAAAAGGTACACCTGTTTGAGAGTA
TGAAAGGTCAGGGTGTTGAGAGAGTTATTAAAAAAAA-
GAAAAAGGCAAAGTAACACTTTAAGTGTTATTTTGTCTTTGATTTTTTTTTT-
CTCTTAGCATCTTGGATATTGGGTG-CTTGCCATTATTAAGTTTGATGG-
CTCACCTTAAATTTATAAGTTGTTTTTT-
ATTAAGGATGAAAAGTCTTTTGATGGCTTGATGTTATTGA-
TATATGAAATGTCATTCATCAAGAAATCAGGGGGGTGACTAACCTTGATGAGAAATGTTGAC
TTTATAAACACATGTTAAAAGATAAATAAAAAGTAAAAGATAAAAAAAATAAAAAATTAAAAA
TTAAAGGGAAAGAAGTAAAAAGAATGAGAGGTATGACATATTTTGAAAATATGTCCCTTG-
TTCTCTTTTTATTTTGATTATCTTTATTTTTTTAT-TTTTAATTTTAATTTTTATGTTTT-
AAGTTTTATTTTATTTTTTACTTCTTTATAATGAATCTCAAA
>TSH-R30109
TTAAGAGTGCACTTAATTGTGGCTTGAAATTTTACTTTATTTACCCCCAACGTC-
TTCGGAACACTGCGGC-
AATTTATTGCTTACCGAATTTAACCCCTGCGGTTAGGCATATATAA-TTCTCTCT--
GAGGGTTGTATGTGTTC-AATCCTTGTTTTTTTTATTTT-

CAACCCCAAATTTATACATATGTATATATGTATT-ATTTACTATCAAGTAAATAAATAT-
AAAACTTTTAACAATGGATCTCTTGGCTCTCA-
CATCGATGAAGAACACAGTGAAATGTGATAAGTAATGGGAATTGCAGAATTCAGTGAATCAT
CGAATTTTTGAACGCATATTGCGCCTTTTGGCTATTCCAAAAGGTACACCTGTTTGAGAGTA
TGAAAGGTCAGGGTGTTGAGAGAGTTATTAAAAAAAA-
GAAAAAGGCAAAGTAACCCTTTAAGGGTTATTTTGTCTTTGATTTTTTTTTT-
CTCTTAGCATCTTGGATATTGGGTG-CTTGCCATTATTAAGTTTGATGG-
CTCACCTTAAATTTATAAGTTGTTTTTT-
ATTAAGGATGAAAAGTCTTTTGATGGCTTGATGTTATGGA-
TATATGAAATGTCATTCATCAAGAAATCAGGGGGGTGACTAACCTTGATGAGAAATGTTGAC
TTTATAAACACATGTTAAAAGATAAATAAAAAGTAAAAGATAAAAAAAATAAAAAATTAAAAA
TTAAAGGGAAAGAAGTAAAAAGAATGAGAGGTATGACATATTTTGAAAATATGTCCCTTG-
TTCTCTTTTTATTTTGATTATCTTTATTTTTTTAT-TTTTAATTTTAATTTTTATGTTTT-
AAGTTTTATTTTATTTTTTACTTCTTTATAATGAATCTCAAA
>TSH-R30118
TTAAGAGTGCACTTAATTGTGGCTTGAAATTTTACTTAATTTACCCCCAACGCC-
TTCGGGACACGGCGGC-
ATTTAATGGCTTAGCGAATTTAACCCCGGCGGTAAGGCATATATAA-TTCCCTCG--
GAGGGTGGTATGGGTTCTAACCTT-GTTTTTTTTATTTT-
CACCCACAAATTTATACATATGTATATATGTATT-ATTTACTATCAAGTAAATAAATAT-
AAAACTTTTAACAATGGATCTCTTGGCTCTCA-
CATCGATGAAGAACACAGTGAAATGTGATAAGTAATGTGAATTGCAGAATTCAGTGAATCAT
CGAATTTTTGAACGCATATTGCGCCTTTTGGCTATTCCAAAAGGTACACCTGTTTGAGAGTA
TGAAAGGTCAGGGTGTTGAGAGAGTTATTAAAAAAAA-
GAAAAAGGCAAAGTAACACTTTAAGTGTTATTTTGTCTTTGATTTTTTTTTT-
CTCTTAGCATCTTGGATATTGGGTG-CTTGCCATTATTAAGTTTGATGG-
CTCACCTTAAATTTATAAGTTGTTTTTT-
ATTAAGGATGAAAAGTCTTTTGATGGCTTGATGTTATTGA-
TATATGAAATGTCATTCATCAAGAAATCAGGGGGGTGACTAACCTTGATGAGAAATGTTGAC
TTTATAAACACATGTTAAAAGATAAATAAAAAGTAAAAGATAAAAAAAATAAAAAATTAAAAA
TTAAAGGGAAAGAAGTAAAAAGAATGAGAGGTATGACATATTTTGAAAATATGTCCCTTG-
TTCTCTTTTTATTTTGATTATCTTTATTTTTTTAT-TTTTAATTTTAATTTTTATGTTTT-
AAGTTTTATTTTATTTTTTACTTCTTTATAATGAATCTCAAA
>TSH-R30120
TTAAGAGTGCACTTAATTGTGGCTTGAAATTTTACTTTATTTACACCCAACGTC-
TTCGGGACACTGCGGC-
AATTTATTGCTTAGCGAATTTAACCCCTGCGGTTAGGCATATATAA-TTCTCTCT--
GAGGGTTGTATGTGTTCTAAT-
CTTGTTTTTTTTATTTTTCAACCACAAATTTATACATATGTATATATGTATT-

ATTTACTATCAAGTAAATAAATAT-AAAACTTTTAACAATGGATCTCTTGGCTCTCA-
CATCGATGAAGAACACAGTGAAATGTGATAAGTAATGTGAATTGCAGAATTCAGTGAATCAT
CGAATTTTTGAACGCATATTGCGCCTTTTGGCTATTCCAAAAGGTACCCCTGTTTGAGAGTA
TGAAAGGTCAGGGTGTTGAGAGAGTTATTAAAAAAAA-
GAAAAAGGCAAAGTAACACTTTAAGTGTTATTTTGTCTTTGATTTTTTTTTT-
CTCTTAGCATCTTGGATATTGGGTG-CTTGCCATTATTAAGTTTGATGG-
CTCACCTTAAATTTATAAGTTGTTTTTT-
ATTAAGGATGAAAAGTCTTTTGATGGCTTGATGTTATTGA-
TATATGAAATGTCATTCATCAAGAAATCAGGGGGGTGACTAACCTTGATGAGAAATGTTGAC
TTTATAAACACATGTTAAAAGATAAATAAAAAGTAAAAGATAAAAAAAATAAAAAATTAAAAA
TTAAAGGGAAAGAAGTAAAAAGAATGAGAGGTATGACATATTTTGAAAATATGTCCCTTG-
TTCTCTTTTTATTTTGATTATCTTTATTTTTTTAT-TTTTAATTTTAATTTTTATGTTTT-
AAGTTTTATTTTATTTTTTACTTCTTTATAATGAATCTCAAA
>TSH-R30125
TTAAGAGTGCACTTAATTGTGGCTTGAAATTTTACTTTATTTACACCCAACGTC-
TTCGGGACACTGCGGC-
AATTTATTGCTTAGCGAATTTAACCCCTGCGGTTAGGCATATATAA-TTCTCTCT--
GAGGGTTGTATGTGTTCTAATCTT-GTTTTTTTTATTTT-
CAACCACAAATTTATACATATGTATATATGTATT-ATTTACTATCAAGTAAATAAATAT-
AAAACTTTTAACAATGGATCTCTTGGCTCTCA-
CATCGATGAAGAACCCAGTGAAATGTGATAAGTAATGTGAATTGCAGAATTCAGTGAATCAT
CGAATTTTTGAACGCATATTGCGCCTTTTGGCTATTCCAAAAGGTACACCTGTTTGAGAGTA
TGAAAGGTCAGGGTGTTGAGAGAGTTATTAAAAAAAA-
GAAAAAGGCAAAGTAACACTTTAAGTGTTATTTTGTCTTTGATTTTTTTTTT-
CTCTTAGCATCTTGGATATTGGGTG-CTTGCCATTATTAAGTTTGATGG-
CTCACCTTAAATTTATAAGTTGTTTTTT-
ATTAAGGATGAAAAGTCTTTTGATGGCTTGATGTTATTGA-
TATATGAAATGTCATTCATCAAGAAATCAGGGGGGTGACTAACCTTGATGAGAAATGTTGAC
TTTATAAACCCATGTTAAAAGATAAATAAAAAGTAAAAGATAAAAAAAATAAAAAATAAAAAA
TTAAAGGGAAAGAAGTAAAAAGAATGAGAGGTATGACATATTTTGAAAATATGTCCCTTG-
TTCTCTTTTTATTTTGATTATCTTTATTTTTTTAT-TTTTAATTTTAATTTTTATGTTTT-
AAGTTTTATTTTATTTTTTACTTCTTTATAATGAATCTCAAA
>TSH-R30129
TTAAGAGTGCACTTAATTGTGGCTTGAAATTTTACTTTATTTACACCAAACGTC-
TTCGGGACACTGCGGC-
AATTTATTGCTTAGCGAATTTAACCCCTGCGGTTAGGCATATATAA-TTCTCTCT--
GAGGGTTGTATGTGTTCTAATCTT-GTTTTTTTTATTTT-
CAACCACAAATTTATACATATGTATATATGTATT-ATTTACTATCAAGTAAATAAATAT-
AAAACTTTTAACAATGGATCTCTTGGCTCTCA-

CATCGATGAAGAACACAGTGAAATGTGATAAGTAATGTGAATTGCAGAATTCAGTGAATCAT
CGAATTTTTGAACGCATATTGCGCCTTTTGGCTATTCCAAAAGGTACACCTGTTTGAGAGTA
TGAAAGGTCAGGGTGTTGAGAGAGTTATTAAAAAAAA-
GAAAAAGGCAAAGTAACACTTTAAGTGTTATTTTGTCTTTGATTTTTTTTTT-
CTCTTAGCATCTTGGATATTGGGTG-CTTGCCATTATTAAGTTTGATGG-
CTCACCTTAAATTTATAAGTTGTTTTTT-
ATTAAGGATGAAAAGTCTTTTGATGGCTTGATGTTATTGA-
TATATGAAATGTCATTCATCAAGAAATCAGGGGGGTGACTAACCTTGATGAGAAATGTTGAC
TTTATAAACACATGTTAAAAGATAAATAAAAAGTAAAAGATAAAAAAAATAAAAAATAAAAAA
TTAAAGGGAAAGAAGTAAAAAGAATGAGAGGTATGACATATTTTGAAAATATGTCCCTTG-
TTCTCTTTTTATTTTGATTATCTTTATTTTTTTAT-TTTTAATTTTAATTTTTATGTTTT-
AAGTTTTATTTTATTTTTTACTTCTTTATAATGAATCTCAAA
>TSH-R30133
TTAAGAGTGCACTTAATTGTGGCTTGAAATTTTACTTTATTTACACCCAACGTC-
TTCGGGACACTGCGGC-
AATTTATTGCTTAGCGAATTTAACCCCTGCGGTTAGGCATATATAA-TTCTCTCT--
GAGGGTTGTATGTGTTCTAAT-CTTGTTTTTTTTATTTT-
CAACCACAAATTTATACATATGTATATATGTATT-ATTTACTATCAAGTAAATAAATAT-
AAAACTTTTAACAATGGATCTCTTGGCTCTCA-
CATCGATGAAGAACACAGTGAAATGTGATAAGTAATGTGAATTGCAGAATTCAGTGAATCAT
CGAATTTTTGAACGCATATTGCGCCTTTTGGCTATTCCAAAAGGTACCCCTGTTTGAGAGTA
TGAAAGGTCAGGGTGTTGAGAGAGTTATTAAAAAAAA-
GAAAAAGGCAAAGTAACCCTTTAAGTGTTATTTTGTCTTTGA-
TTTTTTTTTTCTCTTAGCATCTTGGATATTGGGTG-CTTGCCATTATTAAGTTTGATGG-
CTCACCTTAAATTTATAAGTTGTTTTTT-
ATTAAGGATGAAAAGTCTTTTGATGGCTTGATGTTATTGA-
TATATGAAATGTCATTCATCAAGAAATCAGGGGGGTGACTAACCTTGATGAGAAATGTTGAC
TTTATAAACACATGTTAAAAGATAAATAAAAAGTAAAAGATAAAAAAAATAAAAAATAAAAAA
TTAAAGGGAAAGAAGTAAAAAGAATGAGAGGTATGACATATTTTGAAAATATGTCCCTTG-
TTCTCTTTTTATTTTGATTATCTTTATTTTTTTAT-TTTTAATTTTAATTTTTATGTTTT-
AAGTTTTATTTTATTTTTTACTTCTTTATAATGAATCTCAAA
>TSH-R30137
TTAAGAGTGCACTTAATTGTGGCTTGAAATTTTACTTTATTTACCCCCAACGTC-
TTCGGGACACTGCGGC-
AATTTATTGCTTAGCGAATTTAACCCCTGCGGTTAGGCATATATAA-TTCTCTCT--
GAGGGTTGTATGTGTTCTAATCTT-GTTTTTTTTATTTT-
CAACCACAAATTTATACATATGTATATATGTATT-ATTTACTATCAAGTAAATAAATAT-
AAAACTTTTAACAATGGATCTCTTGGCTCTCA-
CATCGATGAAGAACACAGTGAAATGTGATAAGTAATGTGAATTGCAGAATTCAGTGAATCAT

CGAATTTTTGAACGCATATTGCGCCTTTTGGCTATTCCAAAAGGTACACCTGTTTGAGAGTA
TGAAAGGTCAGGGTGTTGAGAGAGTTATTAAAAAAAA-
GAAAAAGGCAAAGTAACACTTTAAGTGTTATTTTGTCTTTGATTTTTTTTTT-
CTCTTAGCATCTTGGATATTGGGTG-CTTGCCATTATTAAGTTTGATGG-
CTCACCTTAAATTTATAAGTTGTTTTTT-
ATTAAGGATGAAAAGTCTTTTGATGGCTTGATGTTATTGA-
TATATGAAATGTCATTCATCAAGAAATCAGGGGGGTGACTAACCTTGATGAGAAATGTTGAC
TTTATAAACACATGTTAAAAGATAAATAAAAAGTAAAAGATAAAAAAAATTAAAAATTAAAAA
TTAAAGGGAAAGAAGTAAAAAGAATGAGAGGTATGACATATTTTGAAAATATGTCCCTTG-
TTCTCTTTTTATTTTGATTATCTTTATTTTTTTAT-TTTTAATTTTAATTTTTATGTTTT-
AAGTTTTATTTTATTTTTTACTTCTTTATAATGAATCTCAAA
>TSH-R30145
TTAAGAGTGCACTTAATTGTGGCTTGAAATTTTACTTTATTTACCCCCAACGTC-
TTCGGGACACTGCGGC-
AATTTATTGCTTAGCGAATTTAACCCCTGCGGTTAGGCATATATAA-TTCTCTCT--
GAGGGTTGTATGTGTTCTAAT-CTTGTTTTTTTTATTTT-
CAACCACAAATTTATACATATGTATATATGTATT-ATTTACTATCAAGTAAATAAATAT-
AAAACTTTTAACAATGGATCTCTTGGCTCTCA-
CATCGATGAAGAACACAGTGAAATGTGATAAGTAATGTGAATTGCAGAATTCAGTGAATCAT
CGAATTTTTGAACGCATATTGCGCCTTTTGGCTATTCCAAAAGGTACCCCTGTTTGAGAGTA
TGAAAGGTCAGGGGGTTGAGAGAGTTATTAAAAAAAA-
GAAAAAGGCAAAGTAACCCTTTAAGTGTTATTTTGTCTTTGAATTTTTTTTTTCTCTTAGCA
TCTTGGATATTGGGTG-CTTGCCATTATTAAGTTTGATGG-
CTCACCTTAAATTTATAAGTTGTTTTTT-
ATTAAGGATGAAAAGTCTTTTGATGGCTTGATGTTATTGA-
TATATGAAATGTCATTCATCAAGAAATCAGGGGGGTGACTAACCTTGATGAGAAATGTTGAC
TTTATAAACACATGTTAAAAGATAAATAAAAAGTAAAAGATAAAAAAAATAAAAAATAAAAAA
TTAAAGGGAAAGAAGTAAAAAGAATGAGAGGTATGACATATTTTGAAAATATGTCCCTTG-
TTCTCTTTTTATTTTGATTATCTTTATTTTTTTAT-TTTTAATTTTAATTTTTATGTTTT-
AAGTTTTATTTTATTTTTTACTTCTTTATAATGAATCTCAAA
>TSH-R30148
TTAAGAGTGCACTTAATTGTGGCTTGAAATTTTACTTTATTTACCCCCAACGTC-
TTCGGGACACTGCGGC-
AATTTATTGCTTAGCGAATTTAACCCCTGCGGTTAGGCATATATAA-TTCTCTCT--
GAGGGTTGTATGTGTTCTAATC-TTGTTTTTTTTATTTT-
CAACCACAAATTTATACATATGTATATATGTATT-ATTTACTATCAAGTAAATAAATAT-
AAAACTTTTAACAATGGATCTCTTGGCTCTCA-
CATCGATGAAGAACACAGTGAAATGTGATAAGTAATGTGAATTGCAGAATTCAGTGAATCAT
CGAATTTTTGAACGCATATTGGCCCTTTTGGCTATTCCAAAAGGTACCCCTGTTTGAGAGTA

TGAAAGGTCAGGGTGTTGAGAGAGTTATTAAAAAAAA-
GAAAAAGGCAAAGTAACACTTTAAGTGTTATTTTGTCTTTGATTTTTTTTTTTCTCTTAGCA
TCTTGGATATTGGGTG-CTTGCCATTATTAAGTTTGATGG-
CTCACCTTAAATTTATAAGTTGTTTTTT-
ATTAAGGATGAAAAGTCTTTTGATGGCTTGATGTTATTGA-
TATATGAAATGTCATTCATCAAGAAATCAGGGGGGTGACTAACCTTGATGAGAAATGTTGAC
TTTATAAACACATGTTAAAAGATAAATAAAAAGTAAAAGATAAAAAAAATAAAAAATTAAAAA
TTAAAGGGAAAGAAGTAAAAAGAATGAGAGGTATGACATATTTTGAAAATATGTCCCTTG-
TTCTCTTTTTATTTTGATTATCTTTATTTTTTTAT-TTTTAATTTTAATTTTTATGTTTT-
AAGTTTTATTTTATTTTTTACTTCTTTATAATGAATCTCAAA
>TSH-R30155
TTAAGAGTGCACTTAATTGTGGCTTGAAATTTTACTTTATTTACACCCAACGTC-
TTCGGGACACTGCGGC-
AATTTATTGCTTAGCGAATTTAACCCCTGCGGTTAGGCATATATAA-TTCTCTCT--
GAGGGTTGTATGTGTTCTAAT-CTTGTTTTTTTTATTTT-
CAACCACAAATTTATACATATGTATATATGTATT-ATTTACTATCAAGTAAATAAATAT-
AAAACTTTTAACAATGGATCTCTTGGCTCTCA-
CATCGATGAAGAACACAGTGAAATGTGATAAGTAATGTGAATTGCAGAATTCAGTGAATCAT
CGAATTTTTGAACGCATATTGCGCCTTTTGGCTATTCCAAAAGGTACCCCTGTTTGAGAGTA
TGAAAGGTCAGGGTGTTGAGAGAGTTATTAAAAAAAA-
GAAAAAGGCAAAGTAACCCTTTAAGTGTTATTTTGTCTTTGATTTTTTTTTTTCTCTTAGCA
TCTTGGATATTGGGTG-CTTGCCATTATTAAGTTTGATGG-
CTCACCTTAAATTTATAAGTTGTTTTTT-
ATTAAGGATGAAAAGTCTTTTGATGGCTTGATGTTATTGA-
TATATGAAATGTCATTCATCAAGAAATCAGGGGGGTGACTAACCTTGATGAGAAATGTTGAC
TTTATAAACACATGTTAAAAGATAAATAAAAAGTAAAAGATAAAAAAAATAAAAAATTAAAAA
TTAAAGGGAAAGAAGTAAAAAGAATGAGAGCTATGACATATTTTGAAAATATGTCCCTTG-
TTCTCTTTTTATTTTGATTATCTTTATTTTTTTAT-TTTTAATTTTAATTTTTATGTTTT-
AAGTTTTATTTTATTTTTTACTTCTTTATAATGAATCTCAAA
>TSH-R30160
TTAAGAGTGCACTTAATTGTGGCTTGAAATTTTACTTTATTTACACCCAACGTC-
TTCGGGACACTGCGGC-
AATTTATTGCTTAGCGAATTTAACCCCTGCGGTTAGGCATATATAA-TTCTCTCT--
GAGGGTTGTATGTGTTCTAATCTT-GTTTTTTTTATTTT-
CAACCACAAATTTATACATATGTATATATGTATT-ATTTACTATCAAGTAAATAAATAT-
AAAACTTTTAACAATGGATCTCTTGGCTCTCA-
CATCGATGAAGAACACAGTGAAATGTGATAAGTAATGTGAATTGCAGAATTCAGTGAATCAT
CGAATTTTTGAACGCATATTGCGCCTTTTGGCTATTCCAAAAGGTACACCTGTTTGAGAGTA
TGAAAGGTCAGGGTGTTGAGAGAGTTATTAAAAAAAA-

GAAAAAGGCAAAGTAACACTTTAAGTGTTATTTTGTCTTTGATTTTTTTTTT-
CTCTTAGCATCTTGGATATTGGGTG-CTTGCCATTATTAAGTTTGATGG-
CTCACCTTAAATTTATAAGTTGTTTTTT-
ATTAAGGATGAAAAGTCTTTTGATGGCTTGATGTTATTGA-
TATATGAAATGTCATTCATCAAGAAATCAGGGGGGTGACTAACCTTGATGAGAAATGTTGAC
TTTATAAACACATGTTAAAAGATAAATAAAAAGTAAAAGATAAAAAAAATAAAAAATAAAAAA
TTAAAGGGAAAGAAGTAAAAAGAATGAGAGGTATGACATATTTTGAAAATATGTCCCTTG-
TTCTCTTTTTATTTTGATTATCTTTATTTTTTTAT-
TTTTAATTTTAATTTTTATGTTTTTAAGTTTTATTTTATTTTTTACTTCTTTATAATGAATCT
CAAA

>TSH-R30167

TTAAGAGTGCACTTAATTGTGGCTTGAAATTTTACTTTATTTACACCCAACGTC-
TTCGGGCCACTGCGGC-
AATTTATTGCTTAGCGAATTTAACCCCTGCGGTTAGGCATATATAA-TTCTCTCT--
GAGGGTTGTATGTGTTCTAATCTT-GTTTTTTTTATTTT-
CAACCACAAATTTATACATATGTATATATGTATT-ATTTACTATCAAGTAAATAAATAT-
AAAACTTTTAACAATGGATCTCTTGGCTCTCA-
CATCGATGAAGAACACAGTGAAATGTGATAAGTAATGTGAATTGCAGAATTCAGTGAATCAT
CGAATTTTTGAACGCATATTGCGCCTTTTGGCTATTCCAAAAGGTACACCTGTTTGAGAGTA
TGAAAGGTCAGGGTGTTGAGAGAGTTATTAAAAAAAA-
GAAAAAGGCAAAGTAACACTTTAAGTGTTATTTTGTCTTTGATTTTTTTTTT-
CTCTTAGCATCTTGGATATTGGGTG-CTTGCCATTATTAAGTTTGATGG-
CTCACCTTAAATTTATAAGTTGTTTTTT-
ATTAAGGATGAAAAGTCTTTTGATGGCTTGATGTTATTGA-
TATATGAAATGTCATTCATCAAGAAATCAGGGGGGTGACTAACCTTGATGAGAAATGTTGAC
TTTATAAACACATGTTAAAAGATAAATAAAAAGTAAAAGATAAAAAAAATAAAAAATAAAAAA
TTAAAGGGAAAGAAGTAAAAAGAATGAGAGGTATGACATATTTTGAAAATATGTCCCTTG-
TTCTCTTTTTATTTTGATTATCTTTATTTTTTTAT-TTTTAATTTTAATTTTTATGTTTT-
AAGTTTTATTTTATTTTTTACTTCTTTATAATGAATCTCAAA

>TSH-R30172

TTAAGAGTGCACTTAATTGTGGCTTGAAATTTTACTTTATTTACACCCAACGTC-
TTCGGGACACTGCGGC-
AATTTATTGCTTAGCGAATTTAACCCCTGCGGTTAGGCATATATAA-TTCTCTCT--
GAGGGTTGTATGTGTTCTAATCTT-GTTTTTTTTATTTT-
CAACCACAAATTTATACATATGTATATATGTATT-ATTTACTATCAAGTAAATAAATAT-
AAAACTTTTAACAATGGATCTCTTGGCTCTCA-
CATCGATGAAGAACACAGTGAAATGTGATAAGTAATGTGAATTGCAGAATTCAGTGAATCAT
CGAATTTTTGAACGCATATTGCGCCTTTTGGCTATTCCAAAAGGTACACCTGTTTGAGAGTA
TGAAAGGTCAGGGTGTTGAGAGAGTTATTAAAAAAAA-

GAAAAAGGCAAAGTAACACTTTAAGTGTTATTTTGTCTTTGATTTTTTTTTT-
CTCTTAGCATCTTGGATATTGGGTG-CTTGCCATTATTAAGTTTGATGG-
CTCACCTTAAATTTATAAGTTGTTTTTT-
ATTAAGGATGAAAAGTCTTTTGATGGCTTGATGTTATTGA-
TATATGAAATGTCATTCATCAAGAAATCAGGGGGGTGACTAACCTTGATGAGAAATGTTGAC
TTTATAAACACATGTTAAAAGATAAATAAAAAGTAAAAGATAAAAAAAATAAAAAATAAAAAA
TTAAAGGGAAAGAAGTAAAAAGAATGAGAGGTATGACATATTTTGAAAATATGTCCCTTG-
TTCTCTTTTTATTTTGATTATCTTTATTTTTTTAT-TTTTAATTTTAATTTTTATGTTTT-
AAGTTTTATTTTATTTTTTACTTCTTTATAATGAATCTCAAA
>TSH-R30018
TTAAGAGTGCACTTAATTGTGGCTTGAAATTTTACTTTATTTACACCCAACGTC-
TTCGGGACACTGCGGC-
AATTTATTGCTTAGCGAATTTAACCCCTGCGGTTAGGCATATATAAATTCTCTCT--
GAGGGTGGTATGTGTTCTAATCTT-GTTTTTTTTATTTT-
CAACCACAAATTTATACATATGTATATATGTATT-ATTTACTATCAAGTAAATAAATAT-
AAAACTTTTAACAATGGATCTCTTGGCTCTCA-
CATCGATGAAGAACACAGTGAAATGTGATAAGTAATGTGAATTGCAGAATTCAGTGAATCAT
CGAATTTTTGAACGCATATTGCGCCTTTTGGCTATTCCAAAAGGTACACCTGTTTGAGAGTA
TGAAAGGTCAGGGTGTTGAGAGAGTTATTAAAAAAAA-
GAAAAAGGCAAAGTAACACTTTAAGTGTTATTTTGTCTTTGATTTTTTTTTT-
CTCTTAGCATCTTGGATATTGGGTG-CTTGCCATTATTAAGTTTGATGG-
CTCACCTTAAATTTATAAGTTGTTTTTT-
ATTAAGGATGAAAAGTCTTTTGATGGCTTGATGTTATTGA-
TATATGAAATGTCATTCATCAAGAAATCAGGGGGGTGACTAACCTTGATGAGAAATGTTGAC
TTTATAAACACATGTTAAAAGATAAATAAAAAGTAAAAGATAAAAAAAATAAAAAATTAAAAA
TTAAAGGGAAAGAAGTAAAAAGAATGAGAGGTATGACATATTTTGAAAATATGTCCCTTG-
TTCTCTTTTTATTTTGATTATCTTTATTTTTTTAT-TTTTAATTTTAATTTTTATGTTTT-
AAGTTTTATTTTATTTTTTACTTCTTTATAATGAATCTCAAA
>TSH-R30022
TTAAGAGTGCACTTAATTGTGGCTTGAAATTTTACTTTATTTACACCCAACGTC-
TTCGGGACACTGCGGC-
AATTTATTGCTTAGCGAATTTAACCCCTGCGGTTAGGCATATATAA-TTCTCTCT--
GAGGGTTGTATGTGTTCTAATCTT-GTTTTTTTTATTTT-
CAACCACAAATTTATACATATGTATATATGTATT-ATTTACTATCAAGTAAATAAATAT-
AAAACTTTTAACAATGGATCTCTTGGCTCTCA-
CATCGATGAAGAACACAGTGAAATGTGATAAGTAATGTGAATTGCAGAATTCAGTGAATCAT
CGAATTTTTGAACGCATATTGCGCCTTTTGGCTATTCCAAAAGGTACACCTGTTTGAGAGTA
TGAAAGGTCAGGGTGTTGAGAGAGTTATTAAAAAAAA-
GAAAAAGGCAAAGTAACACTTTAAGTGTTATTTTGTCTTTGATTTTTTTTTT-

CTCTTAGCATCTTGGATATTGGGTG-CTTGCCATTATTAAGTTTGATGG-
CTCACCTTAAATTTATAAGTTGTTTTTT-
ATTAAGGATGAAAAGTCTTTTGATGGCTTGATGTTATTGA-
TATATGAAATGTCATTCATCAAGAAATCAGGGGGGTGACTAACCTTGATGAGAAATGTTGAC
TTTATAAACACATGTTAAAAGATAAATAAAAAGTAAAAGATAAAAAAAATAAAAAATTAAAAA
TTAAAGGGAAAGAAGTAAAAAGAATGAGAGGTATGACATATTTTGAAAATATGTCCCTTG-
TTCTCTTTTTATTTTGATTATCTTTATTTTTTTAT-TTTTAATTTTAATTTTTATGTTTT-
AAGTTTTATTTTATTTTTTACTTCTTTATAATGAATCTCAAA

>TSH-R30024

TTAAGAGTGCACTTAATTGTGGCTTGAAATTTTACTTTATTTACCCCCACCGTC-
TTCGGACACCGGCGGC-
AATTTATTGCTTAGCGAATTTAACCCCTGCGGTTAGGCATATATAA-TTCTCTCT--
GAGGGTTGTAGGTGTTCTAATCTT-GTTTTTTTTATTTT-
CAACCACAAATTTATACATATGTATATATGTATT-ATTTACTATCAAGTAAATAAATAT-
AAAACTTTTAACAATGGATCTCTTGGCTCTCA-
CATCGATGAAGAACACAGTGAAATGTGATAAGTAATGTGAATTGCAGAATTCAGTGAATCAT
CGAATTTTTGAACGCATATTGCGCCTTTTGGCTATTCCAAAAGGTACACCTGTTTGAGAGTA
TGAAAGGTCAGGGTGTTGAGAGAGTTATTAAAAAAAA-
GAAAAAGGCAAAGTAACACTTTAAGTGTTATTTTGTCTTTGATTTTTTTTTT-
CTCTTAGCATCTTGGATATTGGGTG-CTTGCCATTATTAAGTTTGATGG-
CTCACCTTAAATTTATAAGTTGTTTTTT-
ATTAAGGATGAAAAGTCTTTTGATGGCTTGATGTTATTGA-
TATATGAAATGTCATTCATCAAGAAATCAGGGGGGTGACTAACCTTGATGAGAAATGTTGAC
TTTATAAACACATGTTAAAAGATAAATAAAAAGTAAAAGATAAAAAAAATAAAAAATTAAAAA
TTAAAGGGAAAGAAGTAAAAAGAATGAGAGGTATGACATATTTTGAAAATATGTCCCTTG-
TTCTCTTTTTATTTTGATTATCTTTATTTTTTTAT-TTTTAATTTTAATTTTTATGTTTT-
AAGTTTTATTTTATTTTTTACTTCTTTATAATGAACCTCAAA

>TSH-R30028

TTAAGAGTGCACTTAATTGTGGCTTGAAATTTTACTTTATTTACACCCAACGTC-
TTCGGGACACTGCGGC-
AATTTATTGCTTAGCGAATTTAACCCCTGCGGTTAGGCATATATAA-TTCTCTCT--
GAGGGTTGTATGTGTTCTAATCTT-GTTTTTTTTATTTT-
CAACCACAAATTTATACATATGTATATATGTATT-ATTTACTATCAAGTAAATAAATAT-
AAAACTTTTAACAATGGATCTCTTGGCTCTCA-
CATCGATGAAGAACACAGTGAAATGTGATAAGTAATGTGAATTGCAGAATTCAGTGAATCAT
CGAATTTTTGAACGCATATTGCGCCTTTTGGCTATTCCAAAAGGTACACCTGTTTGAGAGTA
TGAAAGGTCAGGGTGTTGAGAGAGTTATTAAAAAAAA-
GAAAAAGGCAAAGTAACACTTTAAGTGTTATTTTGTCTTTGATTTTTTTTTT-
CTCTTAGCATCTTGGATATTGGGTG-CTTGCCATTATTAAGTTTGATGG-

CTCACCTTAAATTTATAAGTTGTTTTTT-
ATTAAGGATGAAAAGTCTTTTGATGGCTTGATGTTATTGA-
TATATGAAATGTCATTCATCAAGAAATCAGGGGGGTGACTAACCTTGATGAGAAATGCTGAC
TTTATAAACACATGTTAAAAGATAAATAAAAAGTAAAAGATAAAAAAAATAAAAAATAAAAAA
TTAAAGGGAAAGAAGTAAAAAGAATGAGAGGTATGACATATTTTGAAAATATGTCCCTTG-
TTCTCTTTTTATTTTGATTATCTTTATTTTTTTAT-TTTTAATCTTAATTTTTATGTTTT-
AAGTTTTATTTTATTTTTTACTTCTTTATAATGAACCTCAAA
>TSH-R30031
TTAAGAGTGCACTTAATTGTGGCTTGAAATTTTACTTTATTTACACCCAACGTC-
TTCGGGACACTGCGGC-
AATTTATTGCTTACGGAATTTAACCCCTGCGGTTAGGCATATATAA-TTCTCTCT--
GAGGGTGGTATGTGTTC-AAATCTTGTTTTTTTTATTTT-
CAACCACAAATTTATACATATGTATATATGTATT-ATTTACTATCAAGTAAATAAATAT-
AAAACTTTTAACAATGGATCTCTTGGCTCTCA-
CATCGATGAAGAACACAGTGAAATGTGATAAGTAATGTGAATTGCAGAATTCAGTGAATCAT
CGAATTTTTGAACGCATATTGCGCCTTTTGGCTATTCCAAAAGGTACACCTGTTTGAGAGTA
TGAAAGGTCAGGGTGTTGAGAGAGTTATTAAAAAAAA-
GAAAAAGGCAAAGTAACACTTTAAGTGTTATTTTGTCTTTGATTTTTTTTTT-
CTCTTAGCATCTTGGATATTGGGTG-CTTGCCATTATTAAGTTTGATGG-
CTCACCTTAAATTTATAAGTTGTTTTTT-
ATTAAGGATGAAAAGTCTTTTGATGGCTTGATGTTATTGA-
TATATGAAATGTCATTCATCAAGAAATCAGGGGGGTGACTAACCTTGATGAGAAATGTTGAC
TTTATAAACACATGTTAAAAGATAAATAAAAAGTAAAAGATAAAAAAAATTAAAAATTAAAAA
TTAAAGGGAAAGAAGTAAAAAGAATGAGAGGTATGACATATTTTGAAAATATGTCCCTTG-
TTCTCTTTTTATTTTGATTATCTTTATTTTTTTAT-TTTTAATTTTAATTTTTATGTTTT-
AAGTTTTATTTTATTTTTTACTTCTTTATAATGAATCTCAAA
>TSH-R30034
TTAAGAGTGCACTTAATTGTGGCTTGAAATTTTACTTAATTTAC-
CCCAACGTCCTTCGGGACACTGCGGC-
AATTTATTGCTAACGAATTTTACCCCCTGCGGTAAGGCATATATAA-TTCTCTCT--
GAGGGTTGTATGTGTTC-AAATCTTGTTTTTTTTATTTT-
CAACCACAAATTTATACATATGTATATATGTATT-ATTTACTATCAAGTAAATAAATAT-
AAAACTTTTAACAATGGATCTCTTGGCTCTCA-
CATCGATGAAGAACACAGTGAAATGTGATAAGTAATGTGAATTGCAGAATTCAGTGAATCAT
CGAATTTTTGAACGCATATTGCGCCTTTTGGCTATTCCAAAAGGTACACCTGTTTGAGAGTA
TGAAAGGTCAGGGTGTTGAGAGAGTTATTAAAAAAAA-
GAAAAAGGCAAAGTAACACTTTAAGTGTTATTTTGTCTTTGATTTTTTTTTT-
CTCTTAGCATCTTGGATATTGGGTG-CTTGCCATTATTAAGTTTGATGG-
CTCACCTTAAATTTATAAGTTGTTTTTT-

ATTAAGGATGAAAAGTCTTTTGATGGCTTGATGTTATTGA-
TATATGAAATGTCATTCATCAAGAAATCAGGGGGGTGACTAACCTTGATGAGAAATGTTGAC
TTTATAAACACTTGTTAAAAGATAAATAAAAAGTAAAAGATAAAAAAAATAAAAAATTAAAAA
TTAAAGGGAAAGAAGTAAAAAGAATGAGAGGTATGACATATTTTGAAAATATGTCCCTTG-
TTCTCTTTTTATTTTGATTATCTTTATTTTTTTAT-TTTTAATTTTAATTTTTATGTTTT-
AAGTTTTATTTTATTTTTTACTTCTTTATAATGAATCTCAAA
>TSH-R30036
TTAAGAGTGCACTTAATTGTGGCTTGAAATTTTACTTTATTTACCCCCAACGTC-
TTCGGGACACTGCGGC-
AATTTATTGCTTAGCGAATTTAACCCCTGCGGTTAGGCATATATAA-TTCTCTCT--
GAGGGTTGTATGTGTTCTAAT-CTTGTTTTTTTTATTTT-
CAACCACAAATTTATACATATGTATATATGTATT-ATTTACTATCAAGTAAATAAATAT-
AAAACTTTTAACAATGGATCTCTTGGCTCTCA-
CATCGATGAAGAACACAGTGAAATGTGATAAGTAATGTGAATTGCAGAATTCAGTGAATCAT
CGAATTTTTGAACGCATATTGCCCCTTTTGGCTATTCCAAAAGGTACACCTGTTTGAGAGTA
TGAAAGGTCAGGGTGTTGAGAGAGTTATTAAAAAAAA-
GAAAAAGGCAAAGTAACCCTTTAAGTGTTATTTTGTCTTTGATTTTTTTTTTTCTCTTAGCA
TCTTGGATATTGGGTG-CTTGCCATTATTAAGTTTGATGG-
CTCACCTTAAATTTATAAGTTGTTTTTT-
ATTAAGGATGAAAAGTCTTTTGATGGCTTGATGTTATTGA-
TATATGAAATGTCATTCATCAAGAAATCAGGGGGGTGACTAACCTTGATGAGAAATGTTGAC
TTTATAAACACATGTTAAAAGATAAATAAAAAGTAAAAGATAAAAAAAATAAAAAATAAAAAA
TTAAAGGGAAAGAAGTAAAAAGAATGAGAGGTATGACATATTTTGAAAATATGTCCCTTG-
TTCTCTTTTTATTTTGATTATCTTTATTTTTTTAT-TTTTAATTTTAATTTTTATGTTTT-
AAGTTTTATTTTATTTTTTACTTCTTTATAATGAATCTCAAA
>TSH-R30042
TTAAGAGTGCACTTAATTGTGGCTTGAAATTTTACTTTATTTACCCCCAACGTC-
TTCGGGACACGGCGGC-
AATTTATTGCTTAGCGAATTTAACCCCTGCGGTTAGGCATATATAA-TTCTCTCT--
GAGGGTTGTATGGGTTCTAATCTT-GTTTTTTTTATTTT-
CAACCACAAATTTATACATATGTATATATGTATT-ATTTACTATCAAGTAAATAAATAT-
AAAACTTTTAACAATGGATCTCTTGGCTCTCA-
CATCGATGAAGAACACAGTGAAATGTGATAAGTAATGTGAATTGCAAAATTCAGTGAATCAT
CGAATTTTTGAACGCATATTGCGCCTTTTGGCTATTCCAAAAGGTACACCTGTTTGAGAGTA
TGAAAGGTCAGGGTGTTGAGAGAGTTATTAAAAAAAA-
GAAAAAGGCAAAGTAACACTTTAAGTGTTATTTTGTCTTTGATTTTTTTTTT-
CTCTTAGCATCTTGGATATTGGGTG-CTTGCCATTATTAAGTTTGATGG-
CTCACCTTAAATTTATAAGTTGTTTTTT-
ATTAAGGATGAAAAGTCTTTTGATGGCTTGATGTTATTGA-

TATATGAAATGTCATTCATCAAGAAATCAGGGGGGTGACTAACCTTGATGAGAAATGTTGAC
TTTATAAACACATGTTAAAAGATAAATAAAAAGTAAAAGATAAAAAAAATTAAAAATTAAAAA
TTAAAGGGAAAGAAGTAAAAAGAATGAGAGGTATGACATATTTTGAAAATATGTCCCTTG-
TTCTCTTTTTATTTTGATTATCTTTATTTTTTTAT-TTTTAATTTTAATTTTTATGTTTT-
AAGTTTTATTTTATTTTTTACTTCTT-ATAATGAATCTCAAA
>TSH-R30043
TTAAGAGTGCACTTAATTGTGGCTTGAAATTTTACTTTATTTACCCCCAACGTC-
TTCGGGACCCTGCGGC-
AATTTATTGCTTAGCGAATTTAACCCCTGCGGTTAGGCATATATAA-TTCTCTCT--
GAGGGTTGTATGTGTTCTAATCTT-GTTTTTTTTATTTT-
CAACCACAAATTTATACATATGTATATATGTATT-ATTTACTATCAAGTAAATAAATAT-
AAAACTTTTAACAATGGATCTCTTGGCTCTCA-
CATCGATGAAGAACACAGTGAAATGTGATAAGTAATGTGAATTGCAGAATTCAGTGAATCAT
CGAATTTTTGAACGCATATTGCGCCTTTTGGCTATTCCAAAAGGTACACCTGTTTGAGAGTA
TGAAAGGTCAGGGTGTTGAGAGAGTTATTAAAAAAAA-
GAAAAAGGCAAAGTAACACTTTAAGTGTTATTTTGTCTTTGATTTTTTTTTT-
CTCTTAGCATCTTGGATATTGGGTG-CTTGCCATTATTAAGTTTGATGG-
CTCACCTTAAATTTATAAGTTGTTTTTT-
ATTAAGGATGAAAAGTCTTTTGATGGCTTGATGTTATTGA-
TATATGAAATGTCATTCATCAAGAAATCAGGGGGGTGACTAACCTTGATGAGAAATGCTGAC
TTTATAAACACATGTTAAAAGATAAATAAAAAGTAAAAGATAAAAAAAATTAAAAATTAAAAA
TTAAAGGGAAAGAAGTAAAAAGAATGAGAGGTATGACATATTTTGAAAATATGTCCCTTG-
TTCTCTTTTTATTTTGATTATCTTTATTTTTTTAT-TTTTAATTTTAATTTTTATGTTTT-
AAGTTTTATTTTATTTTTTACTTCTTTATAATGAATCTCAAA
>TSH-R30045
TTAAGGG-GCACTTAATTGTGGCTTGAA--TTTTCTTTATTTACCCCCAACGTT-
TTGGGGACATTG-GGC-AATTTATTGCTTAGCGAATTTAACCCCTG-GGTTGGCCATAT--AA-
TTCTCTCT-GGAGGGTTGTGTGTGTTCTAAT-TTTGTTTTTTTTATTTT-
CAACCACAAATTTATACATATGTATATATGTATT-ATTTACTATCAAGTAAATAAATAT-
AAAACTTTTAACAATGGATCTCTTGGCTCTCAACTTCGATGAAGAACACAGTGAAATGTGAT
AAGTAATGTGAATTGCAGAATTCAGTGAATCATCGAATTTTTGAACCCATATTGCCCCTTTT
GGCTATTCCAAAAGGCACCCCCTGTTGGGAGTATGAAAGGTCCGGGTGTTGAGAGAGTTTT
TAAAAAAAA-GAAAAAGGCAAAGTAACACTTTAAGTGTTATTTTGTCTTTGATTTTTTTTTT-
CTCTTAGCATCTTGGATATTGGGTG-CTTGCCATTATTAAGTTTGATGG-
CTCACCTTAAATTTATAAGTTGTTTTTT-
ATTAAGGATGAAAAGTCTTTTGATGGCTTGATGTTATTGA-
TATATGAAATGTCATTCATCAAGAAATCAGGGGGGTGACTAACCTTGATGAGAAATGCTGAC
TTTATAAACACATGTTAAAAGATAAATAAAAAGTAAAAGATAAAAAAAATAAAAAATAAAAAA
TTAAAGGGAAAGAAGTAAAAAGAATGAGAGGTATGACATATTTTGAAAATATGTCCCTTG-

TTCTCTTTTTATTTTGATTATCTTTATTTTTTTAT-
TTTTAATTTTAATTTTTATGTTTTGAAGTTTTATTTTATTATTTACTTCTTTATAATGAAACT
CAAA
>TSH-R30046
TTAAGAG-GCACTTAATTGTGGCTTGAAATTTTACTTTATTTACACCCAACGTC-
TTCGGGACACTGCGGC-
AATTTATTGCTTAGCGAATTTAACCCCTGCGGTTAGGCATATATAA-TTCTCTCT--
GAGGGTTGTATGTGTTCTAATCTT-GTTTTTTTTATTTT-
CAACCACAAATTTATACATATGTATATATGTATT-ATTTACTATCAAGTAAATAAATAT-
AAAACTTTTAACAATGGATCTCTTGGCTCTCA-
CATCGATGAAGAACACAGTGAAATGTGATAAGTAATGTGAATTGCAGAATTCAGTGAATCAT
CGAATTTTTGAACGCATATTGCGCCTTTTGGCTATTCCAAAAGGTACACCTGTTTGAGAGTA
TGAAAGGTCAGGGTGTTGAGAGAGTTATTAAAAAAAA-
GAAAAAGGCAAAGTAACACTTTAAGTGTTATTTTGTCTTTGATTTTTTTTTT-
CTCTTAGCATCTTGGATATTGGGTG-CTTGCCATTATTAAGTTTGATGG-
CTCACCTTAAATTTATAAGTTGTTTTTT-
ATTAAGGATGAAAAGTCTTTTGATGGCTTGATGTTATTGA-
TATATGAAATGTCATTCATCAAGAAATCAGGGGGGTGACTAACCTTGATGAGAAATGTTGAC
TTTATAAACACATGTTAAAAGATAAATAAAAAGTAAAAGATAAAAAAAATAAAAAATTAAAAA
TTAAAGGGAAAGAAGTAAAAAGAATGAGAGGTATGACATATTTTGAAAATATGTCCCTTG-
TTCTCTTTTTATTTTGATTATCTTTATTTTTTTAT-TTTTAATTTTAATTTTTATGTTTT-
TAGTTTTAA-----GTTTTAATAGAACCCAATCGATATCAAA
>Mexico-1
TTAAGAGTGCACTTAATTGTGGCTTGAAATTTTACTTTATTTACACCCAACGTC-
TTCGGGACACTGCGGC-
AATTTATTGCTTAGCGAATTTAACCCCTGCGGTTAGGCATATATAA-TTCTCTCT--
GAGGGTTGTATGTGTTCTAATCTT-GTTTTTTTTATTTT-
CAACCACAAATTTATACATATGTATATATGTATT-ATTTACTATCAAGTAAATAAATAT-
AAAACTTTTAACAATGGATCTCTTGGCTCTCA-
CATCGATGAAGAACACAGTGAAATGTGATAAGTAATGTGAATTGCAGAATTCAGTGAATCAT
CGAATTTTTGAACGCATATTGCGCCTTTTGGCTATTCCAAAAGGTACACCTGTTTGAGAGTA
TGAAAGGTCAGGGTGTTGAGAGAGTTATTAAAAAAAA-
GAAAAAGGCAAAGTAACACTTTAAGTGTTATTTTGTCTTTGATTTTTTTTTT-
CTCTTAGCATCTTGGATATTGGGTG-CTTGCCATTATTAAGTTTGATGG-
CTCACCTTAAATTTATAAGTTGTTTTTT-
ATTAAGGATGAAAAGTCTTTTGATGGCTTGATGTTATTGA-
TATATGAAATGTCATTCATCAAGAAATCAGGGGGGTGACTAACCTTGATGAGAAATGCTGAC
TTTATAAACACATGTTAAAAGATAAATAAAAAGTAAAAGATAAAAAAAATAAAAAATAAAAAA
TTAAAGGGAAAGAAGTAAAAAGAATGAGAGGTATGACATATTTTGAAAATATGTCCCTTG-

TTCTCTTTTTATTTTGATTATCTTTATTTTTTTAT-TTTTAATTTTAATTTTTATGTTTT-
AAGTTTTATTTTATTTTTTACTTCTTTATAATGAATCTCAAA
>Mexico-2
TTAAGAGTGCACTTAATTGTGGCTTGAAATTTTACTTTATTTACACCCAACGTC-
TTCGGGACACTGCGGC-
AATTTATTGCTTAGCGAATTTAACCCCTGCGGTTAGGCATATATAA-TTCTCTCT--
GAGGGTTGTATGTGTTCTAATCTT-GTTTTTTTTATTTT-
CAACCACAAATTTATACATATGTATATATGTATT-ATTTACTATCAAGTAAATAAATAT-
AAAACTTTTAACAATGGATCTCTTGGCTCTCA-
CATCGATGAAGAACACAGTGAAATGTGATAAGTAATGTGAATTGCAGAATTCAGTGAATCAT
CGAATTTTTGAACGCATATTGCGCCTTTTGGCTATTCCAAAAGGTACACCTGTTTGAGAGTA
TGAAAGGTCAGGGTGTTGAGAGAGTTATTAAAAAAAA-
GAAAAAGGCAAAGTAACACTTTAAGTGTTATTTTGTCTTTGATTTTTTTTTT-
CTCTTAGCATCTTGGATATTGGGTG-CTTGCCATTATTAAGTTTGATGG-
CTCACCTTAAATTTATAAGTTGTTTTTT-
ATTAAGGATGAAAAGTCTTTTGATGGCTTGATGTTATTGA-
TATATGAAATGTCATTCATCAAGAAATCAGGGGGGTGACTAACCTTGATGAGAAATGCTGAC
TTTATAAACACATGTTAAAAGATAAATAAAAAGTAAAAGATAAAAAAAATAAAAAATAAAAAA
TTAAAGGGAAAGAAGTAAAAAGAATGAGAGGTATGACATATTTTGAAAATATGTCCCTTG-
TTCTCTTTTTATTTTGATTATCTTTATTTTTTTAT-TTTTAATTTTAATTTTTATGTTTT-
AAGTTTTATTTTATTTTTTACTTCTTTATAATGAATCTCAAA
>Mexico-3
TTAAGAGTGCACTTAATTGTGGCTTGAAATTTTACTTTATTTACACCCAACGTC-
TTCGGGACACTGCGGC-
AATTTATTGCTTAGCGAATTTAACCCCTGCGGTTAGGCATATATAA-TTCTCTCT--
GAGGGTTGTATGTGTTCTAATCTT-GTTTTTTTTATTTT-
CAACCACAAATTTATACATATGTATATATGTATT-ATTTACTATCAAGTAAATAAATAT-
AAAACTTTTAACAATGGATCTCTTGGCTCTCA-
CATCGATGAAGAACACAGTGAAATGTGATAAGTAATGTGAATTGCAGAATTCAGTGAATCAT
CGAATTTTTGAACGCATATTGCGCCTTTTGGCTATTCCAAAAGGTACACCTGTTTGAGAGTA
TGAAAGGTCAGGGTGTTGAGAGAGTTATTAAAAAAAA-
GAAAAAGGCAAAGTAACACTTTAAGTGTTATTTTGTCTTTGATTTTTTTTTT-
CTCTTAGCATCTTGGATATTGGGTG-CTTGCCATTATTAAGTTTGATGG-
CTCACCTTAAATTTATAAGTTGTTTTTT-
ATTAAGGATGAAAAGTCTTTTGATGGCTTGATGTTATTGA-
TATATGAAATGTCATTCATCAAGAAATCAGGGGGGTGACTAACCTTGATGAGAAATGCTGAC
TTTATAAACACATGTTAAAAGATAAATAAAAAGTAAAAGATAAAAAAAATAAAAAATAAAAAA
TTAAAGGGAAAGAAGTAAAAAGAATGAGAGGTATGACATATTTTGAAAATATGTCCCTTG-
TTCTCTTTTTATTTTGATTATCTTTATTTTTTTAT-TTTTAATTTTAATTTTTATGTTTT-

AAGTTTTATTTTATTTTTTACTTCTTTATAATGAATCTCAAA
>Mexico-4
TTAAGAGTGCACTTAATTGTGGCTTGAAATTTTACTTTATTTACACCCAACGTC-
TTCGGGACACTGCGGC-
AATTTATTGCTTAGCGAATTTAACCCCTGCGGTTAGGCATATATAA-TTCTCTCT--
GAGGGTTGTATGTGTTCTAATCTTTGTTTTTTTTATTTT-
CAACCACAAATTTATACATATGTATATATGTATT-ATTTACTATCAAGTAAATAAATAT-
AAAACTTTTAACAATGGATCTCTTGGCTCTCA-
CATCGATGAAGAACACAGTGAAATGTGATAAGTAATGTGAATTGCAGAATTCAGTGAATCAT
CGAATTTTTGAACGCATATTGCGCCTTTTGGCTATTCCAAAAGGTACACCTGTTTGAGAGTA
TGAAAGGTCAGGGTGTTGAGAGAGTTATTAA-
AAAAAAGAAAAAGGCAAAGTAACACTTTAAGTGTTATTTTGTCTTTGATTTTTTTTTT-
CTCTTAGCATCTTGGATATTGGGTG-CTTGCCATTATTAAGTTTGATGG-
CTCACCTTAAATTTATAAGTTGTTTTTT-
ATTAAGGATGAAAAGTCTTTTGATGGCTTGATGTTATTGA-
TATATGAAATGTCATTCATCAAGAAATCAGGGGGGTGACTAACCTTGATGAGAAATGTTGAC
TTTATAAACACATGTTAAAAGATAAATAAAAAGTAAAAGATAAAAAAAATAAAAAATAAAAAA
TTAAAGGGAAAGAAGTAAAAAGAATGAGAGGTATGACATATTTTGAAAATATGTCCCTTG-
TTCTCTTTTTATTTTGATTATCTTTATTTTTTTAT-TTTTAATTTTAATTTTTATGTTTT-
AAGTTTTATTTTATTTTTTACTTCTTTATAATGAATCTCAAA
>Mexico-5
TTAAGAGTGCACTTAATTGTGGCTTGAAATTTTACTTTATTTACACCCAACGTC-
TTCGGGACACTGCGGC-
AATTTATTGCTTAGCGAATTTAACCCCGGCGGTTAGGCATATATAA-TTCTCTCT--
GAGGGTTGTATGTGTTCTAATCTTT-TTTTTTTTATTTT-
CAACCACAAATTTATACATATGTATATATGTATT-ATTTACTATCAAGTAAATAAATAT-
AAAACTTTTAACAATGGATCTCTTGGCTCTCA-
CATCGATGAAGAACACAGTGAAATGTGATAAGTAATGTGAATTGCAGAATTCAGTGAATCAT
CGAATTTTTGAACGCATATTGCGCCTTTTGGCTATTCCAAAAGGTACACCTGTTTGAGAGTA
TGAAAGGTCAGGGTGTTGAGAGAGTTATTAA-
AAAAAAGAAAAAGGCAAAGTAACACTTTAAGTGTTATTTTGTCTTTGATTTTTTTTTT-
CTCTTAGCATCTTGGATATTGGGTG-CTTGCCATTATTAAGTTTGATGG-
CTCACCTTAAATTTATAAGTTGTTTTTT-
ATTAAGGATGAAAAGTCTTTTGATGGCTTGATGTTATTGA-
TATATGAAATGTCATTCATCAAGAAATCAGGGGGGTGACTAACCTTGATGAGAAATGTTGAC
TTTATAAACACATGTTAAAAGATAAATAAAAAGTAAAAGATAAAAAAAATAAAAAATAAAAAA
TTAAAGGGAAAGAAGTAAAAAGAATGAGAGGTATGACATATTTTGAAAATATGTCCCTTG-
TTCTCTTTTTATTTTGATTATCTTTATTTTTTTAT-TTTTAATTTTAATTTTTATGTTTT-
AAGTTTTATTTTATTTTTTACTTCTTTATAATGAATCTCAAA

>Mexico-6
TTAAGAGTGCACTTAATTGTGGCTTGAAATTTTACTTTATTTACCCCCAACGTC-
TTCGGGACACTGCGGC-
AATTTATTGCTTAGCGAATTTAACCCCTGCGGTTAGGCATATATAA-TTCTCTCT--
GAGGGTTGTATGTGTTCTAAT-CTTGTTTTTTTATTTT-
CAACCACAAATTTATACATATGTATATATGTATT-ATTTACTATCAAGTAAATAAATAT-
AAAACTTTTAACAATGGATCTCTTGGCTCTCA-
CATCGATGAAGAACACAGTGAAATGTGATAAGTAATGTGAATTGCAGAATTCAGTGAATCAT
CGAATTTTTGAACGCATATTGCGCCTTTTGGCTATTCCAAAAGGTACACCTGTTTGAGAGTA
TGAAAGGTCAGGGTGTTGAGAGAGTTATTAAAAAAAA-
GAAAAAGGCAAAGTAACACTTTAAGTGTTATTTTGTCTTTGATTTTTTTTTT-
CTCTTAGCATCTTGGATATTGGGTG-CTTGCCATTATTAAGTTTGATGG-
CTCACCTTAAATTTATAAGTTGTTTTTT-
ATTAAGGATGAAAAGTCTTTTGATGGCTTGATGTTATTGA-
TATATGAAATGTCATTCATCAAGAAATCAGGGGGGTGACTAACCTTGATGAGAAATGTTGAC
TTTATAAACACATGTTAAAAGATAAATAAAAAGTAAAAGATAAAAAAAATAAAAAATAAAAAA
TTAAAGGGAAAGAAGTAAAAAGAATGAGAGGTATGACATATTTTGAAAATATGTCCCTTG-
TTCTCTTTTTATTTTGATTATCTTTATTTTTTTAT-TTTTAATTTTAATTTTTATGTTTT-
AAGTTTTATTTTATTTTTTACTTCTTTATAATGAATCTCAAA
>Mexico-7
TTAAGAGTGCACTTAATTGTGGCTTGAAATTTTACTTTATTTACACCCAACGTC-
TTCGGGACACTGCGGC-
AATTTATTGCTTAGCGAATTTAACCCCGGCGGTTAGGCATATATAA-TTCTCTCT--
GAGGGTTGTATGTGTTCTAATCTTT-TTTTTTTTATTTT-
CAACCACAAATTTATACATATGTATATATGTATT-ATTTACTATCAAGTAAATAAATAT-
AAAACTTTTAACAATGGATCTCTTGGCTCTCA-
CATCGATGAAGAACACAGTGAAATGTGATAAGTAATGTGAATTGCAGAATTCAGTGAATCAT
CGAATTTTTGAACGCATATTGCGCCTTTTGGCTATTCCAAAAGGTACACCTGTTTGAGAGTA
TGAAAGGTCAGGGTGTTGAGAGAGTTATTAA-
AAAAAAGAAAAAGGCAAAGTAACACTTTAAGTGTTATTTTGTCTTTGATTTTTTTTTT-
CTCTTAGCATCTTGGATATTGGGTG-CTTGCCATTATTAAGTTTGATGG-
CTCACCTTAAATTTATAAGTTGTTTTTT-
ATTAAGGATGAAAAGTCTTTTGATGGCTTGATGTTATTGA-
TATATGAAATGTCATTCATCAAGAAATCAGGGGGGTGACTAACCTTGATGAGAAATGTTGAC
TTTATAAACACATGTTAAAAGATAAATAAAAAGTAAAAGATAAAAAAAATAAAAAATAAAAAA
TTAAAGGGAAAGAAGTAAAAAGAATGAGAGGTATGACATATTTTGAAAATATGTCCCTTG-
TTCTCTTTTTATTTTGATTATCTTTATTTTTTTAT-TTTTAATTTTAATTTTTATGTTTT-
AAGTTTTATTTTATTTTTTACTTCTTTATAATGAATCTCAAA
>Mexico-8

TTAAGAGTGCACTTAATTGTGGCTTGAAATTTTACTTTATTTACACCCAACGTC-
TTCGGGACACTGCGGC-
AATTTATTGCTTAGCGAATTTAACCCCTGCGGTTAGGCATATATAA-TTCTCTCT--
GAGGGTTGTATGTGTTCTAATCTT-GTTTTTTTTATTTT-
CAACCACAAATTTATACATATGTATATATGTATT-ATTTACTATCAAGTAAATAAATAT-
AAAACTTTTAACAATGGATCTCTTGGCTCTCA-
CATCGATGAAGAACACAGTGAAATGTGATAAGTAATGTGAATTGCAGAATTCAGTGAATCAT
CGAATTTTTGAACGCATATTGCGCCTTTTGGCTATTCCAAAAGGTACACCTGTTTGAGAGTA
TGAAAGGTCAGGGTGTTGAGAGAGTTATTAAAAAAAA-
GAAAAAGGCAAAGTAACACTTTAAGTGTTATTTTGTCTTTGATTTTTTTTTT-
CTCTTAGCATCTTGGATATTGGGTG-CTTGCCATTATTAAGTTTGATGG-
CTCACCTTAAATTTATAAGTTGTTTTTT-
ATTAAGGATGAAAAGTCTTTTGATGGCTTGATGTTATTGA-
TATATGAAATGTCATTCATCAAGAAATCAGGGGGGTGACTAACCTTGATGAGAAATGTTGAC
TTTATAAACACATGTTAAAAGATAAATAAAAAGTAAAAGATAAAAAAAATAAAAAATAAAAAA
TTAAAGGGAAAGAAGTAAAAAGAATGAGAGGTATGACATATTTTGAAAATATGTCCCTTG-
TTCTCTTTTTATTTTGATTATCTTTATTTTTTTAT-TTTTAATTTTAATTTTTATGTTTT-
AAGTTTTATTTTATTTTTTACTTCTTTATAATGAATCTCAAA
>Mexico-9
TTAAGAGTGCACTTAATTGTGGCTTGAAATTTTACTTTATTTACACCCAACGTC-
TTCGGGACACTGCGGC-
AATTTATTGCTTAGCGAATTTAACCCCTGCGGTTAGGCATATATAA-TTCTCTCT--
GAGGGTTGTATGTGTTCTAATCTT-GTTTTTTTTATTTT-
CAACCACAAATTTATACATATGTATATATGTATT-ATTTACTATCAAGTAAATAAATAT-
AAAACTTTTAACAATGGATCTCTTGGCTCTCA-
CATCGATGAAGAACACAGTGAAATGTGATAAGTAATGTGAATTGCAGAATTCAGTGAATCAT
CGAATTTTTGAACGCATATTGCGCCTTTTGGCTATTCCAAAAGGTACACCTGTTTGAGAGTA
TGAAAGGTCAGGGTGTTGAGAGAGTTATTAAAAAAAA-
GAAAAAGGCAAAGTAACACTTTAAGTGTTATTTTGTCTTTGATTTTTTTTTT-
CTCTTAGCATCTTGGATATTGGGTG-CTTGCCATTATTAAGTTTGATGG-
CTCACCTTAAATTTATAAGTTGTTTTTT-
ATTAAGGATGAAAAGTCTTTTGATGGCTTGATGTTATTGA-
TATATGAAATGTCATTCATCAAGAAATCAGGGGGGTGACTAACCTTGATGAGAAATGCTGAC
TTTATAAACACATGTTAAAAGATAAATAAAAAGTAAAAGATAAAAAAAATAAAAAATAAAAAA
TTAAAGGGAAAGAAGTAAAAAGAATGAGAGGTATGACATATTTTGAAAATATGTCCCTTG-
TTCTCTTTTTATTTTGATTATCTTTATTTTTTTAT-TTTTAATTTTAATTTTTATGTTTT-
AAGTTTTATTTTATTTTTTACTTCTTTATAATGAATCTCAAA
>Colombia-1
TTAAGAGTGCACTTAATTGTGGCTTGAAATTTTACTTTATTTACACCCAACGTC-

TTCGGGACACTGCGGC-
AATTTATTGCTTAGCGAATTTAACCCCTGCGGTTAGGCATATATAA-TTCTCTCT--
GAGGGTGGTAGGTGTTCTAATCTTGGTTTTTTTTATTTT-
CAACCACAAATTTATACATATGTATATATGTATT-ATTTACTATCAAGTAAATAAATAT-
AAAACTTTTAACAATGGATCTCTTGGCTCTCA-
CATCGATGAAGAACACAGTGAAATGTGATAAGTAATGTGAATTGCAGAATTCAGTGAATCAT
CGAATTTTTGAACGCATATTGCGCCTTTTGGCTATTCCAAAAGGTACACCTGTTTGAGAGTA
TGAAAGGTCAGGGTGTTGAGAGAGTTATTAAAAAAAAAGAAAAAGGCAAAGTAACACTTTAA
GGGTTATTTGGTCTTGGATTTTTTTTT--CTCTTAGCATCTTGGATATTGGGTG-
CTTGCCATTATTAAGTTTGATGG-CTCACCTTAAATTTATAAGTTGTTTTTT-
ATTAAGGATGAAAAGTCTTTTGATGGCTTGATGTTATTGA-
TATATGAAATGTCATTCATCAAGAAATCAGGGGGGTGACTAACCTTGATGAGAAATGTTGAC
TTTATAAACACATGTTAAAAGATAAATAAAAAGTAAAAGATAAAAAAAATTAAAAATTAAAAA
TTAAAGGGAAAGAAGTAAAAAGAATGAGAGGTATGACATATTTTGAAAATATGTCCCTTG-
TTCTCTTTTTATTTTGATTATCTTTATTTTTTTAT-TTTTAATTTTAATTTTTATGTTTT-
AAGTTTTATTTTATTTTTTACTTCTTTATAATGAATCTCAAA
>Colombia-2
TTAAGAGTGCACTTAATTGTGGCTTGAAATTTTACTTTATTTACACCCAACGTC-
TTCGGGACACTGCGGC-
AATTTATTGCTTAGCGAATTTAACCCCTGCGGTTAGGCATATATAA-TTCTCTCT--
GAGGGTGGTATGTGTTCTAATCTTGGTTTTTTTTATTTT-
CAACCACAAATTTATACATATGTATATATGTATT-ATTTACTATCAAGTAAATAAATAT-
AAAACTTTTAACAATGGATCTCTTGGCTCTCA-
CATCGATGAAGAACACAGTGAAATGTGATAAGTAATGTGAATTGCAGAATTCAGTGAATCAT
CGAATTTTTGAACGCATATTGCGCCTTTTGGCTATTCCAAAAGGTACACCTGTTTGAGAGTA
TGAAAGGTCAGGGTGTTGAGAGAGTTATTAAAAAAAAAGAAAAAGGCAAAGTAACACTTTAA
GTGTTATTTTGTCTTTGATTTTTTTTTT-CTCTTAGCATCTTGGATATTGGGTG-
CTTGCCATTATTAAGTTTGATGG-CTCACCTTAAATTTATAAGTTGTTTTTT-
ATTAAGGATGAAAAGTCTTTTGATGGCTTGATGTTATTGA-
TATATGAAATGTCATTCATCAAGAAATCAGGGGGGTGACTAACCTTGATGAGAAATGTTGAC
TTTATAAACACATGTTAAAAGATAAATAAAAAGTAAAAGATAAAAAAAATCAAAAATTAAAAA
TTAAAGGGAAAGAAGTAAAAAGAATGAGAGGTATGACATATTTTGAAAATATGTCCCTTG-
TTCTCTTTTTATTTTGATTATCTTTATTTTTTTAT-TTTTAATTTTAATTTTTATGTTTT-
AAGTTTTATTTTATTTTTTACTTCTTTATAATGAATCTCAAA
>Colombia-3
TTAAGAGTGCACTTAATTGTGGCTTGAAATTTTACTT-ATTTACACCCAACGTC-
TTCGGGACACTGCGGC-
AATTTATTGCTTAGCGAATTTAACCCCTGCGGTTAGGCATATATAA-TTCTCTCT--
GAGGGTTGTATGTGTTCTAATCTTGTTTTTTTTTATTTT-

CAACCACAAATTTATACATATGTATATATGTATT-ATTTACTATCAAGTAAATAAATAT-
AAAACTTTTAACAATGGATCTCTTGGCTCTCA-
CATCGATGAAGAACACAGTGAAATGTGATAAGTAATGTGAATTGCAGAATTCAGTGAATCAT
CGAATTTTTGAACGCATATTGCGCCTTTTGGCTATTCCAAAAGGTACACCTGTTTGAGAGTA
TGAAAGGTCAGGGTGTTGAGAGAGTTATTAAAAAAAAAGAAAAAGGCAAAGTAACACTTTAA
GTGTTATTTTGTCTTTGATTTTTTTTTT-CTCTTAGCATCTTGGATATTGGGTG-
CTTGCCATTATTAAGTTTGATGG-CTCACCTTAAATTTATAAGTTGTTTTTT-
ATTAAGGATGAAAAGTCTTTTGATGGCTTGATGTTATTGA-
TATATGAAATGTCATTCATCAAGAAATCAGGGGGGTGACTAACCTTGATGAGAAATGTTGAC
TTTATAAACACATGTTAAAAGATAAATAAAAAGTAAAAGATAAAAAAAATTAAAAATTAAAAA
TTAAAGGGAAAGAAGTAAAAAGAATGAGAGGTATGACATATTTTGAAAATATGTCCCTTG-
TTCTCTTTTTATTTTGATTATCTTTATTTTTTTAT-TTTTAATTTTAATTTTTATGTTTT-
AAGTTTTATTTTATTTTTTACTTCTTTATAATGAATCTCAAA
>Colombia-4
TTAAGAGTGCACTTAATTGTGGCTTGAAATTTTACTT-ATTTACACCCAACGTC-
TTCGGGACACTGCGGC-
AATTTATTGCTTAGCGAATTTAACCCCTGCGGTTAGGCATATATAA-TTCTCTCT--
GAGGGTTGTATGTGTTCTAATCTTTGTTTTTTTTATTTT-
CAACCACAAATTTATACATATGTATATATGTATT-ATTTACTATCAAGTAAATAAATAT-
AAAACTTTTAACAATGGATCTCTTGGCTCTCA-
CATCGATGAAGAACACAGTGAAATGTGATAAGTAATGTGAATTGCAGAATTCAGTGAATCAT
CGAATTTTTGAACGCATATTGCGCCTTTTGGCTATTCCAAAAGGTACACCTGTTTGAGAGTA
TGAAAGGTCAGGGTGTTGAGAGAGTTATTAAAAAAAA-
GAAAAAGGCAAAGTAACACTTTAAGTGTTATTTTGTCTTTGATTTTTTTTTT-
CTCTTAGCATCTTGGATATTGGGTG-CTTGCCATTATTAAGTTTGATGG-
CTCACCTTAAATTTATAAGTTGTTTTTT-
ATTAAGGATGAAAAGTCTTTTGATGGCTTGATGTTATTGA-
TATATGAAATGTCATTCATCAAGAAATCAGGGGGGTGACTAACCTTGATGAGAAATGTTGAC
TTTATAAACACATGTTAAAAGATAAATAAAAAGTAAAAGATAAAAAAAATTAAAAATTAAAAA
TTAAAGGGAAAGAAGTAAAAAGAATGAGAGGTATGACATATTTTGAAAATATGTCCCTTG-
TTCTCTTTTTATTTTGATTATCTTTATTTTTTTAT-TTTTAATTTTAATTTTTATGTTTT-
AAGTTTTATTTTATTTTTTACTTCTTTATAATGAATCTCAAA
>Colombia-5
TTAAGAGTGCACTTAATTGTGGCTTGAAATTTTACTTTATTTACACCCAACGTC-
TTCGGGACACTGCGGC-
AATTTATTGCTTAGCGAATTTAACCCCTGCGGTTAGGCATATATAA-TTCTCTCT--
GAGGGTTGTATGTGTTCTAATCTTTGTTTTTTTTATTTT-
CAACCACAAATTTATACATATGTATATATGTATT-ATTTACTATCAAGTAAATAAATAT-
AAAACTTTTAACAATGGATCTCTTGGCTCTCA-

CATCGATGAAGAACACAGTGAAATGTGATAAGTAATGTGAATTGCAGAATTCAGTGAATCAT
CGAATTTTTGAACGCATATTGCGCCTTTTGGCTATTCCAAAAGGTACACCTGTTTGAGAGTA
TGAAAGGTCAGGGTGTTGAGAGAGTTATTAAAAAAAA-
GAAAAAGGTAAAGTAACACTTTAAGTGTTATTTTGTCTTTGATTTT-
TTTTTTCTCTTAGCATCTTGGATATTGGGTG-CTTGCCATTATTAAGTTTGATGG-
CTCACCTTAAATTTATAAGTTGTTTTTT-
ATTAAGGATGAAAAGTCTTTTGATGGCTTGATGTTATTGA-
TATATGAAATGTCATTCATCAAGAAATCAGGGGGGTGACTAACCTTGATGAGAAATGTTGAC
TTTATAAACACATGTTAAAAGATAAATAAAAAGTAAAAGATAAAAAAAATTAAAAATTAAAAA
TTAAAGGGAAAGAAGTAAAAAGAATGAGAGGTATGACATATTTTGAAAATATGTCCCTTG-
TTCTCTTTTTATTTTGATTATCTTTATTTTTTTAT-TTTTAATTTTAATTTTTATGTTTT-
AAGTTTTATTTTATTTTTTACTTCTTTATAATGAATCTCAAA
>Colombia-6
TTAAGAGTGCACTTAATTGTGGCTTGAAATTTTACTTTATTTACACCCAACGTC-
TTCGGGACACTGCGGC-
AATTTATTGCTTAGCGAATTTAACCCCTGCGGTTAGGCATATATAA-TTCTCTCT--
GAGGGTTGTATGTGTTCTAATCTTTGTTTTTTTTATTTT-
CAACCACAAATTTATACATATGTATATATGTATT-ATTTACTATCAAGTAAATAAATAT-
AAAACTTTTAACAATGGATCTCTTGGCTCTCA-
CATCGATGAAGAACACAGTGAAATGTGATAAGTAATGTGAATTGCAGAATTCAGTGAATCAT
CGAATTTTTGAACGCATATTGCGCCTTTTGGCTATTCCAAAAGGTACACCTGTTTGAGAGTA
TGAAAGGTCAGGGTGTTGAGAGAGTTATTAAA-
AAAAAGAAAAAGGTAAAGTAACACTTTAAGTGTTATTTTGTCTTTGATTTT-
TTTTTTCTCTTAGCATCTTGGATATTGGGTG-CTTGCCATTATTAAGTTTGATGG-
CTCACCTTAAATTTATAAGTTGTTTTTT-
ATTAAGGATGAAAAGTCTTTTGATGGCTTGATGTTATTGA-
TATATGAAATGTCATTCATCAAGAAATCAGGGGGGTGACTAACCTTGATGAGAAATGTTGAC
TTTATAAACACATGTTAAAAGATAAATAAAAAGTAAAAGATAAAAAAAA-TAAA------
AAATTAAAGGGAAAGAAGTAAAAAGAATGAGAGGTATGACATATTTTGAAAATATGTCCCTT
G-TTCTCTTTTTATTTTGATTATCTTTATTTTTTTAT-TTTTAATTTTAATTTTTATGTTTT-
AAGTTTTATTTTATTTTTTACTTCTTTATAATGAATCTCAAA
>Colombia-7
TTAAGAGTGCACTTAATTGTGGCTTGAAATTTTACTTTATTTACACCCAACGTC-
TTCGGGACACTGCGGC-
AATTTATTGCTTAGCGAATTTAACCCCTGCGGTTAGGCATATATAA-TTCTCTCT--
GAGGGTTGTATGTGTTCTAATCTTTGTTTTTTTTATTTT-
CAACCACAAATTTATACATATGTATATATGTATT-ATTTACTATCAAGTAAATAAATAT-
AAAACTTTTAACAATGGATCTCTTGGCTCTCA-
CATCGATGAAGAACACAGTGAAATGTGATAAGTAATGTGAATTGCAGAATTCAGTGAATCAT

CGAATTTTTGAACGCATATTGCGCCTTTTGGCTATTCCAAAAGGTACACCTGTTTGAGAGTA
TGAAAGGTCAGGGTGTTGAGAGAGTTATTAAAAAAAA-
GAAAAAGGTAAAGTAACACTTTAAGTGTTATTTTGTCTTTGATTT-
ATTTTTTCTCTTAGCATCTTGGATATTGGGTG-CTTGCCATTATTAAGTTTGATGG-
CTCACCTTAAATTTATAAGTTGTTTTTT-
ATTAAGGATGAAAAGTCTTTTGATGGCTTGATGTTATTGA-
TATATGAAATGTCATTCATCAAGAAATCAGGGGGGTGACTAACCTTGATGAGAAATGTTGAC
TTTATAAACACATGTTAAAAGATAAATAAAAAGTAAAAGATAAAAAAAATTAAAAATAAAAAA
TTAAAGGGAAAGAAGTAAAAAGAATGAGAGGTATGACATATTTTGAAAATATGTCCCTTG-
TTCTCTTTTTATTTTGATTATCTTTATTTTTTTAT-TTTTAATTTTAATTTTTATGTTTT-
AAGTTTTATTTTATTTTTTACTTCTTTATAATGAATCTCAAA
>Colombia-8
TTAAGAGTGCACTTAATTGTGGCTTGAAATTTTACTT-ATTTACACCCAACGTC-
TTCGGGACACTGCGGC-
AATTTATTGCTTAGCGAATTTAACCCCTGCGGTTAGGCATATATAA-TTCTCTCT--
GAGGGTTGTATGTGTTCTAATCTTTGTTTTTTTTATTTT-
CAACCACAAATTTATACATATGTATATATGTATT-ATTTACTATCAAGTAAATAAATAT-
AAAACTTTTAACAATGGATCTCTTGGCTCTCA-
CATCGATGAAGAACACAGTGAAATGTGATAAGTAATGTGAATTGCAGAATTCAGTGAATCAT
CGAATTTTTGAACGCATATTGCGCCTTTTGGCTATTCCAAAAGGTACACCTGTTTGAGAGTA
TGAAAGGTCAGGGTGTTGAGAGAGTTATTAAAAAAAA-
GAAAAAGGTAAAGTAACACTTTAAGTGTTATTTTGTCTTTGATTT-
ATTTTTTCTCTTAGCATCTTGGATATTGGGTG-CTTGCCATTATTAAGTTTGATGG-
CTCACCTTAAATTTATAAGTTGTTTTTT-
ATTAAGGATGAAAAGTCTTTTGATGGCTTGATGTTATTGA-
TATATGAAATGTCATTCATCAAGAAATCAGGGGGGTGACTAACCTTGATGAGAAATGTTGAC
TTTATAAACACATGTTAAAAGATAAATAAAAAGTAAAAGATAAAAAA-------
AATAAAAAATTAAAGGGAAAGAAGTAAAAAGAATGAGAGGTATGACATATTTTGAAAATATG
TCCCTTG-TTCTCTTTTTATTTTGATTATCTTTATTTTTTTAT-
TTTTAATTTTAATTTTTATGTTTT-
AAGTTTTATTTTATTTTTTACTTCTTTATAATGAATCTCAAA
>Colombia-9
TTAAGAGTGCACTTAATTGTGGCTTGAAATTTTACTTTATTTACACCCAACGTC-
TTCGGGACACTGCGGC-
AATTTATTGCTTAGCGAATTTAACCCCTGCGGTTAGGCATATATAA-TTCTCTCT--
GAGGGTTGTATGTGTTCTAATCTT-GTTTTTTTTATTTT-
CAACCACAAATTTATACATATGTATATATGTATT-ATTTACTATCAAGTAAATAAATAT-
AAAACTTTTAACAATGGATCTCTTGGCTCTCA-
CATCGATGAAGAACACAGTGAAATGTGATAAGTAATGTGAATTGCAGAATTCAGTGAATCAT

CGAATTTTTGAACGCATATTGCGCCTTTTGGCTATTCCAAAAGGTACACCTGTTTGAGAGTA
TGAAAGGTCAGGGTGTTGAGAGAGTTATTAAAAAAAA-
GAAAAAGGTAAAGTAACACTTTAAGTGTTATTTTGTCTTTGATTT-
ATTTTTTCTCTTAGCATCTTGGATATTGGGTG-CTTGCCATTATTAAGTTTGATGG-
CTCACCTTAAATTTATAAGTTGTTTTTT-
ATTAAGGATGAAAAGTCTTTTGATGGCTTGATGTTATTGA-
TATATGAAATGTCATTCATCAAGAAATCAGGGGGGTGACTAACCTTGATGAGAAATGTTGAC
TTTATAAACACATGTTAAAAGATAAATAAAAAGTAAAAGATAAAAAAAATAAAAAATTAAAAA
TTAAAGGGAAAGAAGTAAAAAGAATGAGAGGTATGACATATTTTGAAAATATGTCCCTTG-
TTCTCTTTTTATTTTGATTATCTTTATTTTTTTAT-TTTTAATTTTAATTTTTATGTTTT-
AAGTTTTATTTTATTTTTTACTTCTTTATAATGAATCTCAAA
>Colombia-10
TTAAGAGTGCACTTAATTGTGGCTTGAAATTTTACTTTATTTACACCCAACGTC-
TTCGGGACACTGCGGC-
AATTTATTGCTTAGCGAATTTAACCCCTGCGGTTAGGCATATATAA-TTCTCTCT--
GAGGGTTGTATGTGTTCTAATCTT-GTTTTTTTTATTTT-
CAACCACAAATTTATACATATGTATATATGTATT-ATTTACTATCAAGTAAATAAATAT-
AAAACTTTTAACAATGGATCTCTTGGCTCTCA-
CATCGATGAAGAACACAGTGAAATGTGATAAGTAATGTGAATTGCAGAATTCAGTGAATCAT
CGAATTTTTGAACGCATATTGCGCCTTTTGGCTATTCCAAAAGGTACACCTGTTTGAGAGTA
TGAAAGGTCAGGGTGTTGAGAGAGTTATTAAAAAAAA-
GAAAAAGGTAAAGTAACACTTTAAGTGTTATTTTGTCTTTGATTT-
ATTTTTTCTCTTAGCATCTTGGATATTGGGTG-CTTGCCATTATTAAGTTTGATGG-
CTCACCTTAAATTTATAAGTTGTTTTTT-
ATTAAGGATGAAAAGTCTTTTGATGGCTTGATGTTATTGA-
TATATGAAATGTCATTCATCAAGAAATCAGGGGGGTGACTAACCTTGATGAGAAATGTTGAC
TTTATAAACACATGTTAAAAGATAAATAAAAAGTAAAAGATAAAAAAA-------
ATAAAAAATTAAAGGGAAAGAAGTAAAAAGAATGAGAGGTATGACATATTTTGAAAATATGT
CCCTTG-TTCTCTTTTTATTTTGATTATCTTTATTTTTTTAT-
TTTTAATTTTAATTTTTATGTTTT-
AAGTTTTATTTTATTTTTTACTTCTTTATAATGAATCTCAAA
>CIFC-1
TTAAGAGTGCACTTAATTGTGGCTTGAAATTTTACTT-ATTTACACCCAACGTC-
TTCGGGACACTGCGGC-
AATTTATTGCTTAGCGAATTTAACCCCTGCGGTTAGGCATATATAA-TTCTCTCT--
GAGGGTTGTATGTGTTCTAATCTT-GTTTTTTTTATTTT-
CAACCACAAATTTATACATATGTATATATGTATT-ATTTACTATCAAGTAAATAAATAT-
AAAACTTTTAACAATGGATCTCTTGGCTCTCA-
CATCGATGAAGAACACAGTGAAATGTGATAAGTAATGTGAATTGCAGAATTCAGTGAATCAT

CGAATTTTTGAACGCATATTGCGCCTTTTGGCTATTCCAAAAGGTACACCTGTTTGAGAGTA
TGAAAGGTCAGGGTGTTGAGAGAGTTATTAAAAAAAA-
GAAAAAGGCAAAGTAACACTTTAAGTGTTATTTTGTCTTTGATTTTTTTTT--
CTCTTAGCATCTTGGATATTGGGTG-CTTGCCATTATTAAGTTTGATGG-
CTCACCTTAAATTTATAAGTTGTTTTTT-
ATTAAGGATGAAAAGTCTTTTGATGGCTTGATGTTATTGA-
TATATGAAATGTCATTCATCAAGAAATCAGGGGGGTGACTAACCTTGATGAGAAATGCTGAC
TTTATAAACACATGTTAAAAGATAAATAAAAAGTAAAAGATAAAAAAAATAAAAAATAAAAAA
TTAAAGGGAAAGAAGTAAAAAGAATGAGAGGTATGACATATTTTGAAAATATGTCCCTTG-
TTCTCTTTTTATTTTGATTATCTTTATTTTTTTAT-TTTTAATTTTAATTTTTATGTTTT-
AAGTTTTATTTTATTTTTTACTTCTTTATAATGAATCTCAAA
>CIFC-2
TTAAGAGTGCACTTAATTGTGGCTTGAAATTTTACTTTATTTACACCCAACGTC-
TTCGGGACACTGCGGC-
AATTTATTGCTTAGCGAATTTAACCCCTGCGGTTAGGCATATATAA-TTCTCTCT--
GAGGGTTGTATGTGTTCTAATCTT-TTTTTTTTTATTTT-
CAACCACAAATTTATACATATGTATATATGTATT-ATTTACTATCAAGTAAATAAATAT-
AAAACTTTTAACAATGGATCTCTTGGCTCTCA-
CATCGATGAAGAACACAGTGAAATGTGATAAGTAATGTGAATTGCAGAATTCAGTGAATCAT
CGAATTTTTGAACGCATATTGCGCCTTTTGGCTATTCCAAAAGGTACACCTGTTTGAGAGTA
TGAAAGGTCAGGGTGTTGAGAGAGTTATTAAAAAAAA-
GAAAAAGGCAAAGTAACACTTTAAGTGTTATTTTGTCTTTGATTTTTTTTTT-
CTCTTAGCATCTTGGATATTGGGTG-CTTGCCATTATTAAGTTTGATGG-
CTCACCTTAAATTTATAAGTTGTTTTTT-
ATTAAGGATGAAAAGTCTTTTGATGGCTTGATGTTATTGA-
TATATGAAATGTCATTCATCAAGAAATCAGGGGGGTGACTAACCTTGATGAGAAATGCTGAC
TTTATAAACACATGTTAAAAGATAAATAAAAAGTAAAAGATAAAAAAAATAAAAAATAAAAAA
TTAAAGGGAAAGAAGTAAAAAGAATGAGAGGTATGACATATTTTGAAAATATGTCCCTTG-
TTCTCTTTTTATTTTGATTATCTTTATTTTTTTAT-TTTTAATTTTAATTTTTATGTTTT-
AAGTTTTATTTTATTTTTTACTTCTTTATAATGAATCTCAAA
>CIFC-3
TTAAGAGTGCACTTAATTGTGGCTTGAAATTTTACTT-ATTTACACCCAACGTC-
TTCGGGACACTGCGGC-
AATTTATTGCTTAGCGAATTTAACCCCTGCGGTTAGGCATATATAA-TTCTCTCT--
GAGGGTTGTATGTGTTCTAATCTTTGTTTTTTTTTATTTT-
CAACCACAAATTTATACATATGTATATATGTATT-ATTTACTATCAAGTAAATAAATAT-
AAAACTTTTAACAATGGATCTCTTGGCTCTCA-
CATCGATGAAGAACACAGTGAAATGTGATAAGTAATGTGAATTGCAGAATTCAGTGAATCAT
CGAATTTTTGAACGCATATTGCGCCTTTTGGCTATTCCAAAAGGTACACCTGTTTGAGAGTA

TGAAAGGTCAGGGTGTTGAGAGAGTTATTAAAAAAAA-
GAAAAAGGCAAAGTAACACTTTAAGTGTTATTTTGTCTTTGATTTTTTTTT--
CTCTTAGCATCTTGGATATTGGGTG-CTTGCCATTATTAAGTTTGATGG-
CTCACCTTAAATTTATAAGTTGTTTTTT-
ATTAAGGATGAAAAGTCTTTTGATGGCTTGATGTTATTGA-
TATATGAAATGTCATTCATCAAGAAATCAGGGGGGTGACTAACCTTGATGAGAAATGCTGAC
TTTATAAACACATGTTAAAAGATAAATAAAAAGTAAAAGATAAAAAAAATTAAAAATTAAAAA
TTAAAGGGAAAGAAGTAAAAAGAATGAGAGGTATGACATATTTTGAAAATATGTCCCTTG-
TTCTCTTTTTATTTTGATTATCTTTATTTTTTTAT-TTTTAATTTTAATTTTTATGTTTT-
AAGTTTTATTTTATTTTTTACTTCTTTATAATGAATCTCAAA
>Brazil-1
TTAAGAGTGCACTTAATTGTGGCTTGAAATTTTACTTTATTTACACCCAACGTC-
TTCGGGACACTGCGGC-
AATTTATTGCTTAGCGAATTTAACCCCTGCGGTTAGGCATATATAA-TTCTCTCT--
GAGGGTTGTATGTGTTCTAATCTTTGTTTTTTTTATTTT-
CAACCACAAATTTATACATATGTATATATGTATT-ATTTACTATCAAGTAAATAAATAT-
AAAACTTTTAACAATGGATCTCTTGGCTCTCA-
CATCGATGAAGAACACAGTGAAATGTGATAAGTAATGTGAATTGCAGAATTCAGTGAATCAT
CGAATTTTTGAACGCATATTGCGCCTTTTGGCTATTCCAAAAGGTACACCTGTTTGAGAGTA
TGAAAGGTCAGGGTGTTGAGAGAGTTATTAAGAAAAAAGAAAAAGGCAAAGTAACACTTTAA
GTGTTATTTTGTCTTTGATTTTTTTTTTTCTCTTAGCATCTTGGATATTGGGTG-
CTTGCCATTATTAAGTTTGATGG-
CTCACCTTAAATTTATAAGTTGTTTTTTTATTAAGGATGAAAAGTCTTTTGATGGCTTGATG
TTATTGA-
TATATGAAATGTCATTCATCAAGAAATCAGGGGGGTGACTAACCTTGATGAGAAATGTTGAC
TTTATAAACACATGTTAAAAGATAAATAAAAAGTAAAAGATAAAAAAAATAAAAAATAAAAAA
TTAAAGGGAAAGAAGTAAAAAGAATGAGAGGTATGACATATTTTGAAAATATGTCCCTTG-
TTCTCTTTTTATTTTGATTATCTTTATTTTTTTAT-TTTTAATTTTAATTTTTATGTTTT-
AAGTTTTATTTTATTTTTTACTTCTTTATAATGAATCTCAAA
>Brazil-2
TTAAGAGTGCACTTAATTGTGGCTTGAAATTTTACTTTATTTACACCCAACGTC-
TTCGGGACACTGCGGC-
AATTTATTGCTTAGCGAATTTAACCCCTGCGGTTAGGCATATATAA-TTCTCTCT--
GAGGGTTGTATGTGTTCTAATCTTTGTTTTTTTTATTTT-
CAACCACAAATTTATACATATGTATATATGTATT-ATTTACTATCAAGTAAATAAATAT-
AAAACTTTTAACAATGGATCTCTTGGCTCTCA-
CATCGATGAAGAACACAGTGAAATGTGATAAGTAATGTGAATTGCAGAATTCAGTGAATCAT
CGAATTTTTGAACGCATATTGCGCCTTTTGGCTATTCCAAAAGGTACACCTGTTTGAGAGTA
TGAAAGGTCAGGGTGTTGAGAGAGTTATTAAAAAAAAAGAAAAAGGCAAAGTAACACTTTAA

GTGTTATTTTGTCTTTGATTTTTTTTTTTCTCTTAGCATCTTGGATATTGGGTG-
CTTGCCATTATTAAGTTTGATGG-
CTCACCTTAAATTTATAAGTTGTTTTTTTATTAAGGATGAAAAGTCTTTTGATGGCTTGATG
TTATTGA-
TATATGAAATGTCATTCATCAAGAAATCAGGGGGGTGACTAACCTTGATGAGAAATGTTGAC
TTTATAAACACATGTTAAAAGATAAATAAAAAGTAAAAGATAAAAAAAATTAAAAATTAAAAA
TTAAAGGGAAAGAAGTAAAAAGAATGAGAGGTATGACATATTTTGAAAATATGTCCCTTG-
TTCTCTATTTATTTTGATTATCTTTATTTTTTTAT-TTTTAATTTTAATTTTTATGTTTT-
AAGTTTTATTTTATTTTTTACTTCTTTATAATGAATCTCAAA
>Brazil-3
TTAAGAGTGCACTTAATTGTGGCTTGAAATTTTACTT-ATTTACACCCAACGTC-
TTCGGGACACTGCGGC-
AATTTATTGCTTAGCGAATTTAACCCCTGCGGTTAGGCATATATAA-TTCTCTCT--
GAGGGTTGTATGTGTTCTAATCTTTGTTTTTTTTATTTT-
CAACCACAAATTTATACATATGTATATATGTATT-ATTTACTATCAAGTAAATAAATAT-
AAAACTTTTAACAATGGATCTCTTGGCTCTCA-
CATCGATGAAGAACACAGTGAAATGTGATAAGTAATGTGAATTGCAGAATTCAGTGAATCAT
CGAATTTTTGAACGCATATTGCGCCTTTTGGCTATTCCAAAAGGTACACCTGTTTGAGAGTA
TGAAAGGTCAGGGTGTTGAGAGAGTTATTAAAAAAAAAGAAAAAGGTAAAGTAACACTTTAA
GTGTTATTTTGTCTTTGATTTTATTTTTTCTCTTAGCATCTTGGATATTGGGTG-
CTTGCCATTATTAAGTTTGATGG-
CTCACCTTAAATTTATAAGTTGTTTTTTTATTAAGGATGAAAAGTCTTTTGATGGCTTGATG
TTATTGA-
TATATGAAATGTCATTCATCAAGAAATCAGGGGGGTGACTAACCTTGATGAGAAATGTTGAC
TTTATAAACACATGTTAAAAGATAAATAAAAAGTAAAAGATAAAAAAAATTAAAAATTAAAAA
TTAAAGGGAAAGAAGTAAAAAGAATGAGAGGTATGACATATTTTGAAAATATGTCCCTTG-
TTCTCTTTTTATTTTGATTATCTTTATTTTTTTAT-TTTTAATTTTAATTTTTATGTTTT-
AAGTTTTATTTTATTTTTTACTTCTTTATAATGAATCTCAAA
>Brazil-4
TTAAGAGTGCACTTAATTGTGGCTTGAAATTTTACTT-ATTTACACCCAACGTC-
TTCGGGACACTGCGGC-
AATTTATTGCTTAGCGAATTTAACCCCTGCGGTTAGGCATATATAA-TTCTCTCT--
GAGGGTTGTATGTGTTCTAATCTTTGTTTTTTTTATTTT-
CAACCACAAATTTATACATATGTATATATGTATT-ATTTACTATCAAGTAAATAAATAT-
AAAACTTTTAACAATGGATCTCTTGGCTCTCA-
CATCGATGAAGAACACAGTGAAATGTGATAAGTAATGTGAATTGCAGAATTCAGTGAATCAT
CGAATTTTTGAACGCATATTGCGCCTTTTGGCTATTCCAAAAGGTACACCTGTTTGAGAGTA
TGAAAGGTCAGGGTGTTGAGAGAGTTATTAAAAAAAAAGAAAAAGGCAAAGTAACACTTTAA
GTGTTATTTTGTCTTTGATTTTTTTTTTTCTCTTAGCATCTTGGATATTGGGTG-

CTTGCCATTATTAAGTTTGATGG-
CTCACCTTAAATTTATAAGTTGTTTTTTTATTAAGGATGAAAAGTCTTTTGATGGCTTGATG
TTATTGA-
TATATGAAATGTCATTCATCAAGAAATCAGGGGGGTGACTAACCTTGATGAGAAATGTTGAC
TTTATAAACACATGTTAAAAGATAAATAAAAAGTAAAAGATAAAAAAAATTAAAAATTAAAAA
TTAAAGGGAAAGAAGTAAAAAGAATGAGAGGTATGACATATTTTAAAAATATGTCCCTTG-
TTCTCTTTTTATTTTGATTATCTTTATTTTTTTAT-TTTTAATTTTAATTTTTATGTTTT-
AAGTTTTATTTTATTTTTTACTTCTTTATAATGAATCTCAAA
>Brazil-5
TTAAGAGTGCACTTAATTGTGGCTTGAAATTTTACTT-ATTTACACCCAACGTC-
TTCGGGACACTGCGGC-
AATTTATTGCTTAGCGAATTTAACCCCTGCGGTTAGGCATATATAA-TTCTCTCT--
GAGGGTTGTATGTGTTCTAATCTTTGTTTTTTTTATTTT-
CAACCACAAATTTATACATATGTATATATGTATT-ATTTACTATCAAGTAAATAAATAT-
AAAACTTTTAACAATGGATCTCTTGGCTCTCA-
CATCGATGAAGAACACAGTGAAATGTGATAAGTAATGTGAATTGCAGAATTCAGTGAATCAT
CGAATTTTTGAACGCATATTGCGCCTTTTGGCTATTCCAAAAGGTACACCTGTTTGAGAGTA
TGAAAGGTCAGGGTGTTGAGAGAGTTATTAAAAAAAAAGAAAAAGGCAAAGTAACACTTTAA
GTGTTATTTTGTCTTTGATTTTTTTTTTTCTCTTAGCATCTTGGATATTGGGTG-
CTTGCCATTATTAAGTTTGATGG-
CTCACCTTAAATTTATAAGTTGTTTTTTTATTAAGGATGAAAAGTCTTTTGATGGCTTGATG
TTATTGA-
TATATGAAATGTCATTCATCAAGAAATCAGGGGGGTGACTAACCTTGATGAGAAATGTTGAC
TTTATAAACACATGTTAAAAGATAAATAAAAAGTAAAAGATAAAAAAAATTAAAAATTAAAAA
TTAAAGGGAAAGAAGTAAAAAGAATGAGAGGTATGACATATTTTGAAAATATGTCCCTTG-
TTCTCTTTTTATTTTGATTATCTTTATTTTTTTAT-TTTTAATTTTAATTTTTATGTTTT-
AAGTTTTATTTTATTTTTTACTTCTTTATAATGAATCTCAAA
>Brazil-6
TTAAGAGTGCACTTAATTGTGGCTTGAAATTTTACTT-ATTTACACCCAACGTC-
TTCGGGACACTGCGGC-
AATTTATTGCTTAGCGAATTTAACCCCTGCGGTTAGGCATATATAA-TTCTCTCT--
GAGGGTTGTATGTGTTCTAATCTTTGTTTTTTTTATTTT-
CAACCACAAATTTATACATATGTATATATGTATT-ATTTACTATCAAGTAAATAAATAT-
AAAACTTTTAACAATGGATCTCTTGGCTCTCA-
CATCGATGAAGAACACAGTGAAATGTGATAAGTAATGTGAATTGCAGAATTCAGTGAATCAT
CGAATTTTTGAACGCATATTGCGCCTTTTGGCTATTCCAAAAGGTACACCTGTTTGAGAGTA
TGAAAGGTCAGGGTGTTGAGAGAGTTATTAAAAAAAAAGAAAAAGGCAAAGTAACACTTTAA
GTGTTATTTTGTCTTTGATTTTTTTTTTTCTCTTAGCATCTTGGATATTGGGTG-
CTTGCCATTATTAAGTTTGATGG-

CTCACCTTAAATTTATAAGTTGTTTTTTTATTAAGGATGAAAAGTCTTTTGATGGCTTGATG
TTATTGA-
TATATGAAATGTCATTCATCAAGAAATCAGGGGGGTGACTAACCTTGATGAGAAATGTTGAC
TTTATAAACACATGTTAAAAGATAAATAAAAAGTAAAAGATAAAAAAAATTAAAAATTAAAAA
TTAAAGGGAAAGAAGTAAAAAGAATGAGAGGTATGACATATTTTGAAAATATGTCCCTTG-
TTCTCTTTTTATTTTGATTATCTTTATTTTTTTAT-TTTTAATTTTAATTTTTATGTTTT-
AAGTTTTATTTTATTTTTTACTTCTTTATAATGAATCTCAAA

>Brazil-7

TTAAGAGTGCACTTAATTGTGGCTTGAAATTTTACTT-ATTTACACCCAACGTC-
TTCGGGACACTGCGGC-
AATTTATTGCTTAGCGAATTTAACCCCTGCGGTTAGGCATATATAA-TTCTCTCT--
GAGGGTTGTATGTGTTCTAATCTTTGTTTTTTTTATTTT-
CAACCACAAATTTATACATATGTATATATGTATT-ATTTACTATCAAGTAAATAAATAT-
AAAACTTCTAACAATGGATCTCTTGGCTCTCA-
CATCGATGAAGAACACAGTGAAATGTGATAAGTAATGTGAATTGCAGAATTCAGTGAATCAT
CGAATTTTTGAACGCATATTGCGCCTTTTGGCTATTCCAGAAGGTACACCTGTTTGAGAGT
ATGAAAGGTCAGGGTGTTGAGAGAGTTATTAAAAAAAAAGAAAAAGGCAAAGTAACACTTTA
AGTGTTATTTTGTCTTTGATTTTTTTTTTTCTCTTAGCATCTTGGATATTGGGTG-
CTTGCCATTATTAAGTTTGATGG-
CTCACCTTAAATTTATAAGTTGTTTTTTTATTAAGGATGAAAAGTCTTTTGATGGCTTGATG
TTATTGA-
TATATGAAATGTCATTCATCAAGAAATCAGGGGGGTGACTAACCTTGATGAGAAATGTTGAC
TTTATAAACACATGTTAAAAGATAAATAAAAAGTAAAAGATAAAAAAAATAAAAAATAAAAAA
TTAAAGGGAAAGAAGTAAAAAGAATGAGAGGTATGACATATTTTGAAAATATGTCCCTTG-
TTCTCTTTTTATTTTGATTATCTTTATTTTTTTAT-TTTTAATTTTAATTTTTATGTTTT-
AAGTTTTATTTTATTTTTTACTTCTTTATAATGAATCTCAAA

>Brazil-8

TTAAGAGTGCACTTAATTGTGGCTTGAAATTTTACTTTATTTACACCCAACGTC-
TTCGGGACACTGCGGC-
AATTTATTGCTTAGCGAATTTAACCCCTGTGGTTAGGCATATATAA-TTCTCTCT--
GAGGGTTGTATGTGTTCTAATCTTTGTTTTTTTTATTTT-
CAACCACAAATTTATACATATGTATATATGTATT-ATTTACTATCAAGTAAATAAATAT-
AAAACTTTTAACAATGGATCTCTTGGCTCTCA-
CATCGATGAAGAACACAGTGAAATGTGATAAGTAATGTGAATTGCAGAATTCAGTGAATCAT
CGAATTTTTGAACGCATATTGCGCCTTTTGGCTATTCCAAAAGGTACACCTGTTTGAGAGTA
TGAAAGGTCAGGGTGTTGAGAGAGTTATTAAAAAAAAAGAAAAAGGCAAAGTAACACTTTAA
GTGTTATTTTGTCTTTGATTTTTTTTTTTCTCTTAGCATCTTGGATATTGGGTG-
CTTGCCATTATTAAGTTTGATGG-
CTCACCTTAAATTTATAAGTTGTTTTTTTATTAAGGATGAAAAGTCTTTTGATGGCTTGATG

TTATTGA-
TATATGAAATGTCATTCATCAAGAAATCAGGGGGGTGACTAACCTTGATGAGAAATGTTGAC
TTTATAAACACATGTTAAAAGATAAATAAAAAGTAAAAGATAAAAAAAATAAAAAATAAAAAA
TTAAAGGGAAAGAAGTAAAAAGAATGAGAGGTATGACATATTTTGAAAATATGTCCCTTG-
TTCTCTTTTTATTTTGATTATCTTTATTTTTTTAT-TTTTAATTTTAATTTTTATGTTTT-
AAGTTTTATTTTATTTTTTACTTCTTTATAATGAATCTCAAA
>Brazil-9
TTAAGAGTGCACTTAATTGTGGCTTGAAATTTTACTTTATTTACACCCAACGTC-
TTCGGGACACTGCGGC-
AATTTATTGCTTAGCGAATTTAACCCCTGCGGTTAGGCATATATAA-TTCTCTCT--
GAGGGTTGTATGTGTTCTAATCTTTGTTTTTTTTATTTT-
CAACCACAAATTTATACATATGTATATATGTATT-ATTTACTATCAAGTAAATAAATAT-
AAAACTTTTAACAATGGATCTCTTGGCTCTCA-
CATCGATGAAGAACACAGTGAAATGTGATAAGTAATGTGAATTGCAGAATTCAGTGAATCAT
CGAATTTTTGAACGCATATTGCGCCTTTTGGCTATTCCAAAAGGTACACCTGTTTGAGAGTA
TGAAAGGTCAGGGTGTTGAGAGAGTTATTAAAAAAAAAGAAAAAGGTAAAGTAACACTTTAA
GTGTTATTTTGTCTTTGATTTTATTTTTTCTCTTAGCATCTTGGATATTGGGTG-
CTTGCCATTATTAAGTTTGATGG-
CTCACCTTAAATTTATAAGTTGTTTTTTTATTAAGGATGAAAAGTCTTTTGATGGCTTGATG
TTATTGA-
TATATGAAATGTCATTCATCAAGAAATCAGGGGGGTGACTAACCTTGATGAGAAATGTTGAC
TTTATAAACACATGTTAAAAGATAAATAAAAAGTAAAAGATAAAAAAAGTAAAAAATAAAAAA
TTAAAGAGAAAGAAGTAAAAAGAATGAGAGGTATGACATATTTTGAAAATATGTCCCTTG-
TTCTCTTTTTATTTTGATTATCTTTATTTTTTTAT-TTTTAATTTTAATTTTTATGTTTT-
AAGTTTTATTTTATTTTTTACTTCTTTATAATGAATCTCAAA
>Brazil-10
TTAAGAGTGCACTTAATTGTGGCTTGAAATTTTACTTTATTTACACCCAACGTC-
TTCGGGACACTGCGGC-
AATTTATTGCTTAGCGAATTTAACCCCTGCGGTTAGGCATATATAA-TTCTCTCT--
GAGGGTTGTATGTGTTCTAATCTTTGTTTTTTTTATTTT-
CAACCACAAATTTATACATATGTATATATGTATT-ATTTACTATCAAGTAAATAAATAT-
AAAACTTTTAACAATGGATCTCTTGGCTCTCA-
CATCGATGAAGTACACAGTGAAATGTGATAAGTAATGTGAATTGCAGAATTCAGTGAATCAT
CGAATTTTTGAACGCATATTGCGCCTTTTGGCTATTCCAAAAGGTACACCTGTTTGAGAGTA
TGAAAGGTCAGGGTGTTGAGAGAGTTATTAAAAAAAAAGAAAAAGGCAAAGTAACACTTTAA
GTGTTATTTTGTCTTTGATTTTTTTTTTTCTCTTAGCATCTTGGATATTGGGTG-
CTTGCCATTATTAAGTTTGATGG-
CTCACCTTAAATTTATAAGTTGTTTTTTTATTAAGGATGAAAAGTCTTTTGATGGCTTGATG
TTATTGA-

TATATGAAATGTCATTCATCAAGAAATCAGGGGGGTGACTAACCTTGATGAGAAATGTTGAC
TTTATAGACACATGTTAAAAGATAAATAAAAAGTAAAAGATAAAAAAAATTAAAAATTAAAAA
TTAAAGGGAAAGAAGTAAAAAGAATGAGAGGTATGACATATTTTGAAAATATGTCCCTTG-
TTCTCTTTTTATTTTGATTATCTTTATTTTTTTAT-TTTTAATTTTAATTTTTATGTTTT-
AAGTTTTTTTTTATTTTTTACTTCTTTATAATGAATCTCAAA
>Brazil-11
TTAAGAGTGCACTTAATTGTGGCTTGAAATTTTACTT-ATTTACACCCAACGTC-
TTCGGGACACTGCGGC-
AATTTATTGCTTAGCGAATTTAACCCCTGCGGTTAGGCATATATAA-TTCTCTCT--
GAGGGTTGTATGTGTTCTAATCTTTGTTTTTTTTATTTT-
CAACCACAAATTTATACATATGTATATATGTATT-ATTTACTATCAAGTAAATAAATAT-
AAAACTTTTAACAATGGATCTCTTGGCTCTCA-
CATCGATGAAGAACACAGTGAAATGTGATAAGTAATGTGAATTGCAGAATTCAGTGAATCAT
CGAATTTTTGAACGCATATTGCGCCTTTTGGCTATTCCAAAAGGTACACCTGTTTGAGAGTA
TGAAAGGTCAGGGTGTTGAGAGAGTTATTAAAAAAAAAGAAAAAGGCAAAGTAACACTTTAA
GTGTTATTTTGTCTTTGATTTTTTTTTTTCTCTTAGCATCTTGGATATTGGGTG-
CTTGCCATTATTAAGTTTGATGG-
CTCACCTTAAATTTATAAGTTGTTTTTTTATTAAGGATGAAAAGTCTTTTGATGGCTTGATG
TTATTGA-
TATATGAAATGTCATTCATCAAGAAATCAGGGGGGTGACTAACCTTGATGAGAAATGTTGAC
TTTATAAACACATGTTAAAAGATAAATAAAAAGTAAAAGATAAAAAAAATAAAAAATAAAAAA
TTAAAGGGAGAGAAGTAAAAAGAATGGGAGGTATGACATATTTTGAAAATATGTCCCTTG-
TTCTCTTTTTATTTTGATTATCTTTATTTTTTTAT-TTTTAATTTTAATTTTTATGTTTT-
AAGTTTTATTTTATTTTTTACTTCTTTATAATGAATCTCAAA
>Brazil-12
TTAAGAGTGCACTTAATTGTGGCTCGAAATTTTACTT-ATTTACACCCAACGTC-
TTCGGGACACTGCGGC-
AATTTATTGCTTAGCGAATTTAACCCCTGCGGTTAGGCATATATAA-TTCTCTCT--
GAGGGTTGTATGTGTTCTAATCTTTGTTTTTTTTATTTT-
CAACCACAAATTTATACATATGTATATATGTATT-ATTTACTATCAAGTGAATAAATAT-
AAAACTTTTAACAATGGATCTCTTGGCTCTCA-
CATCGATGAAGAACACAGTGAAATGTGATAAGTAATGTGAATTGCAGAATTCAGTGAATCAT
CGAATTTTTGAACGCATATTGCGCCTTTTGGCTATTCCAAAAGGTACACCTGTTTGAGAGTA
TGAAAGGTCAGGGTGTTGAGAGAGTTATTAAAAAAAAAGAAAAAGGCAAAGTAACACTTTAA
GTGTTATTTTGTCTTTGATTTTTTTTTTTCTCTTAGCATCTTGGATATTGGGTG-
CTTGCCATTATTAAGTTTGATGG-
CTCACCTTAAATTTATAAGTTGTTTTTTTATTAAGGATGAAAAGTCTTTTGATGGCTTGATG
TTATTGA-
TATATGAAATGTCATTCATCAAGAAATCAGGGGGGTGACTAACCTTGATGAGAAATGCTGAC

TTTATAAACACATGTTAAAAGATAAATAAAAAGTAAAAGATAAAAAAAATAAAAAATAAAAAA
TTAAAGGGAAAGAAGTAAAAAGAATGAGAGGTATGACATATTTTGAAAATATGTCCCTTG-
TTCTCTTTTTATTTTGATTATCTTTATTTTTTTAT-TTTTAATTTTAATTTTTATGTTTT-
AAGTTTTATTTTATTTTTTACTTCTTTATAATGAATCTCAAA
>Brazil-13
TTAAGAGTGCACTTAATTGTGGCTTGAAATTTTACTTTATTTACACCCAACGTC-
TTCAGGACACTGCGGC-
AATTTATTGCTTAGCGAATTTAACCCCTGCGGTTAGGCATATATAA-TTCTCTCT--
GAGGGTTGTATGTGTTCTAATCTTTGTTTTTTTTATTTT-
CAACCACAAATTTATACATATGTATATATGTATT-ATTTACTATCAAGTAAATAAATAT-
AAAACTTTTAACAATGGATCTCTTGGCTCTCA-
CATCGATGAAGAACACAGTGAAATGTGATAAGTAATGTGAATTGCAGAATTCAGTGAATCAT
CGAATTTTTGAACGCATATTGCGCCTTTTGGCTATTCCAAAAGGTACACCTGTTTGAGAGTA
TGAAAGGTCAGGGTGTTGAGAGAGTTATTAAAAAAAAAGAAAAAGGCAAAGTAACACTTTAA
GTGTTATTTTGTCTTTGATTTTTTTTTTTCTCTTAGCATCTTGGATATTGGGTG-
CTTGCCATTATTAAGTTTGATGG-
CTCACCTTAAATTTATAAGTTGTTTTTTTATTAAGGATGAAAAGTCTTTTGATGGCTTGATG
TTATTGA-
TATATGAAATGTCATTCATCAAGAAATCAGGGGGGTGACTAACCTTGATGAGAAATGTTGAC
TTTATAAACACATGTTAAAAGATAAATAAAAAGTAAAAGATAAAAAAAATTAAAAATTAAAAA
TTAAAGGGAAAGAAGTAAAAAGAATGAGAGGTATGACATATTTTGAAAATATGTCCCTTG-
TTCTCTTTTTATTTTGATTATCTTTATTTTTTTAT-TTTTAATTTTAATTTTTATGTTTT-
AAGTTTTATTTTATTTTTTACTTCTTTATAATGAATCTCAAA
>Brazil-14
TTAAGAGTGCACTTAATTGTGGCTTGAAATTTTACTTTATTTACACCCAACGTC-
TTCGGGACACTGCGGC-
AATTTATTGCTTAGCGAATTTAACCCCTGCGGTTAGGCATATATAA-TTCTCTCT--
GAGGGTTGTATGTGTTCTAATCTTTGTTTTTTTTATTTT-
CAACCACAAATTTATACATATGTATATATGTATT-ATTTACTATCAAGTAAATAAATAT-
AAAACTTTTAACAATGGATCTCTTGGCTCTCA-
CATCGATGAAGAACACAGTGAAATGTGATAAGTAATGTGAATTGCAGAATTCAGTGAATCAT
CGAATTTTTGAACGCATATTGCGCCTTTTGGCTATTCCAAAAGGTACACCTGTTTGAGAGTA
TGAAAGGTCAGGGTGTTGAGAGAGTTATTAAAAAAAAAGAAAAAGGCAAAGTAACACTTTAA
GTGTTATTTTGTCTTTGATTTTATTTTTTCTCTTAGCATCTTGGATATTGGGTG-
CTTGCCATTATTAAGTTTGATGG-
CTCACCTTAAATTTATAAGTTGTTTTTTTATTAAGGATGAAAAGTCTTTTGATGGCTTGATG
TTATTGA-
TATATGAAATGTCATTCATCAAGAAATCAGGGGGGTGACTAACCTTGATGAGAAATGTTGAC
TTTATAAACACATGTTAAAAGATAAATAAAAAGTAAAAGATAAAAAAAAATAAAAAATAAAAA

TTAAAGGGAAAGAAGTAAAAAGAATGAGAGGTATGACATATTTTGAAAATATGTCCCTTG-
TTCTCTTTTTATTTTGATTATCTTTATTTTTTTAT-TTTTAATTTTAATTTTTATGTTTT-
AAGTTTTATTTTATTTTTTACTTCTTTATAATGAATCTCAAA
>Brazil-15
TTAAGAGTGCACTTAATTGTGGCTTGAAATTTTACTT-ATTTACACCCAACGTC-
TTCGGGACACTGCGGC-
AATTTATTGCTTAGCGAATTTAACCCCTGCGGTTAGGCATATATAA-TTCTCTCT--
GAGGGTTGTATGTGTTCTAATCTTTGTTTTTTTTATTTT-
CAACCACAAATTTATACATATGTATATATGTATT-ATTTACTATCAAGTAAATAAATAT-
AAAACTTTTAACAATGGATCTCTTGGCTCTCA-
CATCGATGAAGAACACAGTGAAATGTGATAAGTAATGTGAATTGCAGAATTCAGTGAATCAT
CGAATTTTTGAACGCATATTGCGCCTTTTGGCTATTCCAAAAGGTACACCTGTTTGAGAGTA
TGAAAGGTCAGGGTGTTGAGAGAGTTATTAAAGAAAAAGAAAAAGGCAAAGTAACACTTTAA
GTGTTATTTTGTCTTTGATTTTTTTTTTTCTCTTAGCATCTTGGATATTGGGTG-
CTTGCCATTATTAAGTTTGATGG-
CTCACCTTAAATTTATAAGTTGTTTTTTTATTAAGGATGAAAAGTCTTTTGATGGCTTGATG
TTATTGA-
TATATGAAATGTCATTCATCAAGAAATCAGGGGGGTGACTAACCTTGATGAGAAATGCTGAC
TTTATAAACACATGTTAAAAGATAAATAAAAAGTAAAAGATAAAAAAAAATAAAAAATAAAAA
TTAAAGGGAAAGAAGTAAAAAGAATGAGAGGTATGACATATTTTGAAAATATGTCCCTTG-
TTCTCTTTTTATTTTGATTATCTTTATTTTTTTAT-TTTTAATTTTAATTTTTATGTTTT-
AAGTTTTATTTTATTTTTTACTTCTTTATAATGAATCTCAAA
>Brazil-16
TTAAGAGTGCACTTAATTGTGGCTTGAAATTTTACTTTATTTACACCCAACGTC-
TTCGGGACACTGCGGC-
AATTTATTGCTTAGCGAATTTAACCCCTGCGGTTAGGCATATATAA-TTCTCTCT--
GAGGGTTGTATGTGTTCTAATCTTTGTTTTTTTTATTTT-
CAACCACAAATTTATACATATGTATATATGTATT-ATTTACTATCAAGTAAATAAATAT-
AAAACTTTTAACAATGGATCTCTTGGCTCTCA-
CATCGATGAAGAACACAGTGAAATGTGATAAGTAATGTGAATTGCAGAATTCAGTGAATCAT
CGAATTTTTGAACGCATATTGCGCCTTTTGGCTATTCCAAAAGGTACACCTGTTTGAGAGTA
TGAAAGGTCAGGGTGTTGAGAGAGTTATTAAAAAAAAAGAAAAAGGCAAAGTAACACTTTAA
GTGTTATTTTGTCTTTGATTTTTTTTTTTCTCTTAGCATCTTGGATATTGGGTG-
CTTGCCATTATTAAGTTTGATGG-
CTCACCTTAAATTTATAAGTTGTTTTTTTATTAAGGATGAAAAGTCTTTTGATGGCTTGATG
TTATTGA-
TATATGAAATGTCATTCATCAAGAAATCAGGGGGGTGACTAACCTTGATGAGAAATGCTGAC
TTTATAAACACATGTTAAAAGATAAATAAAAAGTAAAAGATAAAAAAAAATAAAAAATAAAAA
TTAAAGGGAAAGAAGTAAAAAGAATGAGAGGTATGACATATTTTGAAAATATGTCCCTTG-

TTCTCTTTTTATTTTGATTATCTTTATTTTTTTAT-TTTTAATTTTAATTTTTATGTTTT-
AAGTTTTATTTTATTTTTTACTTCTTTATAATGAATCTCAAA
>Brazil-17
TTAAGAGTGCACTTAATTGTGGCTTGAAATTTTACTTTATTTACACCCAACGTC-
TTCGGGACACTGCGGC-
AATTTATTGCTTAGCGAATTTAACCCCTGCGGTTAGGCATATATAA-TTCTCTCT--
GAGGGTTGTATGTGTTCTAATCTTTGTTTTTTTTATTTT-
CAACCACAAATTTATACATATGTATATATGTATT-ATTTACTATCAAGTAAATAAATAT-
AAAACTTTTAACAATGGATCTCTTGGCTCTCA-
CATCGATGAAGAACACAGTGAAATGTGATAAGTAATGTGAATTGCAGAATTCAGTGAATCAT
CGAATTTTTGAACGCATATTGCGCCTTTTGGCTATTCCAAAAGGTACACCTGTTTGAGAGTA
TGAAAGGTCAGGGTGTTGAGAGAGTTATTAAAAAAAAAGAAAAAGGCAAAGTAACACTTTAA
GTGTTATTTTGTCTTTGATTTTTTTTTTTTTCTTAGCATCTTGGATATTGGGTG-
CTTGCCATTATTAAGTTTGATGG-
CTCACCTTAAATTTATAAGTTGTTTTTTTATTAAGGATGAAAAGTCTTTTGATGGCTTGATG
TTATTGA-
TATATGAAATGTCATTCATCAAGAAATCAGGGGGGTGACTAACCTTGATGAGAAATGTTGAC
TTTATAAACACATGTTAAAAGATAAATAAAAAGTAAAAGATAAAAAAAATAAAAAATAAAAAA
TTAAAGGGAAAGAAGTAAAAAGAATGAGAGGTATGACATATTTTGAAAATATGTCCCTTG-
TTCTCTTTTTATTTTGATTATCTTTATTTTTTTAT-TTTTAATTTTAATTTTTATGTTTT-
AAGTTTTATTTTATTTTTTACTTCTTTATAATGAATCTCAAA
>Brazil-18
TTAAGAGTGCACTTAATTGTGGCTTGAAATTTTACTTTATTTACACCCAACGTC-
TTCGGGACACTGCGGC-
AATTTATTGCTTAGCGAATTTAACCCCTGTGGTTAGGCATATATAA-TTCTCTCT--
GAGGGTTGTATGTGTTCTAATCTT-GTTTTTTTTATTTT-
CAACCACAAATTTATACATATGTATATATGTATT-ATTTACTATCAAGTAAATAAATAT-
AAAACTTTTAACAATGGATCTCTTGGCTCTCA-
CATCGATGAAGAACACAGTGAAATGTGATAAGTAATGTGAATTGCAGAATTCAGTGAATCAT
CGAATTTTTGAACGCATATTGCGCCTTTTGGCTATTCCAAAAGGTACACCTGTTTGAGAGTA
TGAAAGGTCAGGGTGTTGAGAGAGTTATTAAAAAAAAAGAAAAAGGCAAAGTAACACTTTAA
GTGTTATTTTGTCTTTGATTTTTTTTTTTCTCTTAGCATCTTGGATATTGGGTG-
CTTGCCATTATTAAGTTTGATGG-
CTCACCTTAAATTTATAAGTTGTTTTTTTATTAAGGATGAAAAGTCTTTTGATGGCTTGATG
TTATTGA-
TATATGAAATGTCATTCATCAAGAAATCAGGGGGGTGACTAACCTTGATGAGAAATGTTGAC
TTTATAAACACAAGTTAAAAGATAAATAAAAAGTAAAAGATAAAAAAAATAAAAAATAAAAAA
TTAAAGGGAAGGAAGTAAAAAGAATGAGAGGTATGACATATTTTGAAAATATGTCCCTTG-
TTCTCTTTTTATTTTGATTATCTTTATTTTTTTAT-TTTTAATTTTAATTTTTATGTTTT-

AAGTTTTATTTTATTTTTTTACTTCTTTATAATGAATCTCAAA
